# Unusual Sesquiterpenes from *Streptomyces olindensis* DAUFPE 5622

**DOI:** 10.1021/acs.jnatprod.3c00752

**Published:** 2024-02-29

**Authors:** Fernanda O. Chagas, Leandro M. Garrido, Raphael Conti, Ricardo M. Borges, Vincent A. Bielinski, Gabriel Padilla, Mônica T. Pupo

**Affiliations:** † Faculdade de Ciências Farmacêuticas de Ribeirão Preto, 28133 Universidade de São Paulo, Ribeirão Preto, SP 05508-070, Brazil; ‡ Instituto de Pesquisas de Produtos Naturais Walter Mors, 28125 Universidade Federal do Rio de Janeiro, Rio de Janeiro, RJ 21941-617, Brazil; § Instituto de Ciências Biomédicas, 28133 Universidade de São Paulo, São Paulo, SP 05508-070, Brazil; ∥ Synthetic Biology and Bioenergy Group, J. Craig Venter Institute, La Jolla, California 92037, United States; ⊥ Instituto de Química, 28125 Universidade Federal do Rio de Janeiro, Rio de Janeiro, RJ 21941-617, Brazil

## Abstract

In nature, the vast majority of sesquiterpenes are produced by type I mechanisms, and glycosylated sesquiterpenes are rare in actinobacteria. *Streptomyces olindensis* DAUFPE 5622 produces the sesquiterpenes olindenones A–G, a new class of rearranged drimane sesquiterpenes. Olindenones B–D are oxygenated derivatives of olindenone A, while olindenones E–G are analogs glycosylated with dideoxysugars. ^13^C-isotope labeling studies demonstrated olindenone A biosynthesis occurs via the methylerythritol phosphate (MEP) pathway and suggested the rearrangement is only partially concerted. Based on the structures, one potential mechanism of olindenone A formation proceeds by cyclization of the linear terpenoid precursor, likely occurring via a terpene cyclase-mediated type II mechanism whereby the terminal alkene of the precursor is protonated, triggering carbocation-driven cyclization followed by rearrangement. Diphosphate hydrolysis may occur either before or after cyclization. Although a biosynthetic route is proposed, the terpene cyclase gene responsible for producing olindenones currently remains unidentified.

## Graphical abstract

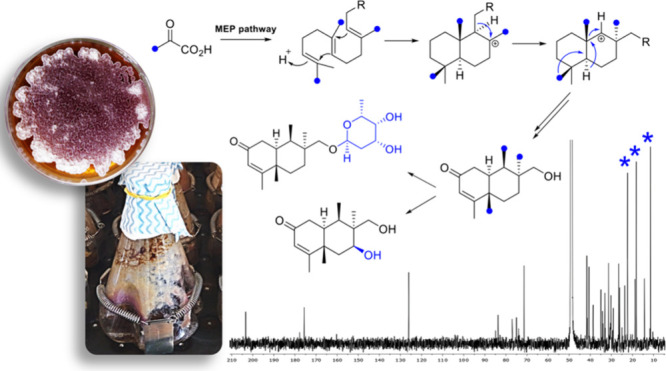

Terpenoids are molecules that comprise the most structurally and chemically diverse family of natural products currently known.^1^ Terpene synthases (TSs) are a group of carbon–oxygen lyases that cleave the phosphates from intermediates such as farnesyl diphosphate (FPP) and geranyl diphosphate (GPP), generating a carbocation that is eventually stabilized and quenched to synthesize structurally diverse terpene scaffolds. Terpene cyclases (TCs) are enzymes that catalyze regio- and stereoselective cyclizations upon the linear terpene scaffolds to generate a class of molecules with a highly diversified array of structures. Although TSs and TCs are often used interchangeably in scientific literature, not all TSs catalyze cyclization nor are all TCs phosphate lyases.

Although TCs are abundant throughout nature, only two mechanisms for initiating cyclization have been described: (i) diphosphate abstraction or (ii) protonation of an alkene or epoxide functional group. This conservation of enzyme activity between plant, bacterial, and fungal TCs with diverse amino acid sequences results from sharing highly similar structural folds and conserved metal cofactor-binding motifs. These metal-binding motifs, three-dimensional structures, functions, and specific mechanisms for producing the initial carbocation form the basis for dividing canonical TCs into type I or type II.^1–3^

During terpenoid cyclization, TCs may perform *cis–trans* isomerization and alkyl and hydride transfer or rearrangement. Some peculiar cyclization patterns and rearrangements have been reported, such as in merosesquiterpenes from sponges,^4–7^ which contain a rearranged drimane core; however, the absence of genome data from these organisms precludes the phylogenetic and biochemical analyses of these unique enzymes. On the other hand, mining the corresponding TC genes through genomic analysis may be challenging when the enzymes performing TC-like reactions are noncanonical.^2,8^ Also, predicting terpene structures from an identified TC is still unfeasible, which reinforces the need for unraveling some rules for terpenoid biosynthesis.^9–11^

Actinobacteria, especially *Streptomyces*, have long been explored because of their wide biosynthetic potential and are known producers of terpenoids, most notably the earthy odor-causing monoterpene derivative 2-methylisoborneol and degraded sesquiterpene geosmin.^12,13^ Other reports of cyclic sesquiterpene production in *Streptomyces* include the α-cadiene family,^14^ epi-isozizaene and albaflavenone,^15^ T-muurolol,^16^ bungoene, and pentalenene.^17^ It is important to note that a vast majority of the sesquiterpenes isolated from *Streptomyces* are synthesized by type I enzymes, as most of the cyclase genes identified via genome mining and biochemically characterized contain the classic type I metal cofactor-binding motifs. Recently, it was reported that *Streptomyces showdoensis* produces the sesquiterpene drimenol via a type II cyclase, which was identified using low-homology targeting of putative squalene-hopene cyclase genes.^18^ In addition, a bifunctional sesqui-TC isolated from marine bacteria that produces drimenol was biochemically characterized,^19^ but no homologues of these genes have been described in *Streptomyces* to date.

*Streptomyces* are free-living microorganisms or found in association with other organisms; they produce specialized metabolites for environmental signaling and symbiotic interactions, metabolites that may also have biological and pharmacological interest. The actinobacterium *Streptomyces olindensis* DAUFPE 5622 produces the antitumor cosmomycins, glycosylated anthracyclines with trisaccharide chains attached to C-7 and/or C-10.^20^ These saccharides, composed mainly of deoxysugars, possess structural variability that confers wide diversity to cosmomycins.^20^ In addition to the biosynthetic gene cluster (BGC) involved in cosmomycins biosynthesis, 34 BGCs were predicted in the genome of *S. olindensis* DAUFPE 5622 by antiSMASH,^21^ many of which are still cryptic. Of these 34 putative BGCs, four were predicted to encode the terpenes geosmin, albaflavenone, α-amorphene, and squalene, while the rest lacked strong identity with known terpenoid BGCs, highlighting the potential of this bacterial strain for the biosynthesis of new natural products.

Herein, we report the isolation and structural elucidation of the novel sesquiterpenes olindenones A–G (**1**–**7**), as well as the results of labeling studies that suggest peculiarities in their biosynthesis.

## Results and Discussion

Olindenone A (**1**) was the major sesquiterpene produced in the experimental growth conditions of *S. olindensis* DAUFPE 5622 and is a possible biosynthetic precursor of olindenones B–G (**2**–**7**) (Figure 1). The structure of **1** was elucidated based on 1D and 2D NMR data and confirmed by HRMS (Tables 1 and S1, Figures S1–S8). In brief, interpretation of the NMR spectra revealed 15 carbons, including four methyls (δ_C_ 11.4, 18.0, 18.7, and 21.9), a hydroxymethyl (δ_C_ 71.5), two olefinic carbons (δ_C_ 125.9 and 171.5), and a carbonyl (δ_C_ 200.6). In addition, methylene (δ_C_ 25.6, 30.4, and 40.0), methine (δ_C_ 39.2 and 40.2), and quaternary (δ_C_ 37.8 and 39.9) carbons were observed. The proton multiplicity together with *g*COSY and *g*HMBC data (Figure 2) permitted assembly of the carbon skeleton. The structure is a decalin-type bicyclic skeleton featuring an α,β-unsaturated carbonyl and containing one hydroxymethyl (C-14) and four methyls (C-11, C-12, C-13, and C-15), of which one is attached to the olefinic β-carbonyl carbon C-4, one is attached to the methine carbon C-9, and two are attached to the quaternary carbons C-5 and C-8, respectively.

**Figure 1:**
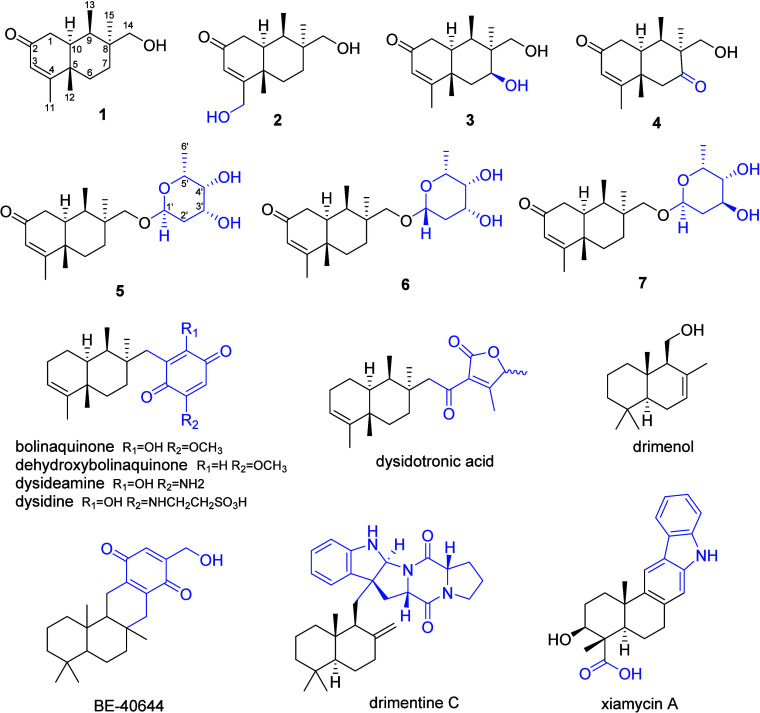
Olindenones A–G (**1**–**7**) and structurally related compounds. The rearranged and nonrearranged drimane scaffolds are drawn in black. For **5**–**7** the relative configurations for the sesquiterpene and for the sugars are depicted, but the overall relative and absolute configurations remain to be determined.

**Figure 2:**
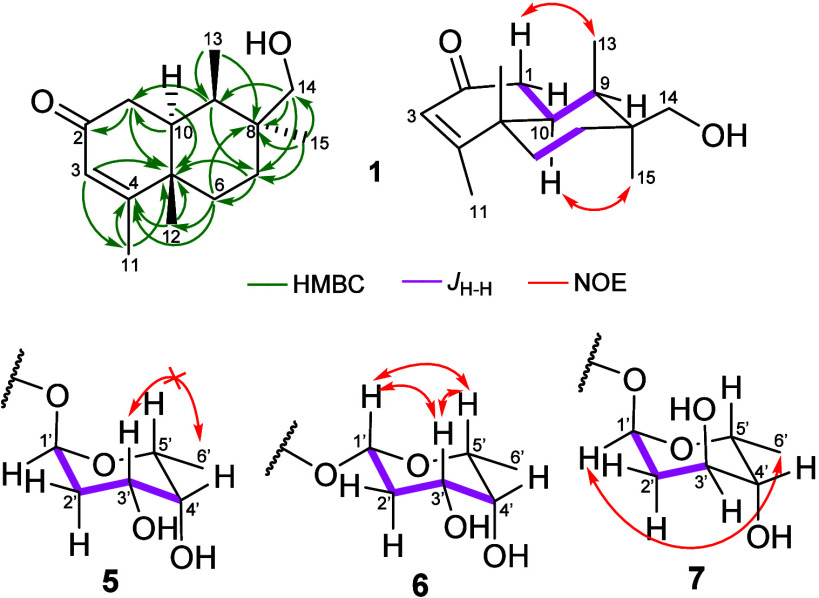
Key NMR correlations for assigning olindenone structures.

**Table 1:** NMR Data of Olindenones A–G (**1**–**7**)

		olindenone A (**1**)			olindenone B (**2**)[Table-fn t1fn5]		olindenone C (**3**)		olindenone D (**4**)		olindenone E (**5**)		olindenone F (**6**)		olindenone G (**7**)	
		δ_C_ ^b^	δ_C_, type^c^	δ_H_, mult. (*J* in Hz)^c^	_C_ ^c^	δ_H_, mult. (*J* in Hz)^c^	δ_C_ ^b^	δ_H_, mult. (*J* in Hz)^b^	δ_C_ ^b^	δ_H_, mult. (*J* in Hz)^b^	δ_C_ ^c^	δ_H_, mult. (*J* in Hz)^c^	δ_C_ ^b^	δ_H_, mult. (*J* in Hz)^b^	δ_C_ ^b^	δ_H_, mult. (*J* in Hz)^b^
1	α	41.3	40.0, CH_2_	2.04, dd (17.0, 3.3)	39.9	2.10, dd (17.1, 3.1)	40.3	2.03, dd (17.2, 4.0)	39.5	2.22, m	39.9	2.03, dd (16.9, 2.7)	40.4	1.99, dd (17.1, 3.5)	40.7	1.97, dd (16.9, 3.5)
	β			2.60, dd (17.0, 14.5)		2.65, dd (17.1, 14.5)		2.78, dd (17.2, 14.5)		2.73, dd (17.0, 14.3)		2.59, dd (16.9, 14.5)		2.67, dd (17.1, 14.7)		2.67, dd (16.9, 14.7)
2		203.3	200.6, C		200.4		203.4		202.0		200.4		203.4		n.o.	
3		126.1	125.9, CH	5.71, br s	121.8	6.08, br s	125.2	5.66, br s	126.2	5.78, br s	126.0	5.71, br s	125.9	5.70, br s	125.9	5.69, br s
4		175.6	171.5, C		172.3		176.4		172.1		171.6		175.5		176.0	
5		40.8	39.9 C		39.2		41.4		45.7		39.9		41.1		41.1	
6	a	31.4	30.4, CH_2_	1.67, m	29.5	1.54–1.69, m^d^	38.5	2.00, dd (14.2, 2.3)	47.8	2.93, d (12.9)	30.4	1.65, m (overlapped)	30.7	1.63, m	31.1	1.69, m
	b			1.56, m		1.25, m^d^		1.80, dd (14.2, 3.1)		2.30, d (12.9)		1.53, m (overlapped)		1.30, m^d^		1.60, m
7	a	26.6	25.6, CH_2_	1.57, m	25.3	1.54–1.69, m^d^	74.2, CH	3.81, m	217.9, C		26.0	1.58, m (overlapped)	26.3	1.62, m	26.6	1.65, m
	b			1.17, m		1.17, m^d^						1.24, m		1.18, m		1.21, m
8		38.6	37.8, C		37.5		42.2		54.0		37.1		37.5		37.9	
9		40.6	39.2, CH	1.54, m	38.9	1.54, m	40.4	1.60, dq (7.3, 4.0)	44.2	2.07, m	39.7	1.51, m (overlapped)	40.7	1.55, m	40.8	1.49, m
10		41.7	40.2, CH	2.37, dt (14.5, 3.3)	40.3	2.43, ddd (14.4, 4.2, 3.5)	41.5	2.48, dt (14.5, 4.0)	41.0	3.01, dt (14.1, 3.9)	40.2	2.38, dt (14.5, 2.7)	41.3	2.38, dt (14.7, 3.5)	41.5	2.39, dt (14.7, 3.5)
11	a	18.8	18.7, CH_3_	1.88, br s	60.4, CH_2_	4.40, dd (17.3, 1.6)	18.7	1.95, br s	18.3	1.92, br s	18.5	1.88, br s	18.5	1.93, d (1.1)	18.5	1.93, d (1.1)
	b					4.35, dd (17.3, 1.6)										
12		18.2	18.0, CH_3_	1.09, s	19.0	1.17, s	20.6	1.42, s	19.5	1.09, s	18.0	1.08, s	17.8	1.13, s	17.9	1.13, s
13		11.5	11.4, CH_3_	0.97, d (7.4)	11.1	0.98, d (7.7)	12.0	1.21, d (7.3)	11.6	0.98, d (7.7)	11.4	0.93, d (7.5)	11.2	0.97, d (7.7)	11.2	1.00, d (7.7)
14	a	71.4	71.5, CH_2_	3.54, d (10.6)	71.3	3.54, d (10.7)	68.1	3.80, d, (10.5)^d^	64.8	3.70, d (11.3)	77.0	3.52, d (9.4)	78.3	3.59, d (9.1)	77.9	3.71, d (9.4)
	b			3.29, d (10.6)		3.29, d (10.7)		3.67, d (10.5)^d^		3.64, m (overlapped)		2.96, d (9.4)		3.28, d (9.1)		2.99, d (9.4)
15		22.3	21.9, CH_3_	1.10, s	21.8	1.11, s	23.1	1.04, s	22.4	1.42, s	22.6	1.08, s	22.6	1.12, s	22.6	1.09, s
1′											99.2, CH	4.84, d (3.4)	101.9	4.41, dd (9.8, 1.5)	105.9	5.13, dd (5.3, 1.5)
2′	a										33.2, CH_2_	1.77, td (12.5, 3.4)	35.1	1.70, m	42.7	2.02, ddd (13.1, 6.9, 5.3)
	b											1.94, dd (12.5, 5.1)		1.80, m		2.19, ddd (13.1, 5.2, 1.5)
3′											66.0, CH	4.02, m^c^ or	69.8	3.68, dt (12.4, 3.0)	72.8	4.24, dt (6.9, 5.2)
												3.92, ddd (13.5, 5.0, 2.0)^b^				
4′											71.4, CH	3.64, br s	71.2	3.45, br s	91.0	3.57, dd (6.4, 5.2)
5′											66.2, CH	3.91, q (6.7)	71.7	3.50, q (6.4)	70.6	3.68, quint (6.4)
6′											16.7, CH_3_	1.28, d (6.7)	16.7	1.27, d (6.4)	19.0	1.19, d (6.4)

a Acquired at 500 MHz in CD_3_OD.

b Acquired at 500 MHz in CDCl_3_. α and β refer to orientation, while a and b distinguish each geminal hydrogen.

c Imprecise chemical shift or *J* measurement due to impurities and low resolution. Not observed signal (n.o.).

d ^13^C-enriched compound.

The relative configuration, including *trans*-fusion of the decalin system, was deduced based on the key ^1^H–^1^H coupling constants of H-10 with H-1β (*J* = 14.5 Hz) and H-9 (*J* = 3.3 Hz) and from NOEDIFF correlations, including H-13 with H-1β and H-10 with H-15 (Figure 2). In this case, the absence of a correlation between H-10 and H-12 also supports the *trans*-ring fusion. Therefore, methyls C-12 and C-13 must be in axial positions on the same face (β-oriented), while methyl C-15 and methine proton H-10 are axially oriented to the α-plane (Figure 2). HRMS revealed a sodium adduct at *m*/*z* 259.1677 [M + Na]^+^ (calcd for C_15_H_24_O_2_Na^+^ 259.16685, Δ 3.3 ppm).

After structural elucidation of **1**, HPLC purification of additional sesquiterpene analogs from the *S. olindenses* DAUFPE 5622 extracts was performed making use of the α,β-unsaturated carbonyl at λ_max_ 241 nm (Figure S9).

In the ^1^H NMR spectrum of olindenone B (**2**) the signal of methyl protons H-11 in (**1**) was replaced by signals of two hydroxymethyl protons H-11a/b (δ_H_ 4.40 and 4.35). These protons are more deshielded than the hydroxymethyl protons H-14a/b (δ_H_ 3.54 and 3.29). The methine proton H-3 (δ_H_ 6.08) was also more deshielded in **2** relative to **1** (see Table S2, Figures S10–S14 for complete NMR data). Olindenone C (**3**) featured an additional oxymethine proton H-7 (δ_H_ 3.81) and deshielded methylene protons H-6a/b (δ_H_ 2.00 and 1.80), methyl protons H-12 (δ_H_ 1.42) and H-13 (δ_H_ 1.21), and hydroxymethyl protons H-14a/b (δ_H_ 3.80 and 3.67) (Table S3, Figures S16–S19). The ^1^H NMR spectrum of olindenone D (**4**) presented proton signals that were deshielded compared to the corresponding signals in **1**, such as H-6a/b (δ_H_ 2.93 and 2.30), H-9 (δ_H_ 2.07), H-10 (δ_H_ 3.01), and H-15 (δ_H_ 1.42). However, no additional or missing signals were observed in the ^1^H NMR spectrum due to the very low concentration of **4** in the impure analysis sample (Table S4, Figures S21–S24). *g*HSQC, *g*HMBC, and *g*COSY spectra were acquired to complete assignments of **2**–**4**. The NOESY experiment of **2** was performed to confirm that the relative configuration was the same as in **1**. The relative C-7 configuration of **3** was ascribed based on the coupling constant of H-7 with H-6a (*J* = 2.3 Hz) and H-6b (*J* = 3.1 Hz), indicating H-7 is equatorial, which implies the axial position of the hydroxy group at C-7. The HRMS of **2**, **3**, and **4** revealed protonated molecules at *m*/*z* 253.1791 [M + H]^+^ (calcd for C_15_H_25_O_3_^+^, 253.17982, Δ 2.8 ppm), *m*/*z* 253.1801 [M + H]^+^ (calcd for C_15_H_25_O_3_^+^, 253.17982, Δ 1.1 ppm), and *m*/*z* 251.1652 [M + H]^+^ (calcd for C_15_H_23_O_3_^+^, 251.16417, Δ 4.1 ppm), respectively.

^1^H NMR spectra of olindenones E–G (**5**–**7**) presented signals very similar to **1** plus additional signals consistent with sugar moieties. Complete carbon and proton assignments were obtained from *g*HSQC, *g*HMBC, *g*COSY, and *g*TOCSY spectra, revealing that **5**–**7** are glycosylated derivatives of **1** containing very similar 2′,6′-dideoxysugars which differ in their relative configurations. Those configurations were deduced from ^1^H–^1^H coupling constants and NOESY correlations (Figure 2). The sesquiterpene backbone in olindenone E (**5**) is linked to the sugar to generate an axial-oriented substituent at anomeric carbon C-1′. The relative configuration was deduced from ^1^H–^1^H coupling constants (Table S5, Figures S26–S31). The coupling constant between H-1′ and H-2′α (*J* = 3.4 Hz) indicates that H-1′ is equatorial. H-2′α also presents geminal and *trans*-diaxial *J* values of 12.5 Hz each, indicating H-3′ is axial (and thus the hydroxy at C-3′ is equatorial). Because of the unresolved *J*_H‑3_ in CDCl_3_, ^1^H NMR was also acquired in CD_3_OD, affording a resolved signal at δ_H_ 3.92 (ddd, *J* = 13.5, 5.0, and 2.0 Hz) and confirming *trans*-axial relation between H-3′ and H-2′α (*J* = 13.5 Hz). In CDCl_3_, the H-4′ signal appears as a broad singlet due to the small coupling constant with H-3′, revealing the equatorial orientation of H-4′ (and thus the axial hydroxy at C-4′). The small coupling constant between H-4′ and H-5′ precluded determining their relationship as *trans*-diequatorial or equatorial–axial. For this purpose, NOE experiments were performed. An NOE between the axial H-3′ and the axial substituent at position 5′ (methyl or proton) was expected but was not observed. One plausible reason is the resonance frequency similarities, translated as chemical shift proximities of H-3′ (δ_H_ 4.02) and H-5′ (δ_H_ 3.91) signals in ^1^H NMR spectrum that precluded accurate nucleus irradiation, suggesting both nuclei were irradiated at the same time during the NOE experiment. On the other hand, a methyl group in the axial position should show an NOE with H-3′; thus, we concluded that the absence of an NOE suggests that methyl C-6′ is equatorial whereas H-5′ is axial. HRMS of **5** revealed a sodium adduct at *m*/*z* 389.2289 [M + Na]^+^ (calcd for C_21_H_34_O_5_Na^+^, 389.22985, Δ 2.4 ppm).

The dideoxysugar in olindenone F (**6**) is attached to the sesquiterpene via a β-linkage at C-1′. Similar to **5**, substituent orientations were deduced from ^1^H–^1^H coupling constants and NOESY correlations (Figure 2). The main difference was the coupling constant between H-1′ and H-2′ (*J* = 9.8 Hz) indicating *trans*-diaxial coupling. NOEs between H-1′, H-3′, and H-5′, together with their *J* values, indicated these protons were axially oriented on the same face of the molecule. Thus, the relative configuration of the sugar was determined (Table S6, Figures S32–S38). HRMS of **6** revealed a protonated molecule and sodium adduct at *m*/*z* 367.2465 [M + H]^+^ (calcd C_21_H_35_O_5_^+^, 367.24790, Δ 3.8 ppm) and *m*/*z* 389.2282 [M + Na]^+^ (calcd C_21_H_34_O_5_Na^+^, 389.22985, Δ 4.2 ppm), respectively.

Olindenone G (**7**) has an α-linked sugar (Table S7, Figures S40–S45). Orientations of substituents at positions 1′ and 3′ were deduced based on coupling constants (*J*_1′,2′a_ = 5.3 Hz; *J*_2′b,3′_ = 5.2 Hz) which suggest H-1′ and H-3′ are equatorial (Figure 2). Thus, unlike in **5** and **6**, the hydroxy at C-3′ in **7** is axial. Determining C-4′ and C-5′ relative configurations from *J* values was unfeasible; however, the *J* values preclude a *trans*-diaxial coupling between protons H-4′ and H-5′. One possibility would be an axial H-4′, which implies an equatorial H-5′ and axial C-6′ methyl. In that case, all sterically bulky substituents (OR-1′, OH-3′, OH-4′, and C-6′, R = the sesquiterpene backbone) would be on the same side of the molecule, and three of them, including methyl C-6′, with axial positions. In this case, the sugar ring most likely would undergo a ring flip to the more thermodynamically stable conformation, leading to the same structure as **6**. We therefore concluded that H-4′ in **7** must be equatorial. Lastly, an NOE between H-1′ and methyl H-6′ showed they are on the same face, suggesting the methyl is equatorial and H-5′ axial. Together with the fact that the terpenoid substituent OR at C-1′ has the preferred axial position according to the well-known anomeric effect, the chair conformation proposed for this sugar is stable even with three groups (OR-1′, OH-3′, and OH-4′) in axial positions. The ion *m*/*z* 389.2310 [M + Na]^+^ (calcd for C_21_H_34_O_5_Na^+^, 389.22985, Δ 3.0 ppm) detected in the HRMS refers to the sodium adduct of **7**.

While olindenones B–D (**2**–**4**) are oxygenated analogs of **1**, olindenones E–G (**5**–**7**) are 14-*O*-glycosylated analogs. **6** is a C-1′ epimer of **5** (anomer in this case), while **7** is a C-2′ epimer of **5**. The relative configuration was established experimentally for the terpene core and for the sugar moiety of the glycosylated analogs, but not across the glycosidic bond. The overall relative configuration for **5**–**7** and the absolute configurations for **1**–**7** remain to be determined.

Compared to drimane sesquiterpenes, the methyl groups in the decalin system of olindenones are positioned differently. Based on related structures previously reported,^2,5–8,22,23,27,28,34^ including drimane derivatives and rearranged drimanes, we propose the biosynthetic route of olindenone A (**1**) (Figure 3). The predicted steps for the biosynthesis of the sugars^25^ found in olindenones E–G (**5**–**7**) are in the Supporting Information (Figure S47). Protonation of the alkene in the intermediate FPP (or, alternatively, farnesol) could generate the initial tertiary carbocation, consistent with a type II mechanism,^2,8,27,28,34^ which is rare in the biosynthesis of sesquiterpenes. Subsequent carbocation-directed cyclization could form a *trans*-fused bicyclic core that undergoes a rearrangement.^5,7^ The suprafacial migration of hydride and alkyl groups leads to a backbone conformational flip to maintain the thermodynamic stability of the molecule (Figure 3). Olindenones A–G (**1**–**7**) may be biosynthesized via a 4,8-drimane rearrangement, in which an alkyl migration from position 9 to 8 occurs instead of the 9,8-hydride shift observed in the previously reported 4,9-*friedo*-drimane rearrangement^6,7,22,23^ (Figure 3).

**Figure 3:**
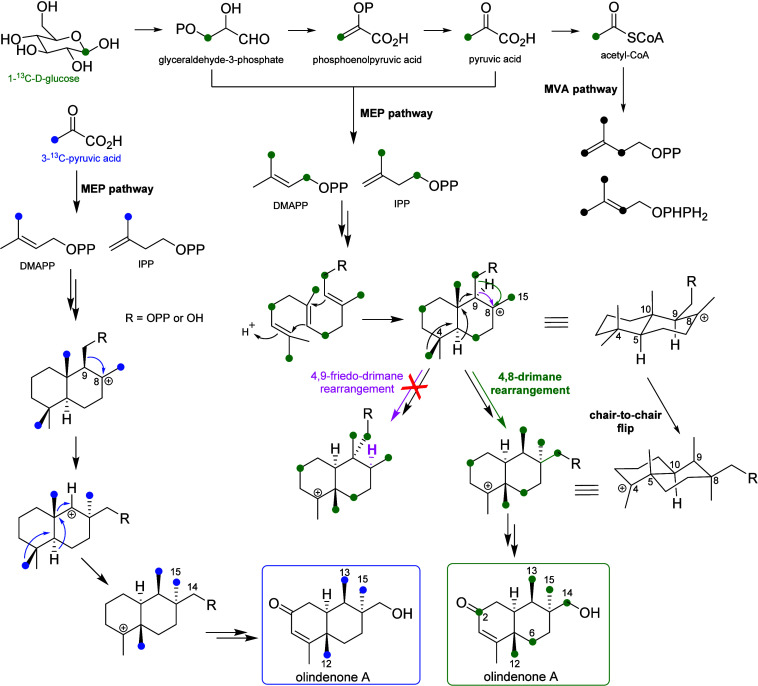
Labeling experiments and chemical reactions involved in the proposed biosynthesis of olindenone A (**1**). Observed labeling pattern for glucose-1-^13^C (green) and pyruvate-3-^13^C (blue). The difference between a 4,9-*friedo*-rearrangement (pink) and a 4,8-drimane rearrangement is shown.

A 4,8-drimane rearrangement has only been previously described in a very small set of sponge meroterpenes (bolinaquinone, dehydroxybolinaquinone, dysideamine, dysidine, and dysidotronic acid, Figure 1).^4–7,24^ Thus, olindenones A–G (**1**–**7**) may be the first reported 4,8-drimane rearranged nonhybrid sesquiterpenes. Bacterial glycosylated sesquiterpenes, such as **5**–**7**, are also very rare, with only one previously reported example.^29–31^

The primary alcohol in olindenones most likely derives from the diphosphate hydrolysis that may occur either before or after the rearrangement. In some nonrearranged drimane meroterpenoids the FPP cyclizes after being assembled to the polyketide moiety.^26–28^ In those cases, diphosphate lysis occurs before cyclization to generate the carbocation that is transferred to a nucleophile for prenylation.^2^ However, in drimenol (Figure 1), a nonrearranged drimane sesquiterpene reported in bacteria, the cyclization takes place before phosphate cleavage.^18,19^

Usually, the methylerythritol phosphate (MEP) pathway is the main biosynthetic route for the bacterial terpenes, but both MEP and mevalonate pathway (MVA) pathways have been observed in *Streptomyces*.^32^ Indeed, genes for both pathways were identified in *S. olindensis* DAUFPE 5622. In order to determine the precursor pathway producing olindenones, *S. olindenses* DAUFPE 5622 was cultured in a medium supplemented with labeled glucose-1-^13^C. NMR analyses of biosynthetically labeled **1** showed a ^13^C-enrichment pattern consistent with biosynthesis via the MEP pathway, yielding labeled C-2, C-6, C-12, C-13, C-14, and C-15 (Figures 3 and S48, Table S8).

Although this experiment established the involvement of the MEP biosynthetic pathway, the complete mechanism for the rearrangement was still unclear. Both C-14 and C-15 were labeled when the medium was supplemented with glucose-1-^13^C and the origin of C-14 hydroxylation was uncertain (Figure 3). A second labeling experiment, using sodium pyruvate-3-^13^C, was performed to determine which methyl group had rearranged from position 9 to 8 through observation of the C-14 and C-15 labeling. In addition, the final orientation (α or β) of the group undergoing a suprafacial shift would provide further evidence for the mechanism of rearrangement. In this experiment, C-12, C-13, and C-15 were labeled (Figures 3 and S49, Table S8). The β-oriented C-14 of olindenone A (**1**) was unlabeled, indicating this was the methyl group shifted from position 9. This labeling result is also consistent with the oxygen at C-14, which presumably originated from the diphosphate at C-9 (Figure 3). No experimental evidence has been obtained to distinguish whether diphosphate hydrolysis occurs before or after rearrangement. In summary, we propose that the shift of the oxidized methyl from position 9 to 8 is the first step in the rearrangement, prompting the subsequent concerted 10,9-methyl, 5,10-hydride, and 4,5-methyl shifts. Presumably, the 9,8-methyl shift occurs first because the simultaneous shift of methyls C-14 and C-13 on the same face is disfavored. The reason for the preferential shift of the 9-methyl over the 9-hydride, which happens in the 4,9-*friedo*-drimane rearrangement,^6,7,22,23^ is unclear, but 4,9-rearranged compounds were not detected in the *S. olindensis* DAUFPE 5622.

For mining the olindenone BGC in the *S. olindensis* DAUFPE 5622 genome, we identified candidate biosynthetic enzymes, based on our proposed biosynthetic route. Conversion of FPP (or farnesol) to the 4,8-rearranged drimane is likely performed by the same TC. Diphosphate hydrolysis to generate the primary alcohol may be mediated by a hydrolase, as reported before in *Streptomyces showdoensis*,^18^ or alternatively by some exotic TC like the bifunctional enzyme that produces drimenol in marine bacteria.^19^ A monooxygenase might act on the 3,4-unsaturated sesquiterpene intermediate to generate an allylic hydroxy, which is subsequently converted to the α,β-unsaturated ketone of **1** (Figure 3). Tailoring enzymes, such as monooxygenases and glycosyltransferases could act on **1** or on a related intermediate to form the other olindenones, or **1** might be a hydrolysis product of **5**–**7**.

While the vast majority of known bacterial sesqui-TCs operate through a type I mechanism,^2,33^ olindenones appear to be biosynthesized via type II cyclization. Relatively similar compounds, e.g., bacterial nonrearranged drimane merosesquiterpene BE-40644, drimentines, and xiamycins (Figure 1), are biosynthesized by noncanonical sesqui-TCs^2,8,27,28,34^ that are integral membrane cyclases lacking the aspartate-rich motif typically found in type I and type II TCs. On the other hand, the nonrearranged drimane sesquiterpene drimenol (Figure 1), the closest relative of **1**, is produced in *Streptomyces showdoensis* by a canonical type II TC containing a β,γ-didomain architecture^18^ with the subsequent action of a genetically clustered hydrolase. Drimenol is also produced in marine bacteria^19^ and fungi^35^ by bifunctional TCs, which cyclize the FPP by a type II mechanism and hydrolyze its phosphate by a type I-like reaction. Alternatively, fungi produce drimenol through a noncanonical type II TC and a hydrolase separately,^36^ while plants use canonical type I TCs.^37^

In order to predict the putative genes involved in olindenone biosynthesis, antiSMASH analysis was performed.^38^ Five putative terpene BGCs were found (Table S9): a geosmin BGC (100% similarity, contig JJOH01000005.1); an albaflavenone BGC (100% similarity, contig JJOH01000108.1); a hopene BGC (84% similarity, contig JJOH01000108.1); a putative undecaprenyl diphosphate synthase family protein (contig JJOH01000092.1) potentially involved in peptidoglycan biosynthesis; and a predicted terpene BGC (contig JJOH01000001.1), which contains a gene for a polyprenyl synthase (DF19_00385). We identified in this BGC a gene for a noncanonical TC (DF19_00380, KDN80181.1), not predicted by antiSMASH, with some similarity to the integral membrane cyclases producing nonrearranged meroterpenes that would be a candidate TC for the olindenone biosynthesis (Figure S50). However, this BGC contains other genes whose functions are unrelated to olindenone production, suggesting this may not be the olindenone BGC. Apart from the antiSMASH results, we identified a gene for a putative sesqui-TC predicted to biosynthesize α-amorphene (DF19_12680). We did not find a TC gene similar to those in the *Streptomyces* sp. producer of drimenol nor any other putative BGC that fits the minimal requirement for olindenone production; thus, the bioinformatic analysis was insufficient to predict the olindenone BGC.

Compound **1** was the only olindenone isolated in sufficient yield for preliminary biological testing, while the other olindenones yielded 1 mg or less. Compound **1** was not cytotoxic against colon adenocarcinoma tumor cells and was inactive in antimicrobial assays against *Escherichia coli*, *Bacillus subtilis*, and *Candida albicans*.

The 4,8-drimane rearranged sesquiterpenes are rare, and their BGCs and biological functions are still cryptic. Data presented herein shed light on some aspects of their peculiar biosynthesis and pave the way for elucidating the enzymes and genes responsible for producing olindenones and other 4,8-drimane rearranged analogs.

## Experimental Section

### General Experimental Procedures

Optical rotation was recorded in a P-2000 digital polarimeter (Jasco) using methanol. 1D and 2D NMR analyses were performed at 500 MHz (Bruker Avance DRX-500 spectrometer) using deuterated chloroform or methanol. Some NMR analyses were performed or repeated at 800 MHz (Bruker 800 MHz Avance III spectrometer equipped with a 1.7 mm TCI cryoprobe) using 40 μL of deuterated methanol (Cambridge Isotope Laboratories) and pulse sequences zg30 for ^1^H data acquisition, cosygpppqf for COSY data acquisition, hsqcetgpprsisp2.3 for HSQC data acquisition, hmbcetgpl3nd for HMBC data acquisition, and noesygpphpp for NOESY data acquisition. The NMR spectra were referenced to solvent peaks of chloroform (δ_H_ 7.26, δ_C_ 77.2) or methanol (δ_H_ 3.31, δ_C_ 49.0). The MS system was a quadrupole time-of-flight instrument (UltrOTOF-Q, Bruker Daltonics) or a triple-quadrupole (XEVO TQ-S, Waters Corporation), both equipped with an ESI ion source. ESI-MS of purified fractions containing the compounds was performed using a capillary voltage of 3900 V, a dry gas flow of 4 L h^–1^, and nitrogen nebulizer gas.

HPLC analysis was performed in an HPLC system (Shimadzu) with an LC-6AD solvent pump, an SCL 10AVB system controller, a CTO-10ASVP column oven, a Rheodyne model 7725 injector, an SPD-M10AVP diode array detector (DAD), and Class-VP software for data acquisition. An analytical Gemini C6-phenyl column (100 mm, 4.6 mm, 5 μm, Phenomenex) coupled to a precolumn (4.0 mm × 4.6 mm, Phenomenex) was used with gradient aqueous acetonitrile (MeCN) mobile phase at a flow rate of 1.0 mL min^–1^ for 30 min. The mobile phase was 10% MeCN for 4 min, linear gradient from 10% to 25% MeCN in 9 min, 25% MeCN for 7 min, gradient from 25% to 100% MeCN in 3 min, 100% MeCN for 3 min, gradient from 100% to 10% MeCN in 1 min, and 10% MeCN for 3 min. HPLC purifications were carried out with a semipreparative C6-phenyl column (250 mm, 10.0 mm, 5 μm, Phenomenex) attached to a guard column (10.0 mm, 10.0 mm, 5 μm) using aqueous MeCN at 3.0 mL min^–1^. Per run, 200 μL of each sample at 15–20 mg mL^–1^ was purified using a suitable aqueous acetonitrile gradient. UV profiles of compounds were observed in the HPLC-DAD.

### *S. olindensis* DAUFPE 5622 strain and bioinformatic analysis

The actinobacterium DAUFPE 5622, identified as *Streptomyces olindensis*, was isolated from soil in Northeast Brazil in the 1960s^39,40^ and has been preserved as part of the microbial collection at Institute of Biomedical Sciences, University of São Paulo, São Paulo, SP, Brazil. Permission for studying this microorganism is granted through SisGen under registration number A4CA5B3.

The whole-genome shotgun sequence is available at NCBI under accession number JJOH00000000.1. DNA and Protein IDs used for BGC prediction are available in NCBI. The prediction of putative terpene BGCs was made using the bacterial version of antiSMASH 6.1.1,^38^ using the default settings. Web tools available at the Enzyme Function Initiative Web site were used to create the Sequence Similarity Network (SSN). The visual diagram of the network was created with Cytoscape 3.9.1.

### Microbial Culture and Extraction

The stored strain *S. olindensis* DAUFPE 5622 was reactivated in a 50 mL Falcon tube with 10 mL of SFM liquid medium (20 g of soy flour and 20 g of mannitol per 1 L of deionized water) at 200 rpm and 30 °C for 48 h. The liquid culture was plated on SFM agar (20 g of agar per 1 L of SFM) using a swab, and the plates were incubated unsealed at 30 °C for 7 d. One 5 mm diameter plug of the actinobacterium agar culture was transferred to a 500 mL baffled Erlenmeyer flask containing 50 mL of liquid SFM and incubated on a rotary shaker at 30 °C and 200 rpm for 11 d. The microbial culture with the actinobacterium mycelia was extracted with 80 mL of ethyl acetate (2 × 40 mL) per 50 mL of culture. The organic solvent was separated through a liquid–liquid partition and evaporated under low pressure to obtain the extract.

For labeling experiments, the same conditions for culture and extraction were used, except that the 500 mL baffled Erlenmeyer flasks contained 50 mL of a modified soy flour medium (20 g of soy flour and 4 g of dextrose per 1 L of deionized water, named SFd medium) and the labeled precursor (see below).

### Isolation and Analysis of Olindenones A–G

The extract (∼600 mg) derived from 20 flasks (1000 mL) of a SFM liquid culture was prefractionated using an SPE cartridge (C18, 10 g, 60 mL) eluted with 120 mL of water, 60 mL of 25% aqueous methanol (MeOH), 60 mL of 50% MeOH, 60 mL of 75% MeOH, and 120 mL of MeOH, and five fractions were obtained. The extract and fractions were analyzed by HPLC using the analytical conditions described above. Fractions 3 and 4 were purified using an HPLC semipreparative column (see general conditions above).

Fraction 4 (4.5 mg) was fractionated using 20% aqueous MeCN for 22 min, 20% to 30% MeCN in 1 min, 30% MeCN for 12 min, followed by column cleaning. Olindenones A (**1**, 1.3 mg), G (**7**, 0.2 mg), E (**5**, 0.8 mg), and F (**6**, 0.2 mg) were collected from 21.0 to 22.6 min, 26.0 to 26.8 min, 26.9 to 28.4 min, and 29.8 to 31.4 min, respectively. Fraction 3 (38 mg) was fractionated in 20% aqueous MeCN for 45 min, 20% to 100% in 5 min, followed by column cleaning. Olindenones B (**2**, 0.5 mg), C (**3**, 1.3 mg), D (**4**, 1.2 mg), and A (**1**, 4.2 mg) were collected from 13.2 to 15.0 min, 18.2 to 20.0 min, 23.5 to 26.0 min, and 44.5 to 46.8 min, respectively.

All isolated compounds were analyzed using one- and two-dimensional NMR and ESI-MS. The pure compounds were analyzed using the same HPLC conditions described above. In these conditions, the retention times (*t*_R_) of olindenones A–G (**1**–**7**) were 16.8, 8.6, 10.2, 10.8, 21.7, 21.9, and 21.2 min, respectively (Figure S9).

### Labeling Experiments and Labeled Compounds’ Analysis

For both labeling experiments, *S. olindensis* DAUFPE 5622 was cultured in SFd medium and extracted as described above. The first labeling experiment was performed with labeled glucose (glucose-1-^13^C). For this, 80% of labeled glucose replaced unlabeled glucose (3.2 g of glucose-1-^13^C, 0.8 g of unlabeled glucose, 20 g of soy flour per 1 L of deionized water). A 750 mL amount of SFd medium was produced with 2.4 g of glucose-1-^13^C, and after microbial cultivation labeled olindenones A (**1**) and B (**2**) were isolated as follows. The extract (∼300 mg) from a SFd medium culture (labeled glucose, 15 flasks, 750 mL) was prefractionated using an SPE cartridge (C18, 10 g, 60 mL) as described above. Fraction 3 (19 mg) was fractionated using isocratic elution with 30% aqueous MeCN for 35 min, followed by column cleaning. Subfractions 3-2 (2.6 mg) and 3-6 (1.8 mg) containing labeled olindenones B (**2**) and A (**1**) were collected from 5.5 to 11.1 min and 20.6 to 23.4 min, respectively. Fraction 3-2 (2.6 mg) was purified using an elution of 10% MeCN for 4 min, 10% to 20% MeCN in 6 min, 20% MeCN for 20 min, followed by column cleaning. ^13^C-enriched olindenone B (**2**, 1.1 mg) was collected from 21.3 to 23.1 min.

^13^C NMR spectra of labeled and unlabeled olindenone A were acquired (∼1.5 mg each, 20 h of NMR acquisition). The abundance of ^13^C in labeled olindenone A was determined by the formula ΔC = 1.1% × *L*/*U*, where 1.1% represents the natural abundance of ^13^C, *U* is the signal intensity in unlabeled compound, and *L* is the signal intensity in ^13^C-labeled compound (Table S8 and Figure S48).

The second labeling experiment was performed in a similar way. For this, 250 mg of sodium pyruvate-3-^13^C was used to prepare 500 mL of SFd microbial culture and produce the labeled olindenone A (**1**, ∼0.9 mg) (Table S8 and Figure S49).

#### Olindenone A (**1**):

white powder; [α]^25^_D_ +2.8 (*c* 0.18, MeOH); UV (MeCN/H_2_O) λ_max_ 241 nm; ^1^H NMR (CDCl_3_, 500 MHz) δ 5.71 (1H, br s, H-3), 3.54 (1H, d, *J* = 10.6 Hz, H-14a), 3.29 (1H, d, *J* = 10.6 Hz, H-14b), 2.60 (1H, dd, *J* = 17.0, 14.5 Hz, H-1β), 2.37 (1H, dt, *J* = 14.5, 3.3 Hz, H-10), 2.04 (1H, dd, *J* = 17.0, 3.3 Hz, H-1α), 1.88 (3H, br s, H-11), 1.67 (1H, m, H-6a), 1.57 (1H, m, H-7a), 1.56 (1H, m, H-6b), 1.54 (1H, m, H-9), 1.17 (1H, m, H-7b), 1.10 (3H, s, H-15), 1.09 (3H, s, H-12), 0.97 (3H, d, *J* = 7.4 Hz, H-13); ^13^C NMR (CDCl_3_, 125 MHz) d 200.6 (C, C-2), 171.5 (C, C-4), 125.9 (CH, C-3), 71.5 (CH_2_, C-14), 40.2 (CH, C-10), 40.0 (CH_2_, C-1), 39.9 (C, C-5), 39.2 (CH, C-9), 37.8 (C, C-8), 30.4 (CH_2_, C-6), 25.6 (CH_2_, C-7), 21.9 (CH_3_, C-15), 18.7 (CH_3_, C-11), 18.0 (CH_3_, C-12), 11.4 (CH_3_, C-13); HRESIMS *m*/*z* 259.1677 (calcd for C_15_H_24_O_2_Na^+^, 259.16685).

#### Olindenone B (**2**):

white powder; UV (MeCN/H_2_O) λ_max_ 241 nm; ^1^H NMR (CDCl_3_, 500 MHz) δ 6.08 (1H, br s, H-3), 4.40 (1H, dd, *J* = 17.3, 1.6 Hz, H-11a), 4.35 (1H, dd, *J* = 17.3, 1.6 Hz), 3.54 (1H, d, *J* = 10.7 Hz, H-14a), 3.29 (1H, d, *J* = 10.7 Hz, H-14b), 2.65 (1H, dd, *J* = 17.1, 14.5 Hz, H-1β), 2.43 (1H, ddd, *J* = 14.4, 4.2, 3.5 Hz, H-10), 2.10 (1H, dd, *J* = 17.1, 3.1 Hz, H-1α), 1.69–1.54 (2H, m, H-6a, H-7a), 1.25 (1H, m, H-6b), 1.54 (1H, m, H-9), 1.17 (1H, m, H-7b), 1.17 (3H, s, H-12), 1.11 (3H, s, H-15), 0.98 (3H, d, *J* = 7.7 Hz, H-13); ^13^C NMR (CDCl_3_, 125 MHz) δ 200.4 (C, C-2), 172.3 (C, C-4), 121.8 (CH, C-3), 71.3 (CH_2_, C-14), 60.4 (CH_2_, C-11), 40.3 (CH, C-10), 39.9 (CH_2_, C-1), 39.2 (C, C-5), 38.9 (CH, C-9), 37.5 (C, C-8), 29.5 (CH_2_, C-6), 25.3 (CH_2_, C-7), 21.8 (CH_3_, C-15), 19.0 (CH_3_, C-12), 11.1 (CH_3_, C-13); HRESIMS *m*/*z* 253.1791 (calcd for C_15_H_25_O_3_^+^, 253.17982).

#### Olindenone C (**3**):

white powder; UV (MeCN/H_2_O) λ_max_ 241 nm; ^1^H NMR (CD_3_OD, 500 MHz) δ 5.66 (1H, br s, H-3), 3.81 (1H, m, H-7), 3.80 (1H, d, *J* = 10.5 Hz, H-14a), 3.65 (1H, d, *J* = 10.5 Hz, H-14b), 2.78 (1H, dd, *J* = 17.2, 14.5 Hz, H-1β), 2.48 (1H, dt, *J* = 14.5, 4.0 Hz, H-10), 2.03 (1H, dd, *J* = 17.2, 4.0 Hz, H-1α), 1.95 (3H, br s, H-11), 2.00 (1H, dd, *J* = 14.2, 2.3 Hz, H-6a), 1.80 (1H, dd, *J* = 14.2, 3.1 Hz, H-6b), 1.60 (1H, dq, *J* = 7.3, 4.0 Hz, H-9), 1.42 (3H, s, H-12), 1.21 (3H, d, *J* = 7.3, H-13), 1.04 (3H, s, H-15); ^13^C NMR (CD_3_OD, 125 MHz) d 203.4 (C, C-2), 176.4 (C, C-4), 125.2 (CH, C-3), 74.2 (CH, C-7), 68.1 (CH_2_, C-14), 42.2 (C, C-8), 41.5 (CH, C-10), 41.4 (C, C-5), 40.4 (CH, C-9), 40.3 (CH_2_, C-1), 38.5 (CH_2_, C-6), 23.1 (CH_3_, C-15), 20.6 (CH_3_, C-12), 18.7 (CH_3_, C-11), 12.0 (CH_3_, C-13); HRESIMS *m*/*z* 253.1801 (calcd for C_15_H_25_O_3_^+^, 253.17982).

#### Olindenone D (**4**):

white powder; UV (MeCN/H_2_O) λ_max_ 241 nm; ^1^H NMR (CD_3_OD, 500 MHz) δ 5.78 (1H, br s, H-3), 3.70 (1H, d, *J* = 11.3 Hz, H-14a), 3.64 (1H, m, H-14b), 3.01 (1H, dt, *J* = 14.1, 3.9 Hz, H-10), 2.93 (1H, d, *J* = 12.9, H-6a), 2.73 (1H, dd, *J* = 17.0, 14.3 Hz, H-1β), 2.30 (1H, d, *J* = 12.9, H-6b), 2.22 (1H, m, H-1α), 2.07 (1H, m, H-9), 1.92 (3H, br s, H-11), 1.42 (3H, s, H-15), 1.09 (3H, s, H-12), 0.98 (3H, d, *J* = 7.7 Hz, H-13); ^13^C NMR (CD_3_OD, 125 MHz) δ 217.9 (C, C-7), 202.0 (C, C-2), 172.1 (C, C-4), 126.2 (CH, C-3), 64.8 (CH_2_, C-14), 54.0 (C, C-8), 47.8 (CH_2_, C-6), 45.7 (C, C-5), 44.2 (CH, C-9), 41.0 (CH, C-10), 39.5 (CH_2_, C-1), 22.4 (CH_3_, C-15), 19.5 (CH_3_, C-12), 18.3 (CH_3_, C-11), 11.6 (CH_3_, C-13); HRESIMS *m*/*z* 251.1652 (calcd for C_15_H_23_O_3_^+^, 251.16417).

#### Olindenone E (**5**):

white powder; UV (MeCN/H_2_O) λ_max_ 241 nm; ^1^H NMR (CDCl_3_, 500 MHz) δ 5.71 (1H, br s, H-3), 4.84 (1H, d, *J* = 3.4 Hz, H-1′), 4.02 (1H, m, H-3′), 3.91 (1H, q, *J* = 6.7 Hz, H-5′), 3.64 (1H, br s, H-4′), 3.52 (1H, d, *J* = 9.4 Hz, H-14a), 2.96 (1H, d, *J* = 9.4 Hz, H-14b), 2.59 (1H, dd, *J* = 16.9, 14.5 Hz, H-1β), 2.38 (1H, dt, *J* = 14.5, 2.7 Hz, H-10), 2.03 (1H, dd, *J* = 16.9, 2.7 Hz, H-1α), 1.94 (1H, dd, *J* = 12.5, 5.1 Hz, H-2′β), 1.88 (3H, br s, H-11), 1.77 (1H, td, *J* = 12.5, 3.4 Hz, H-2′α), 1.65 (1H, m, H-6a), 1.58 (1H, m, H-7a), 1.53 (1H, m, H-6b), 1.51 (1H, m, H-9), 1.28 (3H, d, *J* = 6.7 Hz, H-6′); 1.24 (1H, m, H-7b), 1.08 (3H, s, H-15), 1.08 (3H, s, H-12), 0.93 (3H, d, *J* = 7.5 Hz, H-13); ^13^C NMR (CDCl_3_, 125 MHz) δ 200.4 (C, C-2), 171.6 (C, C-4), 126.0 (CH, C-3), 99.2 (CH, C-1′), 77.0 (CH_2_, C-14), 71.4 (CH, C-4′), 66.2 (CH, C-5′), 66.0 (CH, C-3′), 40.2 (CH, C-10), 39.9 (CH_2_, C-1), 39.9 (C, C-5), 39.7 (CH, C-9), 37.1 (C, C-8), 33.2 (CH_2_, C-2′), 30.4 (CH_2_, C-6), 26.0 (CH_2_, C-7), 22.6 (CH_3_, C-15), 18.5 (CH_3_, C-11), 18.0 (CH_3_, C-12), 16.7 (CH_3_, C-6′), 11.4 (CH_3_, C-13); HRESIMS *m*/*z* 389.2289 (calcd for C_21_H_34_NaO_5_^+^, 389.22985).

#### Olindenone F (**6**):

white powder; UV (MeCN/H_2_O) λ_max_ 241 nm; ^1^H NMR (CD_3_OD, 500 MHz) δ 5.70 (1H, br s, H-3), 4.41 (1H, dd, *J* = 9.8, 1.5 Hz, H-1′), 3.68 (1H, dt, *J* = 12.4, 3.0, H-3′), 3.59 (1H, d, *J* = 9.1 Hz, H-14a), 3.50 (1H, q, *J* = 6.4 Hz, H-5′), 3.45 (1H, br s, H-4′), 3.28 (1H, d, *J* = 9.1 Hz, H-14b), 2.67 (1H, dd, *J* = 17.1, 14.7 Hz, H-1β), 2.38 (1H, dt, *J* = 14.7, 3.5 Hz, H-10), 1.99 (1H, dd, *J* = 17.1, 3.5 Hz, H-1α), 1.93 (3H, d, *J* = 1.1 Hz, H-11), 1.80 (1H, m, H-2′b), 1.70 (1H, m, H-2′a), 1.63 (1H, m, H-6a), 1.62 (1H, m, H-7a), 1.55 (1H, m, H-9), 1.30 (1H, m, H-6b), 1.27 (3H, d, *J* = 6.4 Hz, H-6′); 1.18 (1H, m, H-7b), 1.13 (3H, s, H-12), 1.12 (3H, s, H-15), 0.97 (3H, d, *J* = 7.7 Hz, H-13); ^13^C NMR (CD_3_OD, 125 MHz) δ 203.4 (C, C-2), 175.5 (C, C-4), 125.9 (CH, C-3), 101.9 (CH, C-1′), 78.3 (CH_2_, C-14), 71.7 (CH, C-5′), 71.2 (CH, C-4′), 69.8 (CH, C-3′), 41.3 (CH, C-10), 41.1 (C, C-5), 40.7 (CH, C-9), 40.4 (CH_2_, C-1), 37.5 (C, C-8), 35.1 (CH_2_, C-2′), 30.7 (CH_2_, C-6), 26.3 (CH_2_, C-7), 22.6 (CH_3_, C-15), 18.5 (CH_3_, C-11), 17.8 (CH_3_, C-12), 16.7 (CH_3_, C-6′), 11.2 (CH_3_, C-13); HRESIMS *m*/*z* 367.2465 (calcd for C_21_H_35_O_5_^+^, 367.24790).

#### Olindenone G (**7**):

white powder; UV (MeCN/H_2_O) λ_max_ 241 nm; ^1^H NMR (CD_3_OD, 500 MHz) δ 5.69 (1H, br s, H-3), 5.13 (1H, dd, *J* = 5.3, 1.5 Hz, H-1′), 4.24 (1H,dd, *J* = 6.9, 5.2, H-3′), 3.68 (1H, quint, *J* = 6.4 Hz, H-5′), 3.57 (1H, dd, *J* = 6.4, 5.2 Hz, H-4′), 3.71 (1H, d, *J* = 9.4 Hz, H-14a), 2.99 (1H, d, *J* = 9.4 Hz, H-14b), 2.67 (1H, dd, *J* = 16.9, 14.7 Hz, H-1β), 2.39 (1H, dt, *J* = 14.7, 3.5 Hz, H-10), 1.97 (1H, dd, *J* = 16.9, 3.5 Hz, H-1α), 2.19 (1H, ddd, *J* = 13.1, 5.2, 1.5 Hz, H-2′b), 1.93 (3H, d, *J* = 1.1 Hz, H-11), 2.02 (1H, ddd, *J* = 13.1, 6.9, 5.3 Hz, H-2′a), 1.69 (1H, m, H-6a), 1.65 (1H, m, H-7a), 1.60 (1H, m, H-6b), 1.49 (1H, m, H-9), 1.19 (3H, d, *J* = 6.4 Hz, H-6′); 1.21 (1H, m, H-7b), 1.13 (3H, s, H-12), 1.00 (3H, d, *J* = 7.7 Hz, H-13), 1.09 (3H, s, H-15); ^13^C NMR (CD_3_OD, 125 MHz) δ 176.0 (C, C-4), 125.9 (CH, C-3), 105.9 (CH, C-1′), 91.0 (CH, C-4′), 77.9 (CH_2_, C-14), 72.8 (CH, C-3′), 70.6 (CH, C-5′), 42.7 (CH_2_, C-2′), 41.5 (CH, C-10), 41.1 (C, C-5), 40.8 (CH, C-9), 40.7 (CH_2_, C-1), 37.9 (C, C-8), 31.1 (CH_2_, C-6), 26.6 (CH_2_, C-7), 22.6 (CH_3_, C-15), 19.0 (CH_3_, C-6′), 18.5 (CH_3_, C-11), 17.9 (CH_3_, C-12), 11.2 (CH_3_, C-13), n.o. (C, C-2); HRESIMS *m*/*z* 389.2310 (calcd for C_21_H_34_NaO_5_^+^, 389.22985).

## Supplementary Material



## Data Availability

The raw NMR data for olindenones A–G (**1**–**7**) have been deposited in nmrXiv (https://nmrxiv.org/) and can be found at 10.57992/NMRXIV.P20.

## Notes

NMR and MS data and genomic analysis (PDF)
