# Supplementary material for: Unusual Sesquiterpenes from Streptomyces olindensis DAUFPE 5622
Source: J Nat Prod. 2024 Feb 29;87(3):491–500. doi: 10.1021/acs.jnatprod.3c00752 (PMC12453296; doi:10.1021/acs.jnatprod.3c00752)
Supplement: Supplementary file 1 [file np3c00752_si_001.pdf]

Supporting Information

Unusual sesquiterpenes from *Streptomyces olindensis* DAUFPE 5622

Fernanda O. Chagas[a,b,*], Leandro M. Garrido[c], Raphael Conti[a], Ricardo M. Borges[b], Vincent A. Bielinski[d,e], Gabriel Padilla[c], Mônica T. Pupo[a,*]

[a]Faculdade de Ciências Farmacêuticas de Ribeirão Preto, Universidade de São Paulo, Ribeirão Preto, SP, Brazil
[b]Instituto de Pesquisas de Produtos Naturais Walter Mors, Universidade Federal do Rio de Janeiro, Rio de Janeiro, RJ, Brazil
[c]Instituto de Ciências Biomédicas, Universidade de São Paulo, São Paulo, SP, Brazil
[d]Synthetic Biology and Bioenergy Group, J. Craig Venter Institute, La Jolla, CA, USA
[e]Instituto de Química, Universidade Federal do Rio de Janeiro, Rio de Janeiro, RJ, Brazil

*Corresponding authors: mtpupo@fcfrp.usp.br (MTP) and ferchagas@ippn.ufrj.br (FOC)

List of contents

Table S1. NMR data of compound **1** …………………………………………………..………… 1

Figure S1. ^{1}H NMR spectrum of compound **1** ……………………………………………………….. 1

Figure S2. ^{13}C NMR spectrum of compound **1**……………………………………………………….. 2

Figure S3. DEPT spectrum of compound **1**…………………………………………………………... 2

Figure S4. *g*HSQC spectrum of compound **1**………………………………………………………… 3

Figure S5. *g*HMBC spectrum of compound **1**………………………………………………………… 3

Figure S6. *g*COSY spectrum of compound **1**………………………………………..…………….. 4

Figure S7. NOEDIFF spectra of compound **1**……………………………………….…………… 4

Figure S8. HR-ESI-MS spectrum of compound **1** ……………………………………..………. 5

Figure S9. Chromatogram of a crude extracted from *S. olindensis* DAUFPE 5622 ..…………… 5

Table S2. NMR data of compound **2** ……………………………………………………………..… 6

Figure S10. ^{1}H NMR spectrum of compound **2**………………………………………………………. 6

Figure S11. *g*HSQC spectrum of compound **2**……………………………………………….……… 7

Figure S12. *g*HMBC spectrum of compound **2**………………………………………………………….. 7

Figure S13. *g*COSY spectrum of compound **2** ………………………………………………………. 8

Figure S14. NOESY spectrum of compound **2**…………………………………………………………. 8

Figure S15. HR-ESI-MS spectrum of compound **2**………………………………………………… 9

Table S3. NMR data of compound **3** ……………………………………………………….……… 9

Figure S16. ^{1}H NMR spectrum of compound **3**………………………………………………….... 10

Figure S17. *g*HSQC spectrum of compound **3**…………………………………………….………. 10

Figure S18. *g*HMBC spectrum of compound **3**………………………………………………………. 11

Figure S19. *g*COSY spectrum of compound **3**……………………………………………………….. 11

Figure S20. HR-ESI-MS spectrum of compound **3**………………………………………………… 12

Table S4. NMR data of compound **4** ………………………………………………………..……… 12

Figure S21. ^{1}H NMR spectrum of compound **4** ………………………………………………… 13

Figure S22. *g*HSQC spectrum of compound **4**………………………………………………….…. 13

Figure S23. *g*HMBC spectrum of compound **4**……………………………………………………. 14

Figure S24. *g*COSY spectrum of compound **4**……………………………………………………… 14

Figure S25. HR-ESI-MS spectrum of compound **4** ……………………………………….……… 15

Table S5. NMR data of compound **5** …………………………………………………………..… 15

Figure S26. ^{1}H NMR spectrum of compound **5**………………………………………………………. 16

Figure S27. ^{1}H NMR spectrum of compound **5**………………………………………………………. 16

Figure S28. *g*HSQC spectrum of compound **5**……………………………………………………. 17

Figure S29. *g*HMQC spectrum of compound **5**………………………………………………………. 17

Figure S30. *g*COSY spectrum of compound **5** ………………………………………………………………… **18**
Figure S31. NOESY spectrum of compound **5**……………………………………………………………. **18**
Figure S32. HR-ESI-MS spectrum of compound **5**………………………………………………….. **19**
Table S6. NMR data of compound **6** ……………………………………………………………………..… **19**
Figure S33. ^{1}H NMR spectrum of compound **6**…………………………………………………………. **20**
Figure S34. *g*HSQC spectrum of compound **6** ……………………………………………………...…… **20**
Figure S35. *g*HMBC spectrum of compound **6**…………………………………………………………. **21**
Figure S36. *g*COSY spectrum of compound **6**………………………………………………………. **21**
Figure S37. *g*TOCSY spectrum of compound **6**……………………………………………………... **22**
Figure S38. NOESY spectrum of compound **6**…………………………………………………………. **22**
Figure S39. HR-ESI-MS spectrum of compound **6**……………………………………………… **23**
Table S7. NMR data of compound **7** ……………………………………………………………………..… **23**
Figure S40. ^{1}H NMR spectrum of compound **7**…………………………………………………………. **24**
Figure S41. *g*HSQC spectrum of compound **7**……………………………………………………….. **24**
Figure S42. *g*HMBC spectrum of compound **7**…………………………………………………………. **25**
Figure S43. *g*COSY spectrum of compound **7**………………………………………………………… **25**
Figure S44. *g*TOCSY spectrum of compound **7**…………………………………………………..…. **26**
Figure S45. NOESY spectrum of compound **7**…………………………………………………………. **26**
Figure S46. HR-ESI-MS spectrum of compound **7**……………………………………………….…. **27**
Figure S47. Possible steps in the biosynthesis of the sugars found in olindenones E-G (**5-7**).… **27**
Table S8. ^{13}C NMR of ^{13}C-enriched **1** from *S. olindensis* DAUFPE 5622 cultivation with glucose-1-^{13}C or pyruvate-3-^{13}C …………………………………………………………………..…... **28**
Figure S48. ^{13}C NMR spectra of compound **1** unlabeled and labeled using glucose-1-^{13}C ……... **29**
Figure S49. ^{13}C NMR spectra of compound **1** unlabeled and labeled using pyruvate-3-^{13}C …….. **30**
Table S9. Terpene putative BGCs identified by antiSMASH ………………………………………. **31**
Figure S50. SSN using the putative TC KDN80181.1 from *S. olindensis* DAUFPE 5622……… **31**

Table S1. NMR data of compound **1**

Position	δ ^{13}C [c]	δ ^{13}C and DEPT [d]	δ ^{1}H (mult., *J* Hz, integral) [d]	*g*COSY [d]	*g*HMBC [d]	NOEDIFF [d]
**1**	41.3	40.0 - CH_2	2.60 (dd, 17.0, 14.5, 1H) - β<br>2.04 (dd, 17.0, 3.3, 1H) - α	H-1α, H-10<br>H-1β, H-10	C-2, C-5, C-10	-
**2**	203.3	200.6 - C	-	-	-	-
**3**	126.1	125.9 - CH	5.71 (br s, 1H)	H-11[e]	C-5, C-11	-
**4**	175.6	171.5 - C	-	-	-	-
**5**	40.8	39.9 - C	-	-	-	-
**6**	31.4	30.4 - CH_2	1.67 (m, 1H) - a<br>1.56 (m, 1H) - b	H-6b, H-7a, H-7b<br>H-6a, H-7a, H-7b	C-4, C-5, C-7,<br>C-8, C-12	-
**7**	26.6	25.6 - CH_2	1.57 (m, 1H) - a<br>1.17 (m, 1H) - b	H-6a, H-6b, H-7b<br>H-6a, H-6b, H-7a	C-5, C-6, C-15	-
**8**	38.6	37.8 - C	-	-	-	-
**9**	40.6	39.2 - CH	1.54 (m, 1H)	H-10, H-13	C-1, C-7, C-13,<br>C-14, C-15	-
**10**	41.7	40.2 - CH	2.37 (dt, 14.5, 3.3, 1H)	H-1α, H-1β, H-9	C-1, C-5	H-15
**11**	18.8	18.7 - CH_3	1.88 (br s, 3H)	H-3[e]	C-3, C-4, C-5	-
**12**	18.2	18.0 - CH_3	1.09 (s, 3H)	-	C-4, C-5, C-6	-
**13**	11.5	11.4 - CH_3	0.97 (d, 7.4, 3H)	H-9	C-8, C-9	H-1β, H-14a,<br>H-14b
**14**	71.4	71.5 - CH_2	3.54 (d, 10.6, 1H) - a<br>3.29 (d, 10.6, 1H) - b	H-14b<br>H-14a	C-7, C-8, C-9,<br>C-15	-
**15**	22.3	21.9 - CH_3	1.10 (s, 3H)	-	C-7, C-8, C-9,<br>C-14	-

Acquired at 500 MHz in CD_3OD [c] or $CDCl_3$ [d]. α and β refer to planar orientation while a and b distinguish each geminal hydrogen.
[e] long range coupling (^4J) in *g*COSY. NOEDIFF experiments were only performed for H-3, H-10 and H-13.

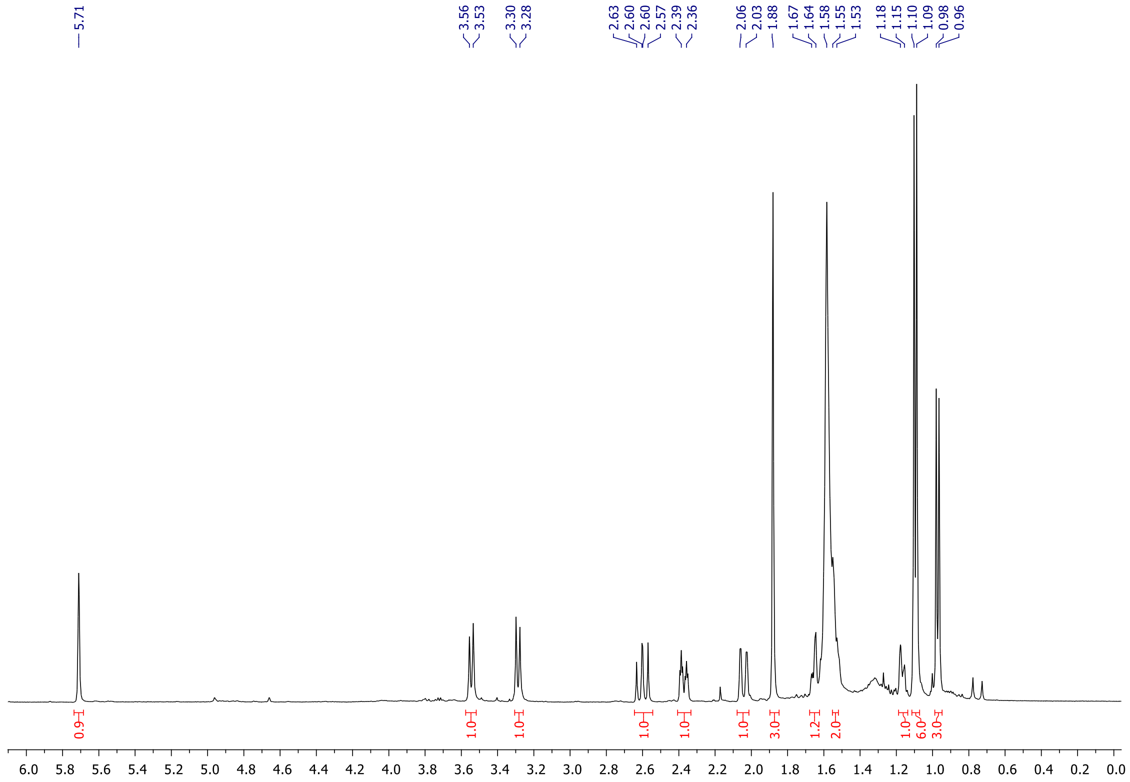

Figure S1. ^{1}H NMR spectrum (500 MHz, $CDCl_3$) of compound **1**.

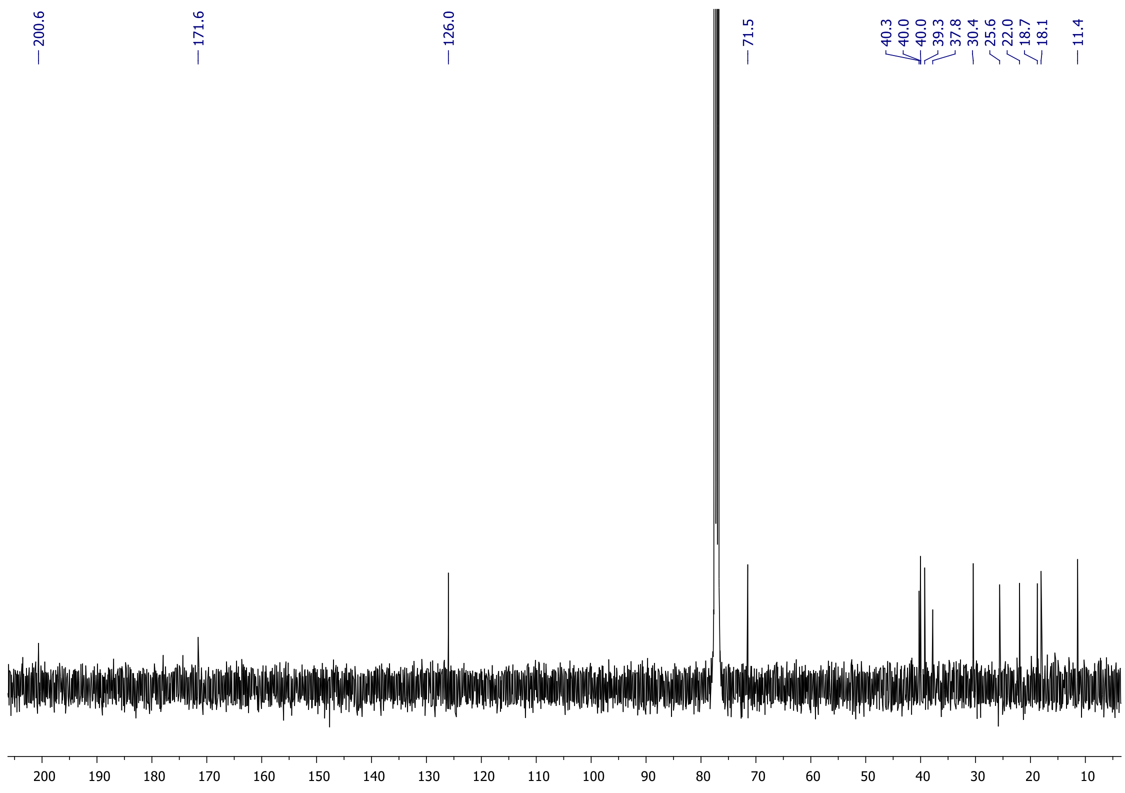

Figure S2. ^{13}C NMR spectrum (125 MHz, $CDCl_3$) of compound **1**.

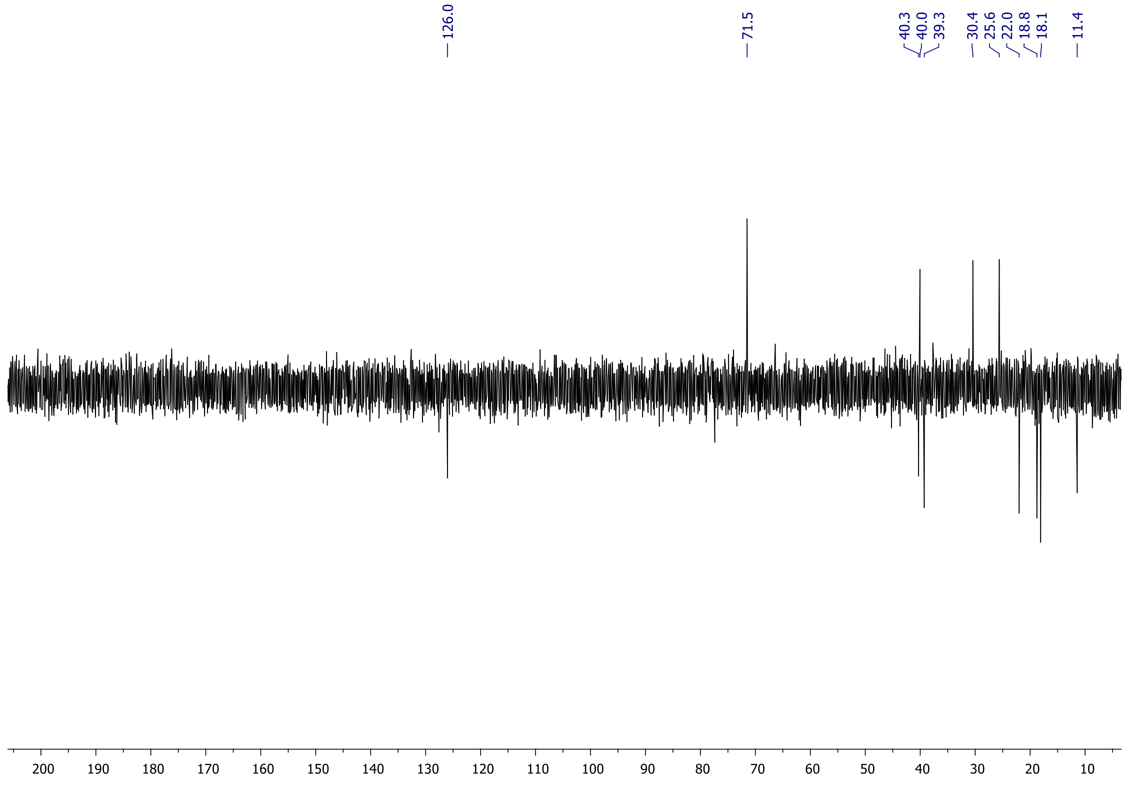

Figure S3. DEPT spectrum (125 MHz, $CDCl_3$) of compound **1**.

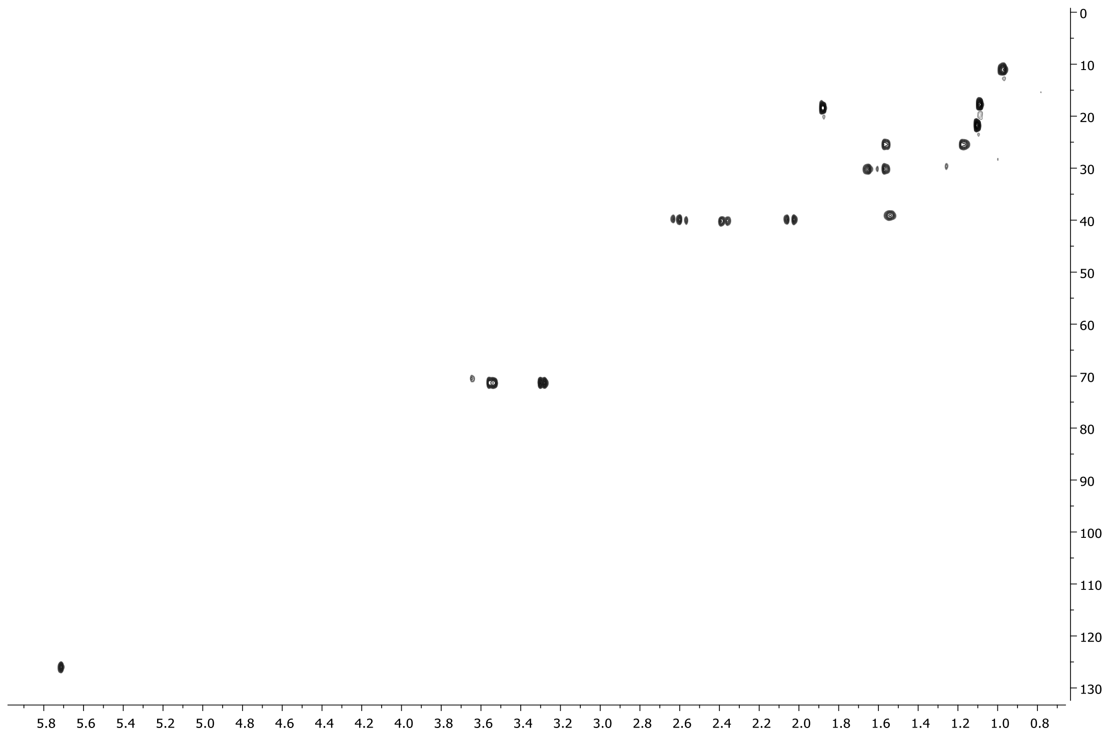

Figure S4. *g*HSQC spectrum (500 MHz, $CDCl_3$) of compound **1**.

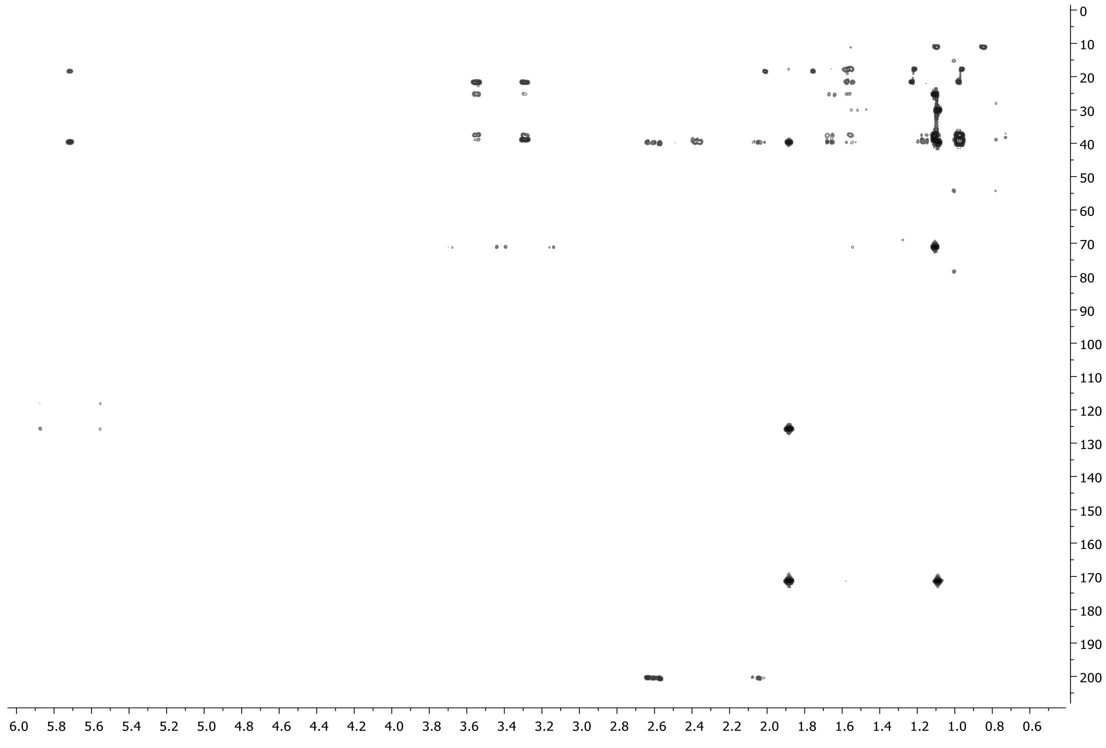

Figure S5. *g*HMBC spectrum (500 MHz, $CDCl_3$) of compound **1**.

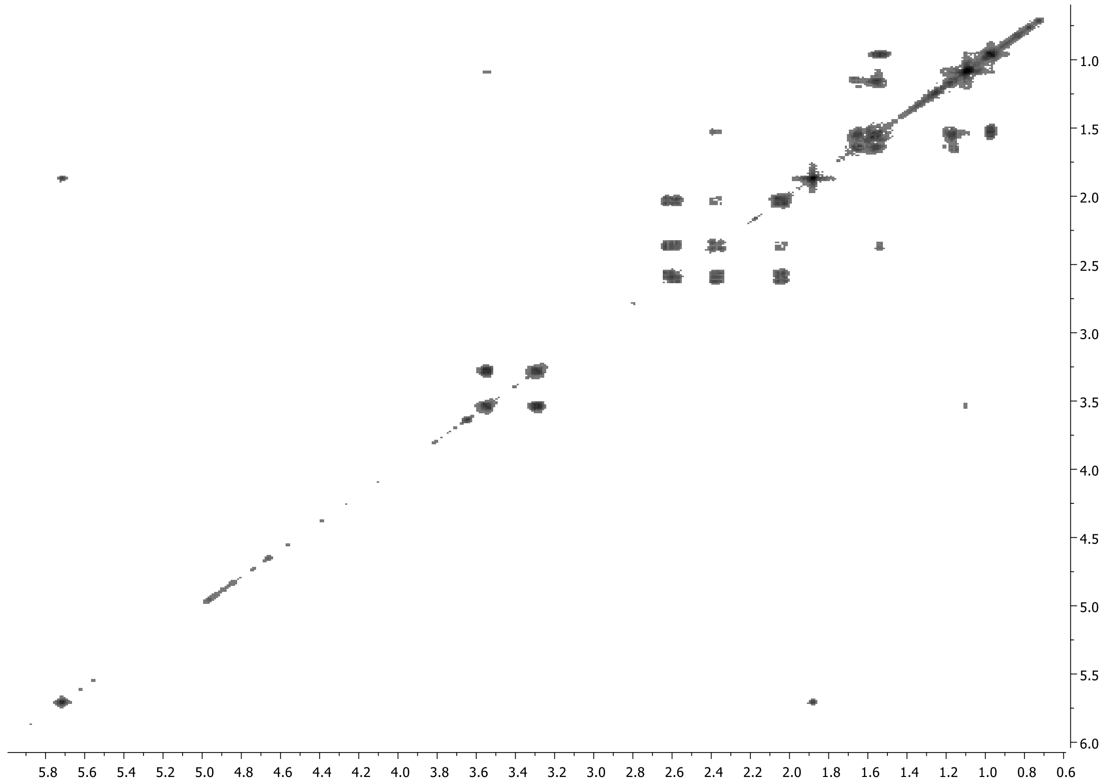

Figure S6. *g*COSY spectrum (500 MHz, $CDCl_3$) of compound **1**.

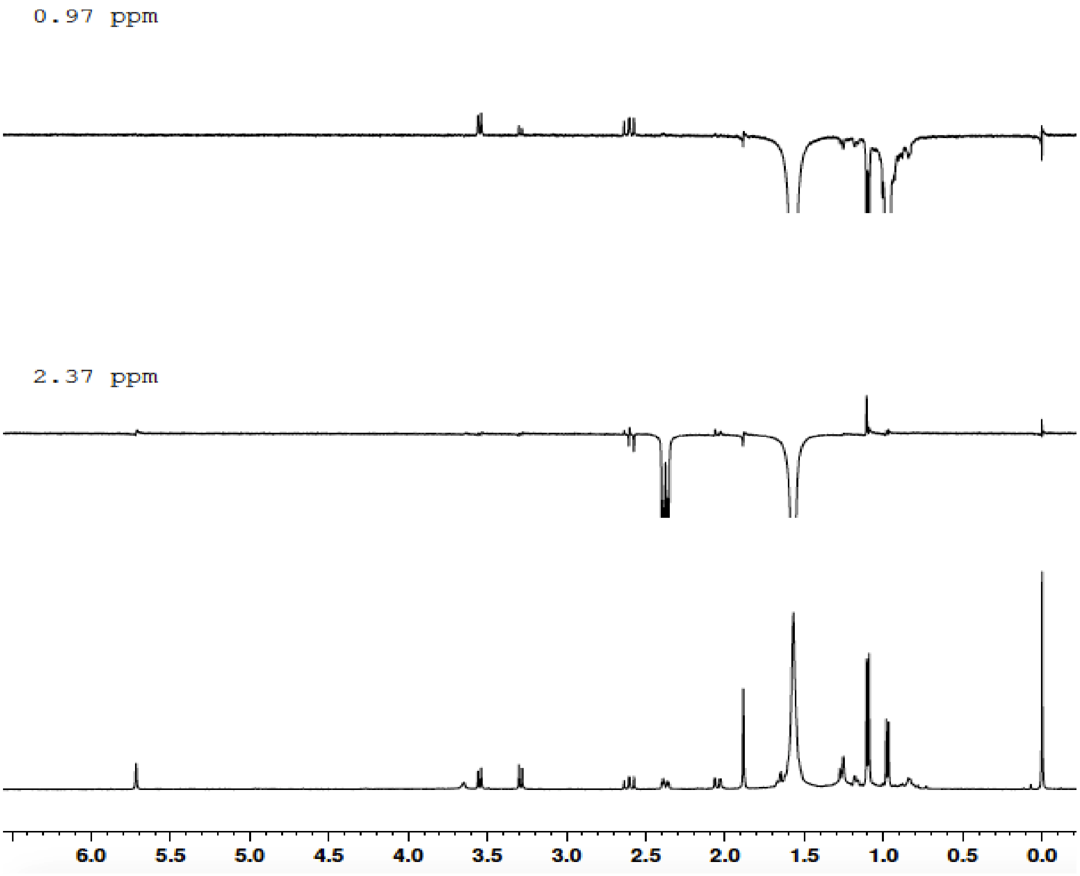

Figure S7. NOEDIFF spectra (500 MHz, $CDCl_3$) of compound **1**.

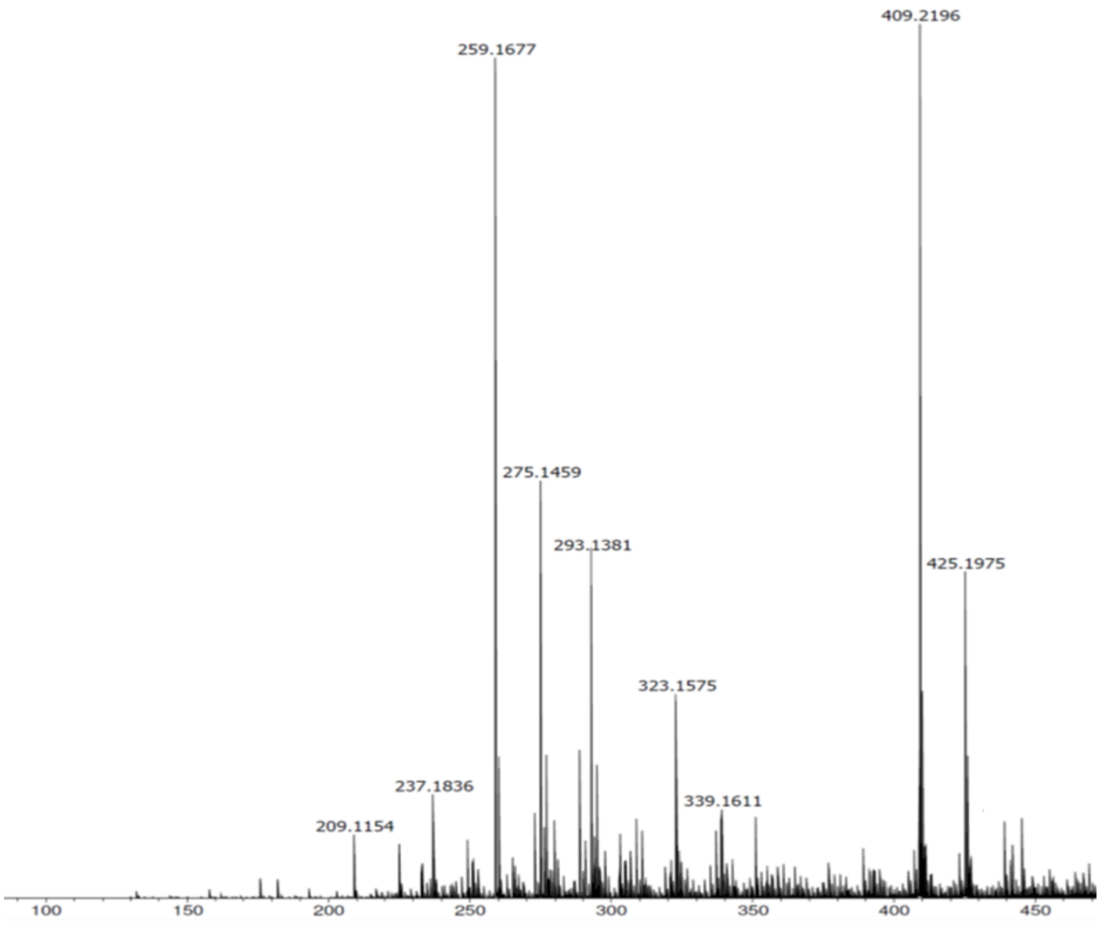

Figure S8. HR-ESI-MS spectrum of compound **1** (*m/z* 237.1836 $[M+H]^+$, calc.$C_{15}H_{25}O_2^+$ 237.18491, err. 5.5 ppm; and *m/z* 259.1677 $[M+Na]^+$, calc. $C_{15}H_{24}O_2Na^+$ 259.16685, err. 3.3 ppm).

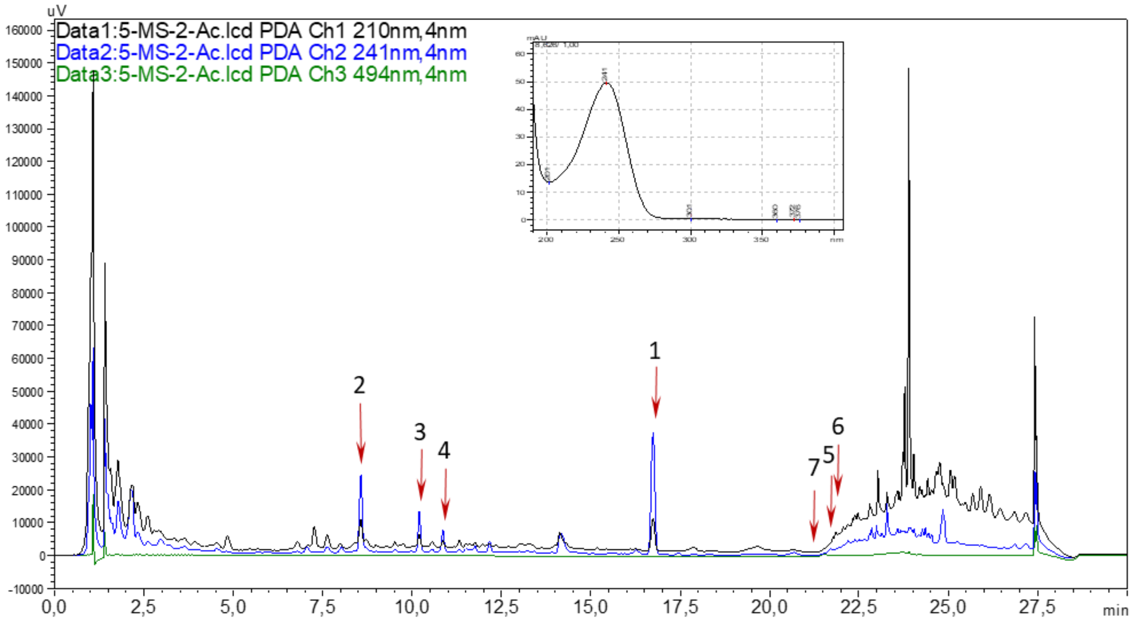

Figure S9. Chromatogram of a crude extracted from an SFM culture of *S. olindensis* DAUFPE 5622. Olindenones A-G (**1-7**) and the UV profile of these compounds with λ_{max} at 241 nm.

Table S2. NMR data of compound **2**

Position	δ ^{13}C [c]	δ ^{1}H (mult., *J* Hz, integral)	*g*COSY	*g*HMBC	NOESY
**1**	39.9	2.65 (dd, 17.1, 14.5, 1H) - β<br>2.10 (dd, 17.1, 3.1, 1H) - α	H-1α, H-10<br>H-1β, H-10	C-2	H-1α, H-10, H-12, H-13<br>H-1β
**2**	200.4	-	-	-	-
**3**	121.8	6.08 (br s, 1H)	H-11a, H-11b	C-11[d]	-
**4**	172.3	-	-	-	-
**5**	39.2	-	-	-	-
**6**	29.5	1.54-1.69* (m, 1H) - a<br>1.25* (m, 1H) - b	overlapped	-	-
**7**	25.3	1.54-1.69* (m, 1H) - a<br>1.17* (m, 1H) - b	overlapped	-	-
**8**	37.5	-	-	-	-
**9**	38.9	1.54 (m, 1H)	H-10, H-13	-	-
**10**	40.3	2.43 (ddd, 14.4, 4.2, 3.5, 1H)	H-1α, H-1β, H-9	C-2	H-1β, H-13
**11**	60.4	4.40 (dd, 17.3, 1.6, 1H) - a<br>4.35 (dd, 17.3, 1.6, 1H) - b	H-3<br>H-3	C-3[d], C-4	H-12<br>H-12
**12**	19.0	1.17 (s, 3H)	-	C-4, C-6, C-10	H-1β
**13**	11.1	0.98 (d, 7.7, 3H)	H-9	C-8, C-9, C-10	H-1β, H-14a
**14**	71.3	3.54 (d, 10.7, 1H) - a<br>3.29 (d, 10.7, 1H) - b	H-14b<br>H-14a	C-9, C-15	H-13, H-14b<br>H-14a, H-15
**15**	21.8	1.11 (s, 3H)	-	C-7, C-8, C-9, C-14	H-10, H-14a

Acquired at 500 MHz in $CDCl_3$. α and β refer to planar orientation; a and b distinguish each geminal hydrogen. [c] indirectly determined by *g*HSQC and *g*HMBC. [d] long range coupling (^{4}J) in *g*COSY. *uncertain, assigned from *g*HSQC spectrum. This compound was obtained from a culture supplemented with glucose-1-^{13}C and is ^{13}C-enriched.

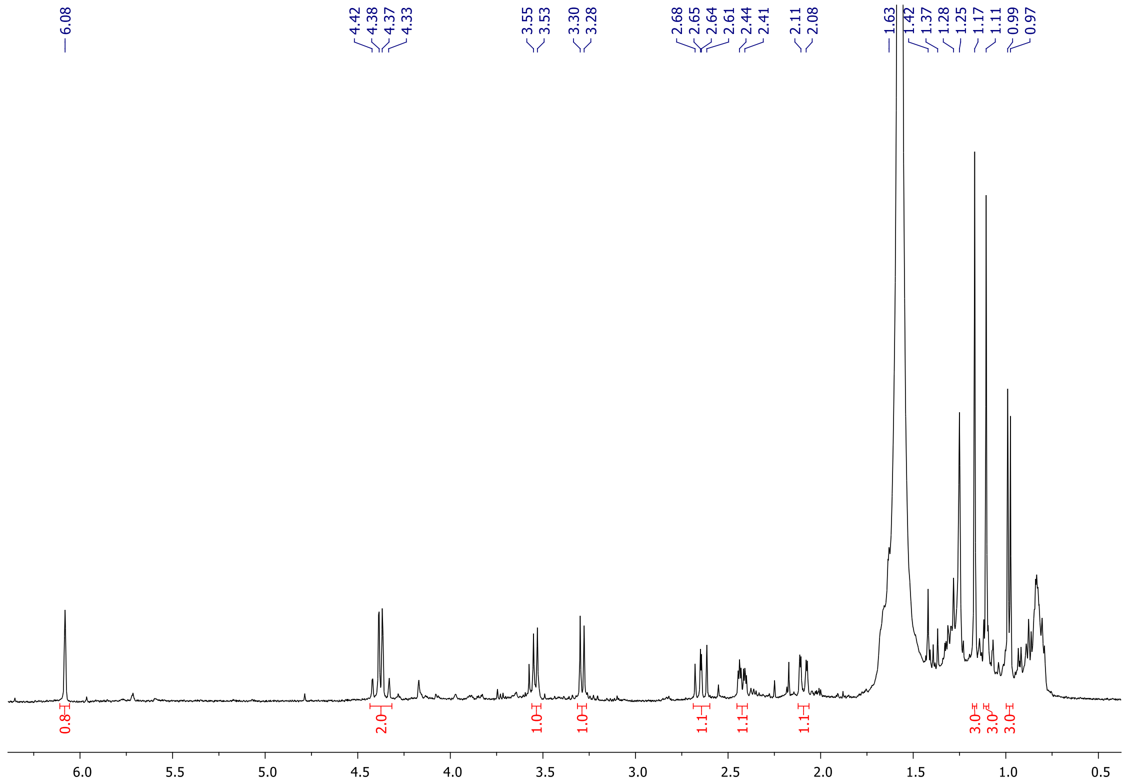

Figure S10. ^{1}H NMR spectrum (500 MHz, $CDCl_3$) of compound **2**.

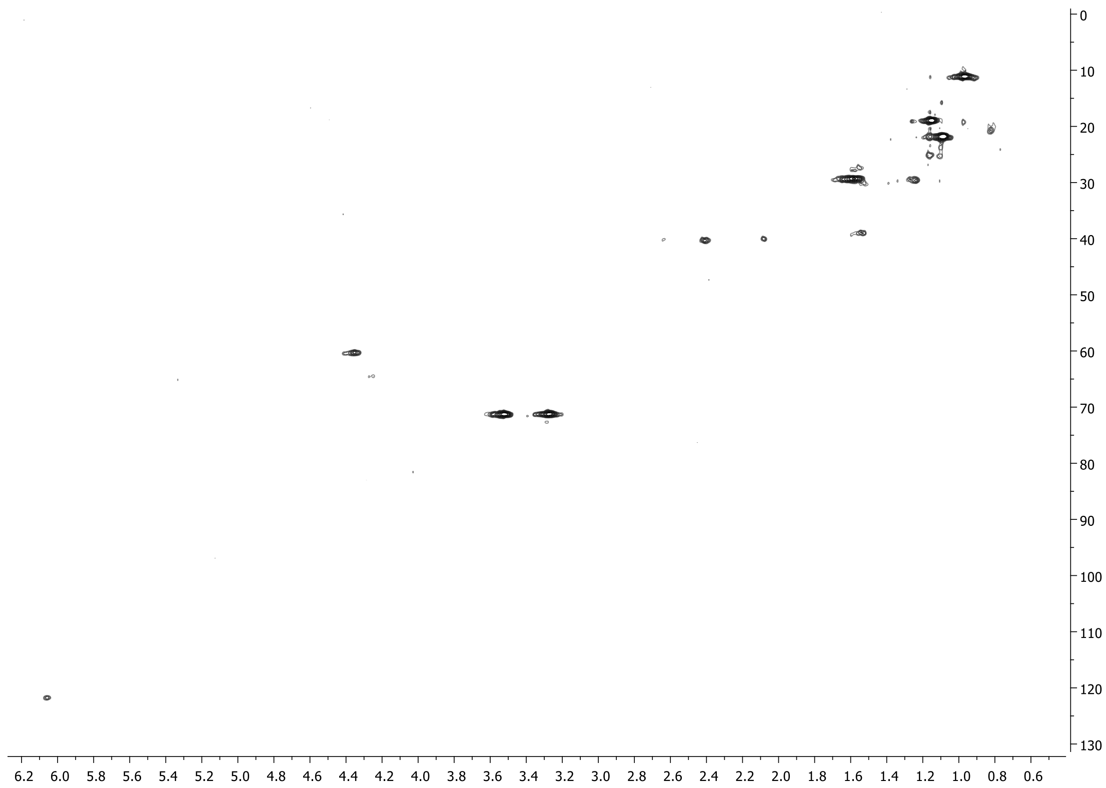

Figure S11. *g*HSQC spectrum (500 MHz, $CDCl_3$) of compound **2**.

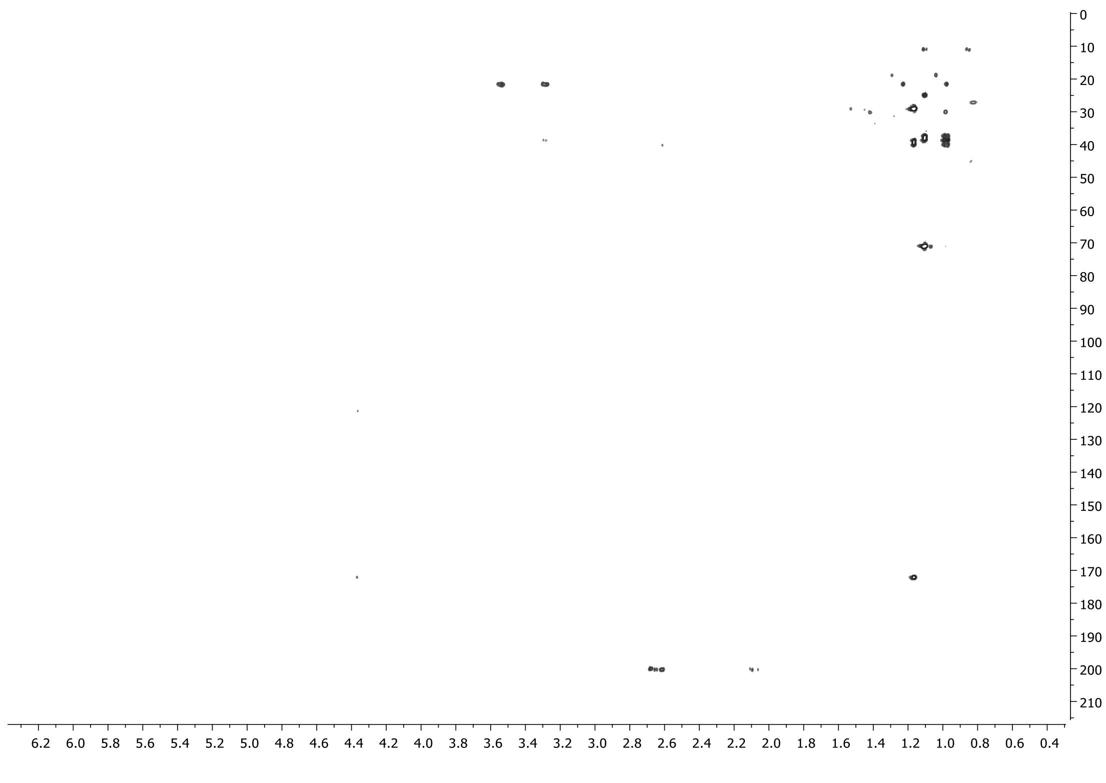

Figure S12. *g*HMBC spectrum (500 MHz, $CDCl_3$) of compound **2**.

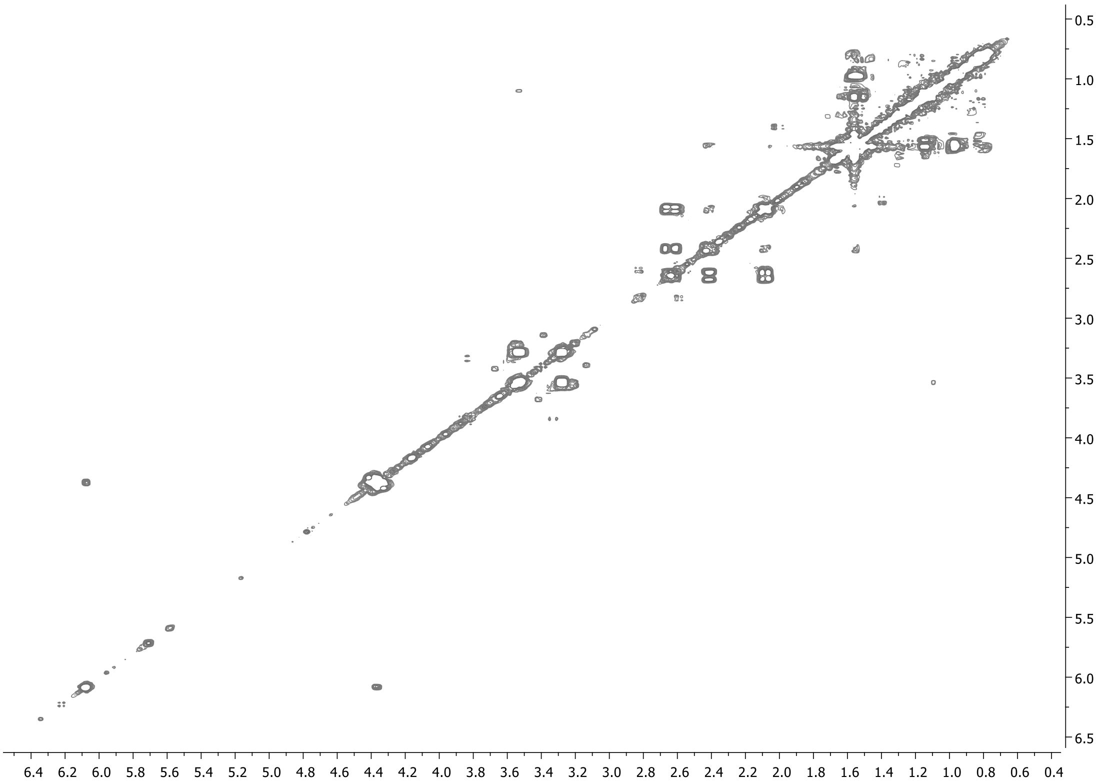

Figure S13. *g*COSY spectrum (500 MHz $CDCl_3$) of compound **2**.

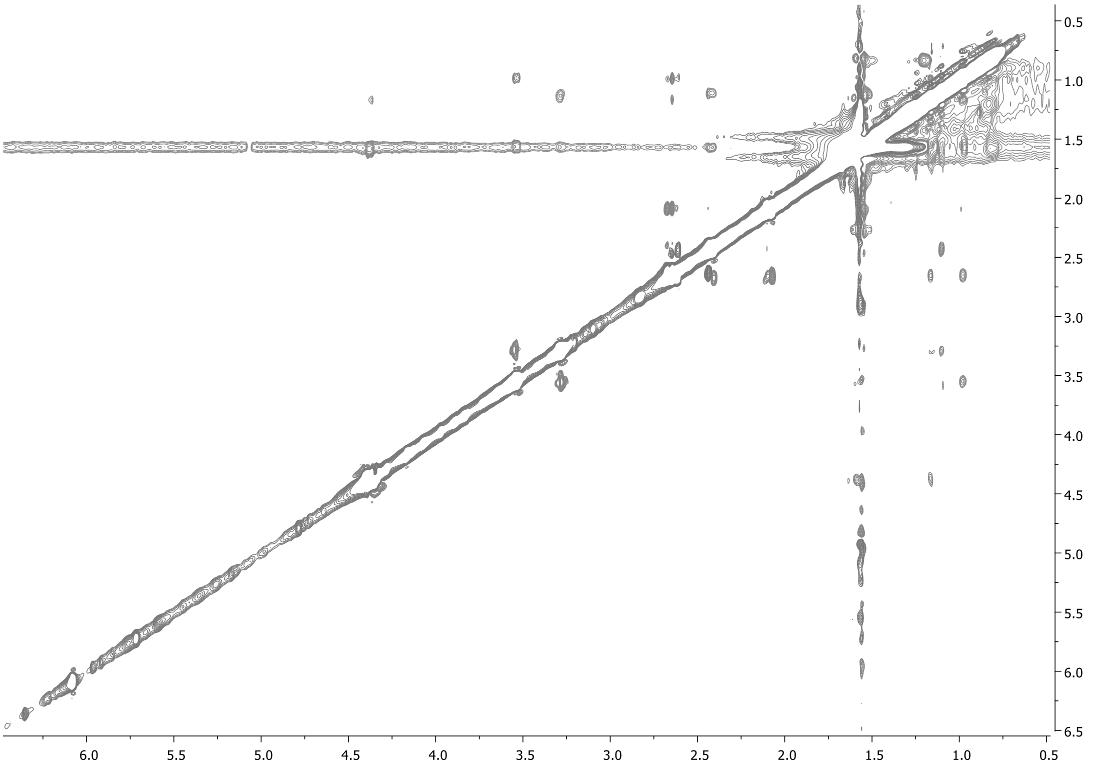

Figure S14. NOESY spectrum (500 MHz, $CDCl_3$) of compound **2**.

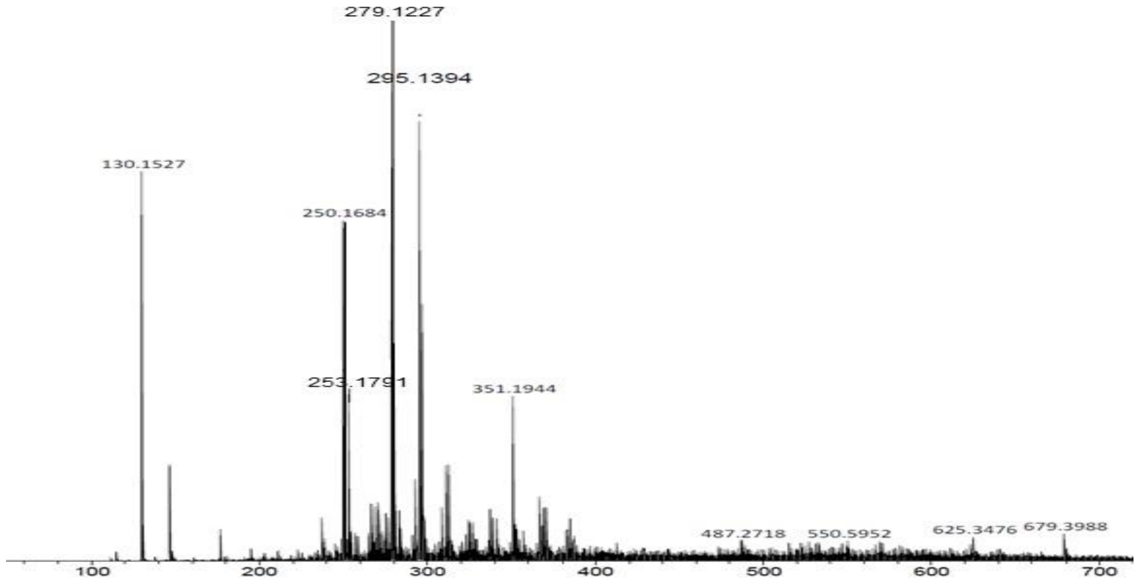

Figure S15. HR-ESI-MS spectrum of compound **2** (*m/z* 253.1791 $[M+H]^+$, calc.$C_{15}H_{25}O_3^+$ 253.17982, err. 2.8 ppm).

Table S3. NMR data of compound **3**

Position	δ ^{13}C [c]	δ ^{1}H (mult., *J* Hz, integral)	*g*COSY	*g*HMBC
**1**	40.3	2.78 (dd, 17.2, 14.5, 1H) - β<br>2.03 (dd, 17.2, 4.0, 1H) - α	H-1α, H-10<br>H-1β	C-2, C-10
**2**	203.4	-	-	-
**3**	125.2	5.66 (br s, 1H)	H-11[d]	-
**4**	176.4	-	-	-
**5**	41.4	-	-	-
**6**	38.5	2.00 (dd, 14.2, 2.3, 1H) - a<br>1.80 (dd, 14.2, 3.1, 1H) - b	H-6b, H-7<br>H-6a, H-7	C-3[e], C-4, C-5
**7**	74.2	3.81 (m, 1H)	H-6a, H-6b	C-8, C-15
**8**	42.2	-	-	-
**9**	40.4	1.60 (dq, 4.0, 7.3, 1H)	H-13	-
**10**	41.5	2.48 (dt, 14.5, 4.0, 1H)	H-1β	C-9
**11**	18.7	1.95 (br s, 3H)	H-3[d]	C-3, C-4, C-5
**12**	20.6	1.42 (s, 3H)	-	C-4, C-5, C6
**13**	12.0	1.21 (d, 7.3, 3H)	H-9	C-8, C-9
**14**	68.1	3.80 (d, 10.5*, 1H) - a<br>3.67 (d, 10.5*, 1H) - b	H-14b<br>H-14a	C-7, C-8, C-9, C-15
**15**	23.1	1.04 (s, 3H)	-	C-7, C-8, C-9, C-14

Acquired at 500 MHz in CD_3OD. α and β refer to planar orientation; a and b are used to distinguish each geminal hydrogen. [c] indirectly determined by *g*HSQC and *g*HMBC. [d] long range coupling (^4J) in *g*COSY. [e] ^4J coupling in *g*HMBC. *imprecise *J* measurement

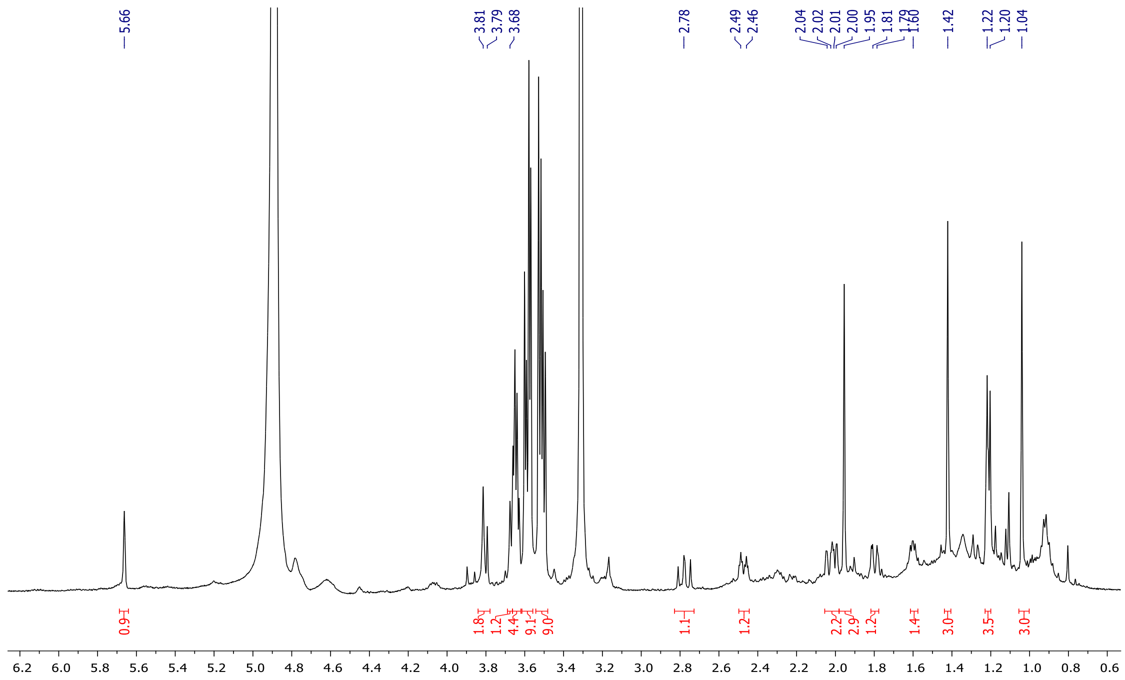

Figure S16. ^{1}H NMR spectrum (500 MHz, CD_3OD) of compound **3**. Signals corresponding to this compound are assigned.

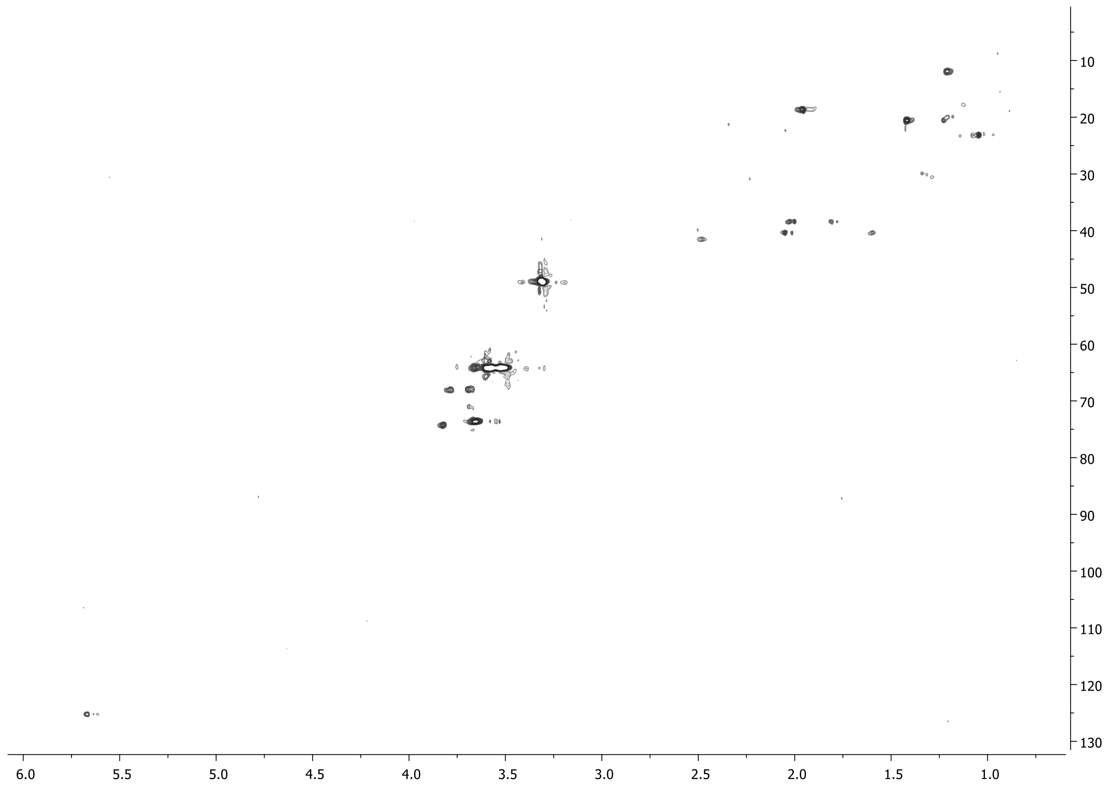

Figure S17. *g*HSQC spectrum (500 MHz, CD_3OD) of compound **3**.

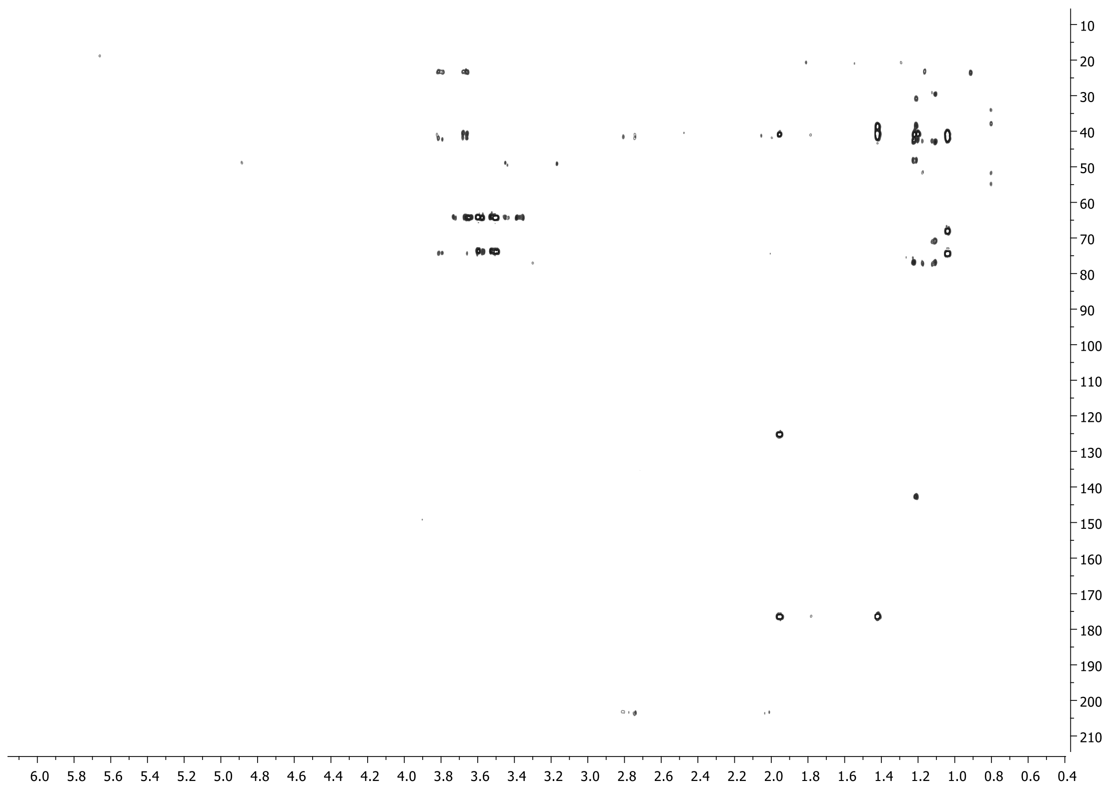

Figure S18. *g*HMBC spectrum (500 MHz, CD_3OD) of compound **3**.

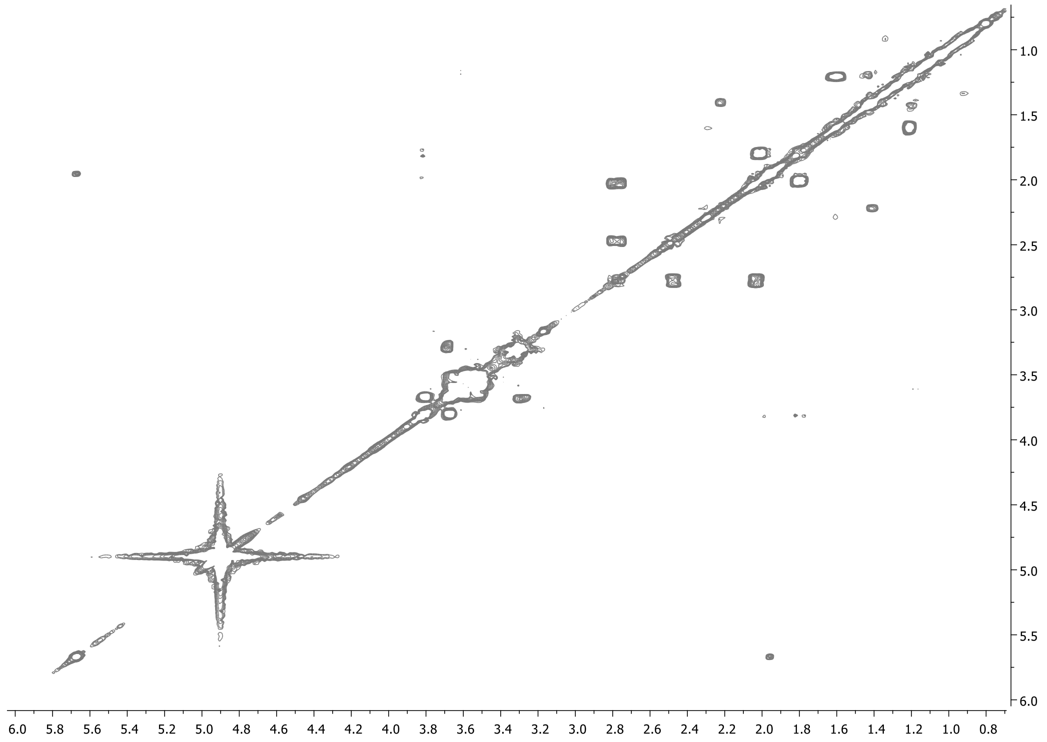

Figure S19. *g*COSY spectrum (500 MHz, CD_3OD) of compound **3**.

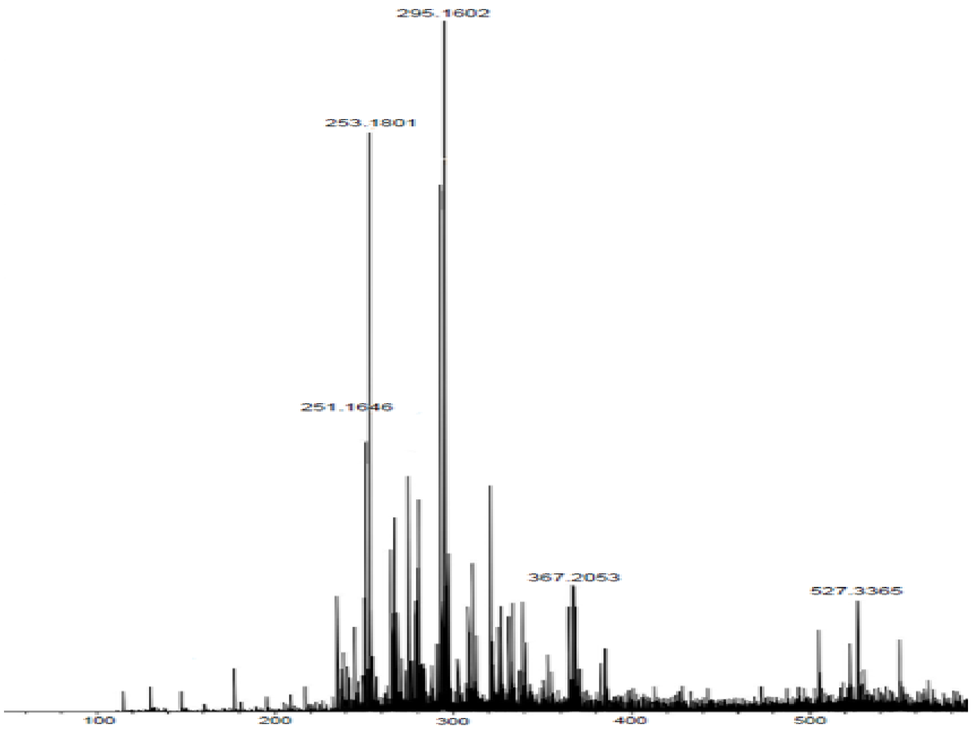

Figure S20. HR-ESI-MS spectrum of compound **3** (*m/z* 253.1801 $[M+H]^+$, calc.$C_{15}H_{25}O_3^+$ 253.17982, err. 1.1 ppm).

Table S4. NMR data of compound **4**

Position	δ ^{13}C [c]	δ ^{1}H (mult., *J* Hz, integral)	*g*COSY	*g*HMBC
**1**	39.5	2.73 (dd, 17.0, 14.3, 1H) - β<br>2.22 (m, 1H) - α	H-1α, H-10<br>H-1β	C-2, C-10
**2**	202.0	-	-	-
**3**	126.2	5.78 (br s, 1H)	H-11[d]	-
**4**	172.1	-	-	-
**5**	45.7	-	-	-
**6**	47.8	2.93 (d, 12.9, 1H) - a<br>2.30 (d, 12.9, 1H) - b	H-6b<br>H-6a	C-4, C-5, C-7, C-8, C-10, C12
**7**	217.9	-	-	-
**8**	54.0	-	-	-
**9**	44.2	2.07 (m, 1H)	H-13	-
**10**	41.0	3.01 (dt, 14.1, 3.9, 1H)	H-9, H-1β	-
**11**	18.3	1.92 (br s, 3H)	H-3[d]	C-3, C-4, C-5
**12**	19.5	1.09 (s, 3H)	-	C-4, C-5, C-6, C-10
**13**	11.6	0.98 (d, 7.7, 3H)	H-9	C-8, C-9, C-10
**14**	64.8	3.70 (d, 11.3, 1H) - a<br>3.64 - b overlapped	-<br>-	C-8, C-9, C-15
**15**	22.4	1.42 (s, 3H)	-	C-7, C-8, C-9, C-14

Acquired at 500 MHz in CD_3OD. α and β refer to planar orientation; a and b are used to distinguish each geminal hydrogen. [c] indirectly determined by *g*HSQC and *g*HMBC. [d] long range coupling (^4J) in *g*COSY.

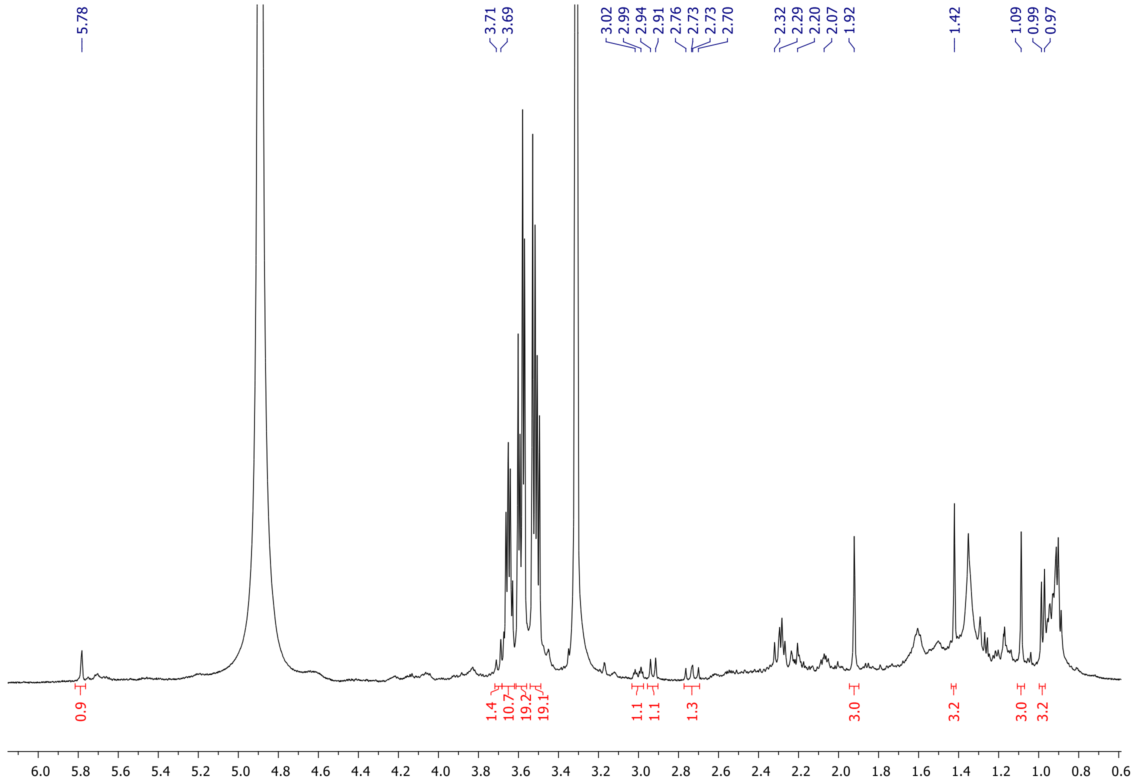

Figure S21. ^{1}H NMR spectrum (500 MHz, CD_3OD) of compound **4**. Signals corresponding to this compound are assigned.

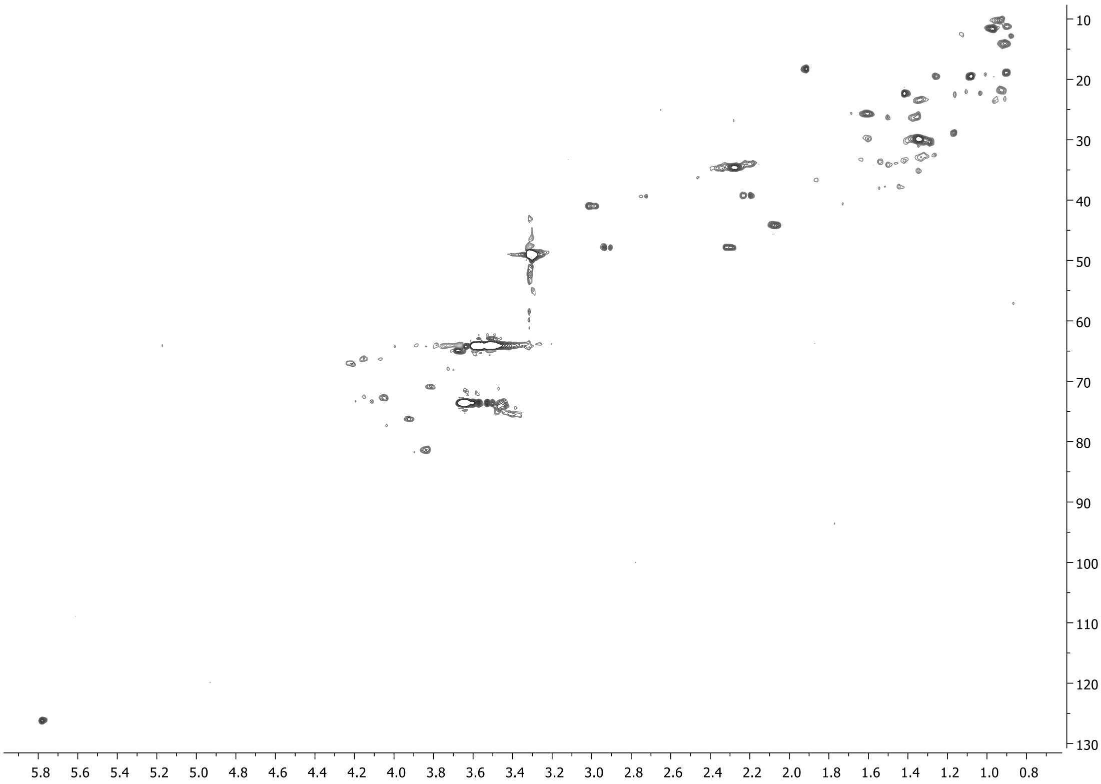

Figure S22. *g*HSQC spectrum (500 MHz, CD_3OD) of compound **4**.

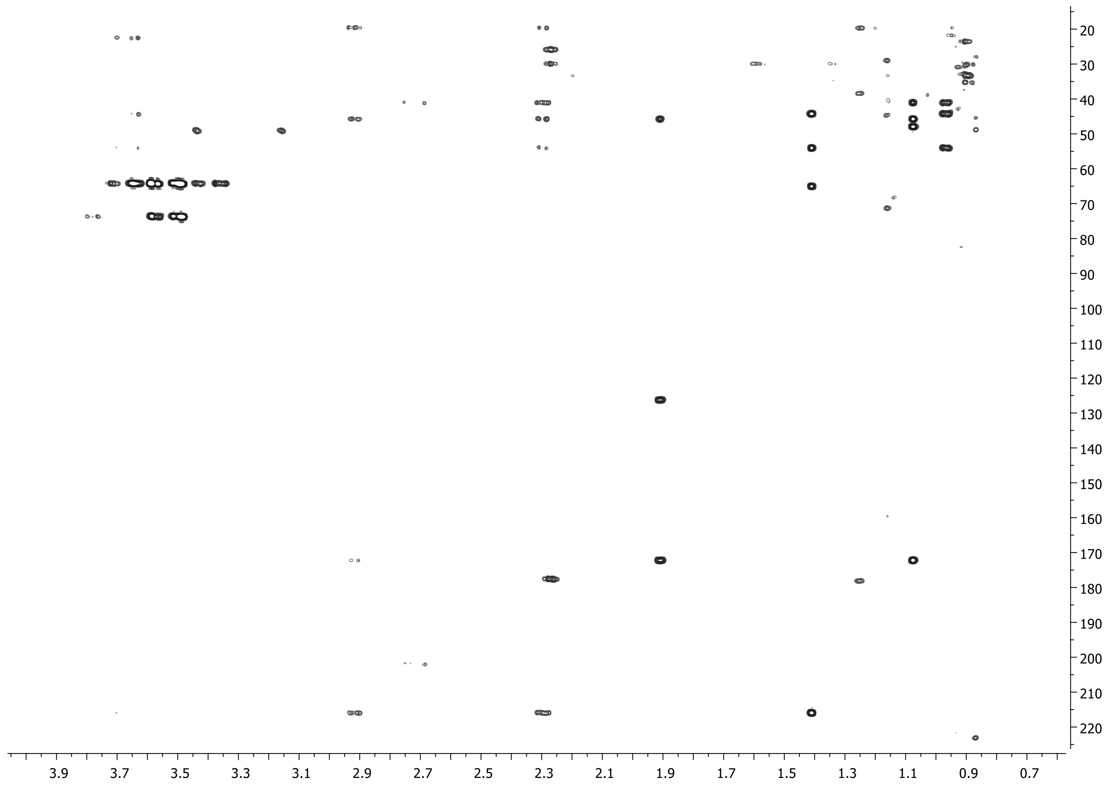

Figure S23. *g*HMBC spectrum (500 MHz, CD_3OD) of compound **4**.

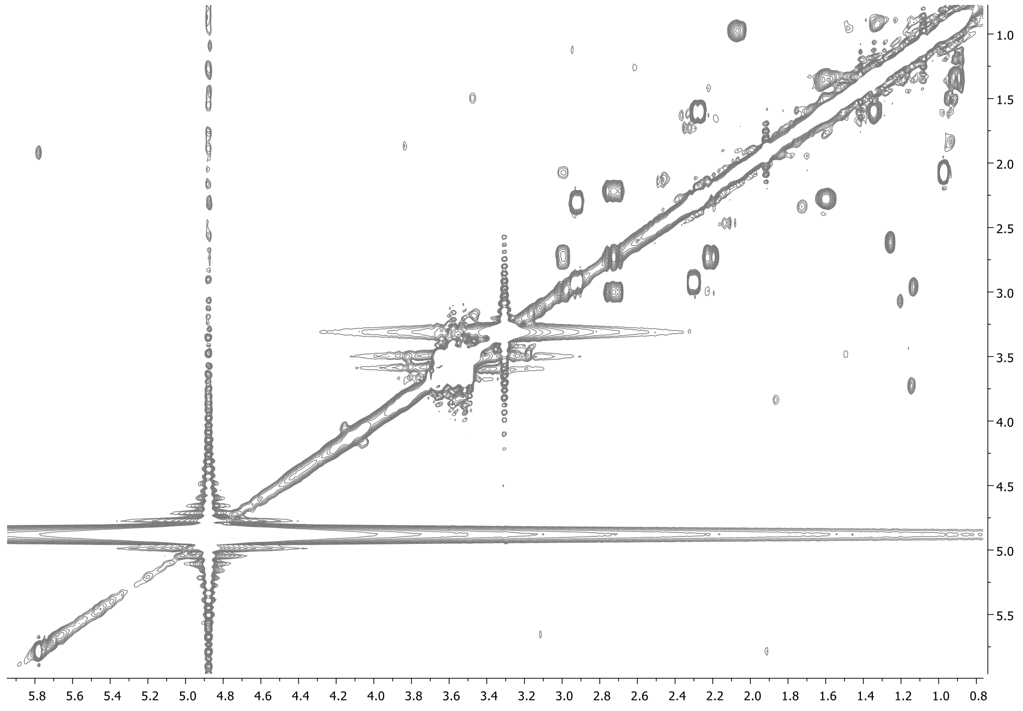

Figure S24. *g*COSY spectrum (500 MHz, CD_3OD) of compound **4**.

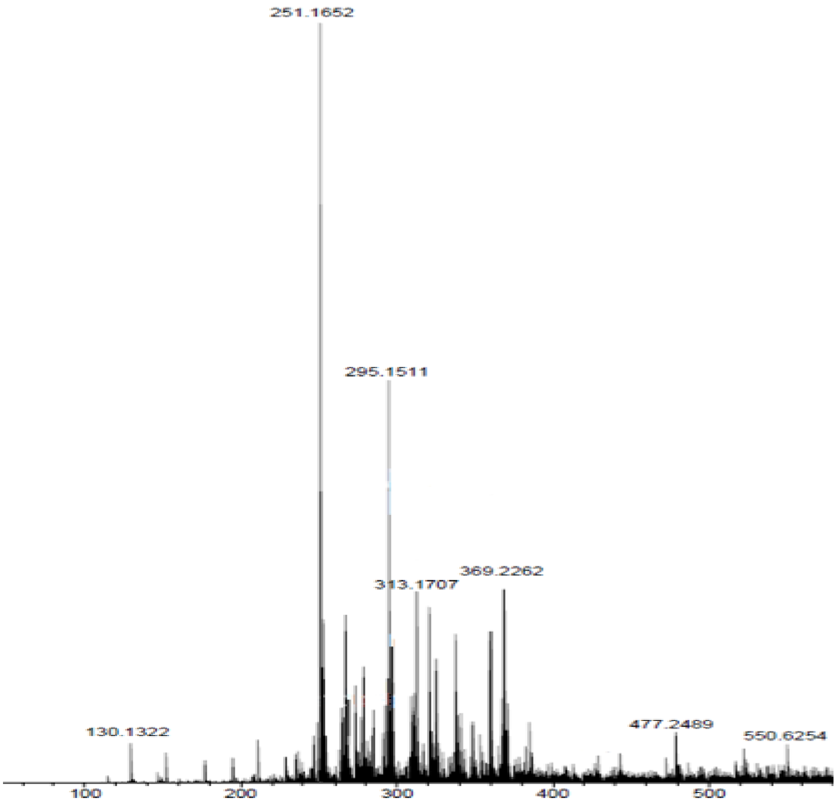

Figure S25. HR-ESI-MS spectrum of compound **4** (*m/z* 251.1652 $[M+H]^+$, calc.$C_{15}H_{23}O_3^+$ 251.16417, err. 4.1 ppm).

Table S5. NMR data of compound **5**

Position	δ ^{13}C [c]	δ ^1H (mult., *J* Hz, integral)	*g*COSY	*g*HMBC	NOESY
**1**	39.9	2.59 (dd, 16.9, 14.5, 1H) - β<br>2.03 (dd, 16.9, 2.7, 1H) - α	H-1α, H-10<br>H-1β	C-2, C-10	H-1α, H-13<br>H-1β
**2**	200.4	-	-	-	-
**3**	126.0	5.71 (br s, 1H)	H-11[d]	C-1/C-5	-
**4**	171.6	-	-	-	-
**5**	39.9	-	-	-	-
**6**	30.4	1.65 (m, 1H) - a overlapped<br>1.53 (m, 1H) - b overlapped	overlapped	-	-<br>-
**7**	26.0	1.58 (m, 1H) - a overlapped<br>1.24 (m, 1H) - b	H-7b<br>H-7a	-	-<br>-
**8**	37.1	-	-	-	-
**9**	39.7	1.51 (m, 1H) overlapped	H-13	-	-
**10**	40.2	2.38 (dt, 14.5, 2.7, 1H)	H-1β	-	H-15
**11**	18.5	1.88 (br s, 3H)	H-3[d]	C-3, C-4, C-5	-
**12**	18.0	1.08 (s, 3H)	-	C-4, C-6	H-14b
**13**	11.4	0.93 (d, 7.5, 3H)	H-9	C-8, C-9	H-1β, H-14a
**14**	77.0	3.52 (d, 9.4, 1H) - a<br>2.96 (d, 9.4, 1H) - b	H-14b<br>H-14a	C-7, C-8, C-9, C-15, C-1’	H-13, H-14b<br>H-14a, H-1’
**15**	22.6	1.08 (s, 3H)	-	C-7, C-8, C-9, C-14	H-10
**1’**	99.2	4.84 (d, 3.4, 1H)	H-2’α	-	H-14b, H-2’α
**2’**	33.2	1.94 (dd, 12.5, 5.1, 1H) - β<br>1.77 (td, 12.5, 3.4, 1H) - α	H-2’α, H-3’<br>H-1’, H-2’β, H-3’	C-3’, C-4	-<br>H-1’
**3’**	66.0	4.02 (m, 1H) [e]	H-2’α, H-2’β	-	-
**4’**	71.4	3.64 (br s, 1H)	-	-	-
**5’**	66.2	3.91 (q, 6.7, 1H)	H-6’	-	H-6’
**6’**	16.7	1.28 (d, 6.7, 3H)	H-5’	C-4’, C-5’	H-14b, H-5’

Acquired at 500 MHz in $CDCl_3$. α and β refer to planar orientation; a and b are used to distinguish each geminal hydrogen. [c] indirectly determined by *g*HSQC and *g*HMBC. [d] long range coupling (^4J) in *g*COSY. [e] in CD_3OD, 3.92 (ddd, 13.5, 5.0, 2.0)

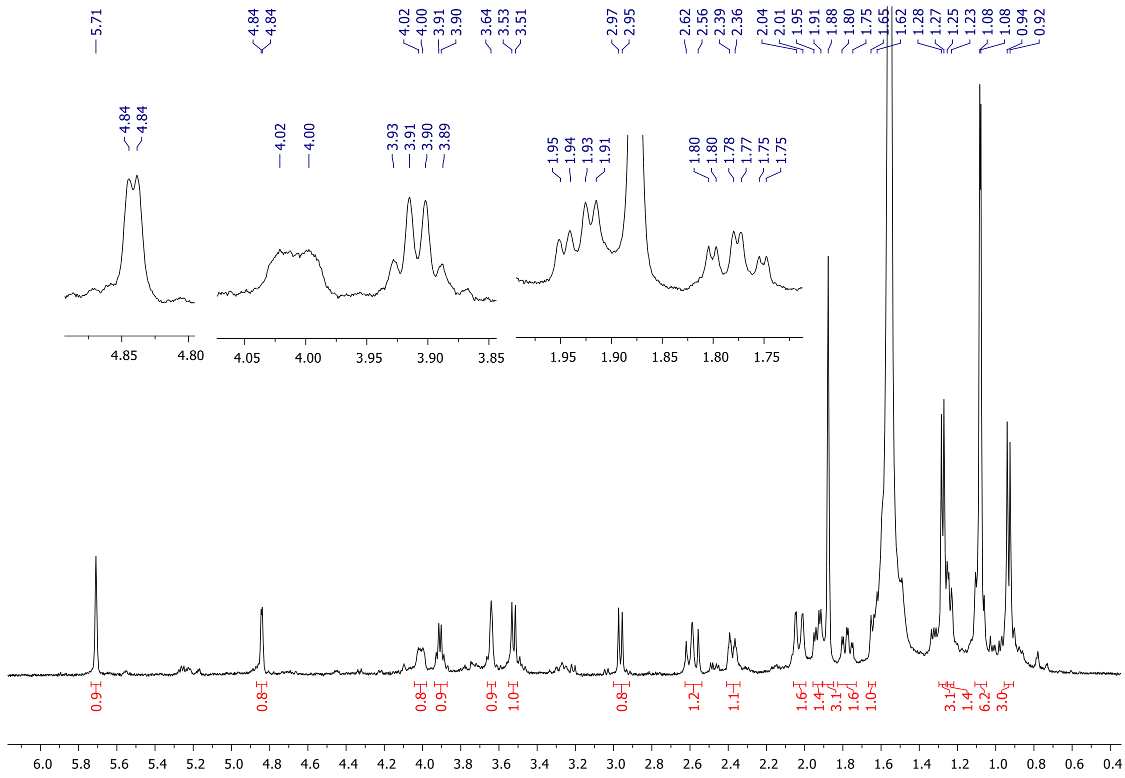

Figure S26. ^{1}H NMR spectrum (500 MHz, $CDCl_3$) of compound **5**.

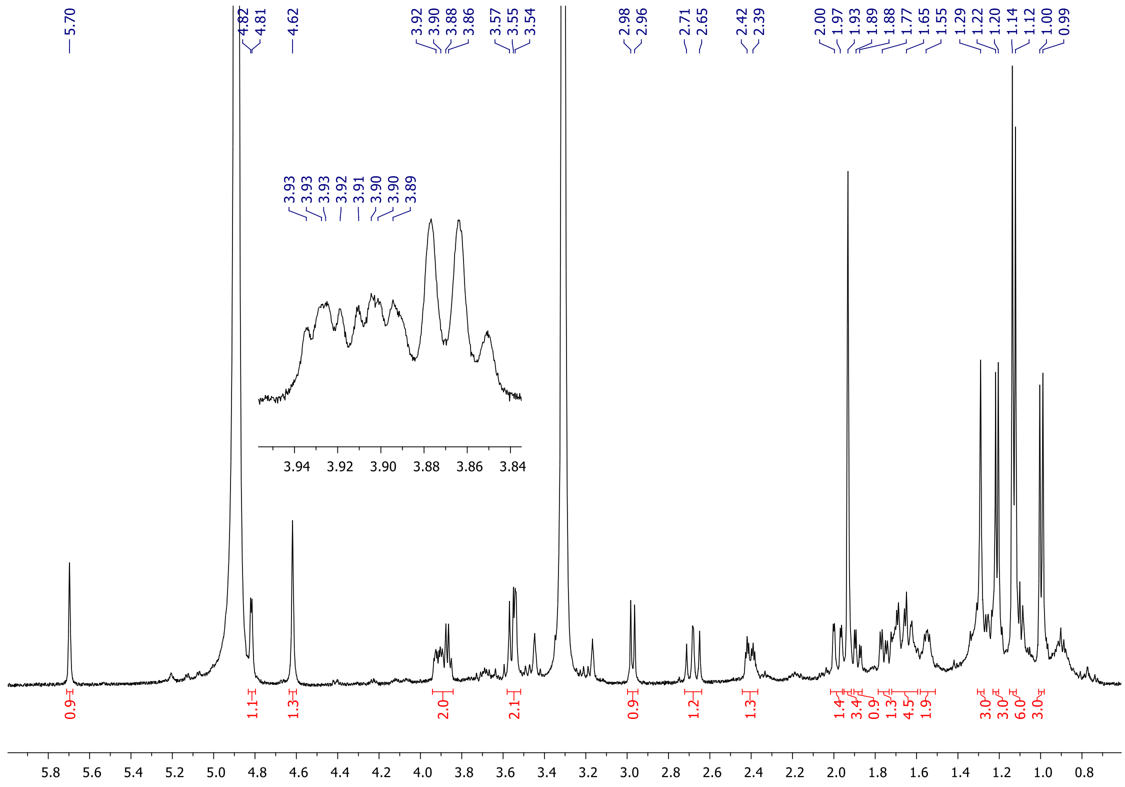

Figure S27. ^{1}H NMR spectrum (500 MHz, CD_3OD) of compound **5**.

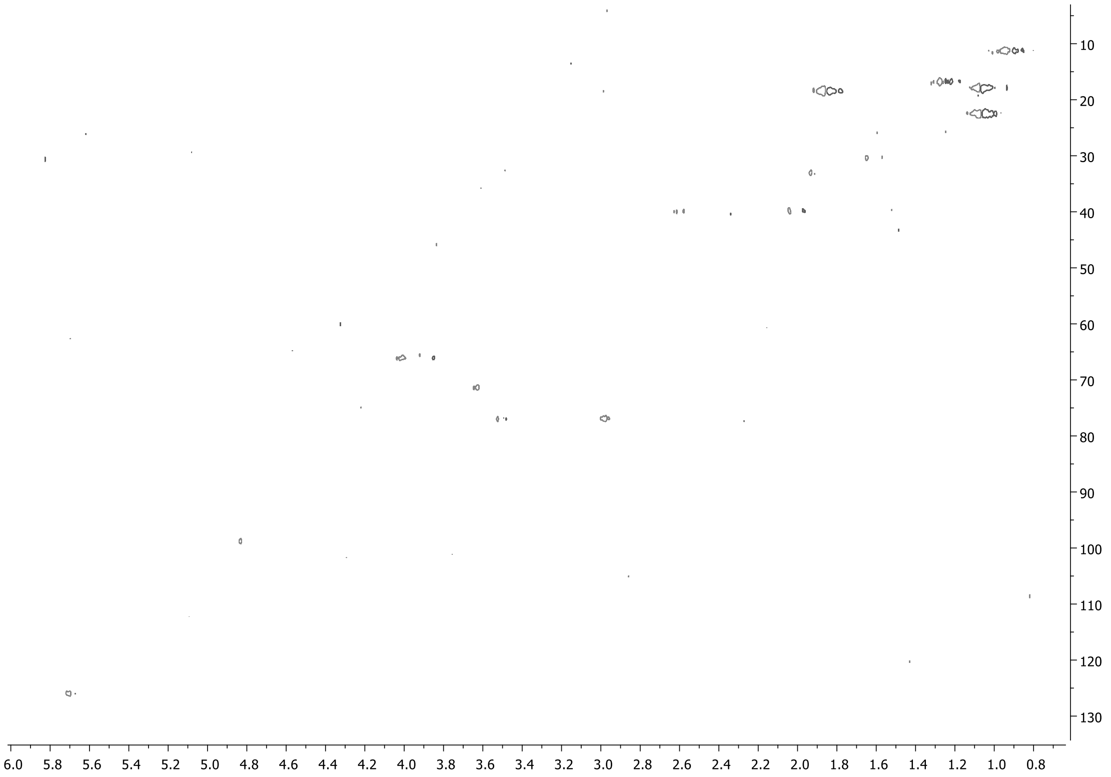

Figure S28. *g*HSQC spectrum (500 MHz, $CDCl_3$) of compound **5**.

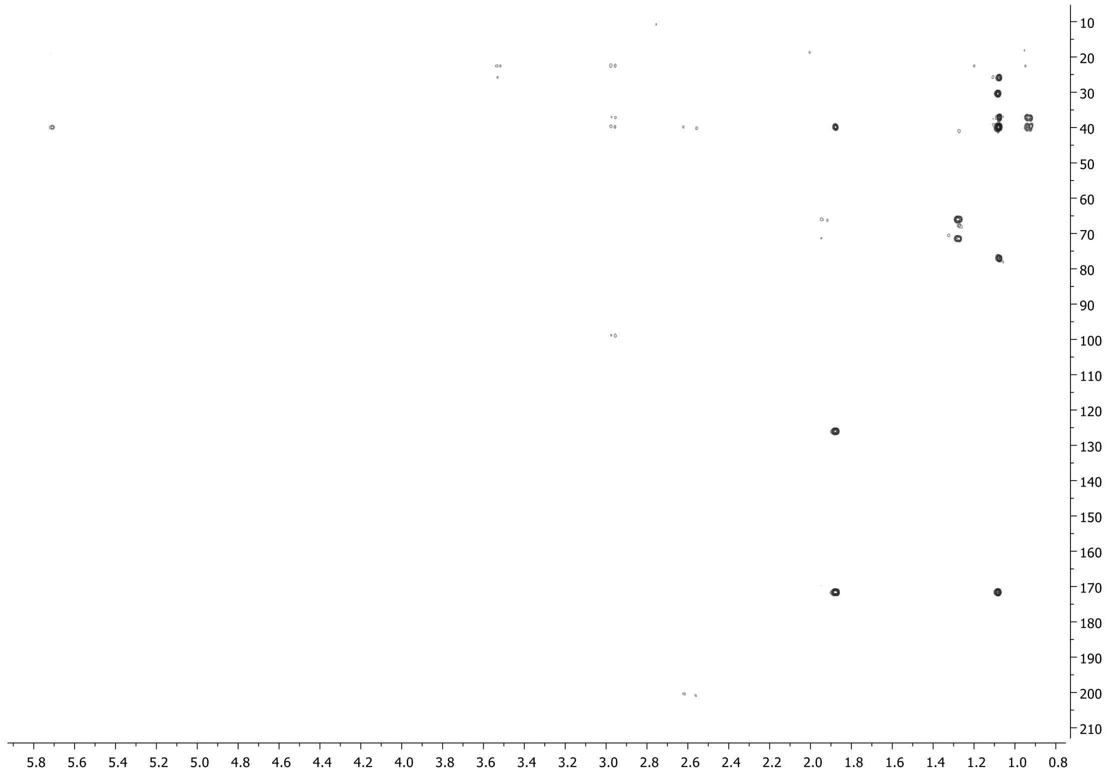

Figure S29. *g*HMQC spectrum (500 MHz, $CDCl_3$) of compound **5**.

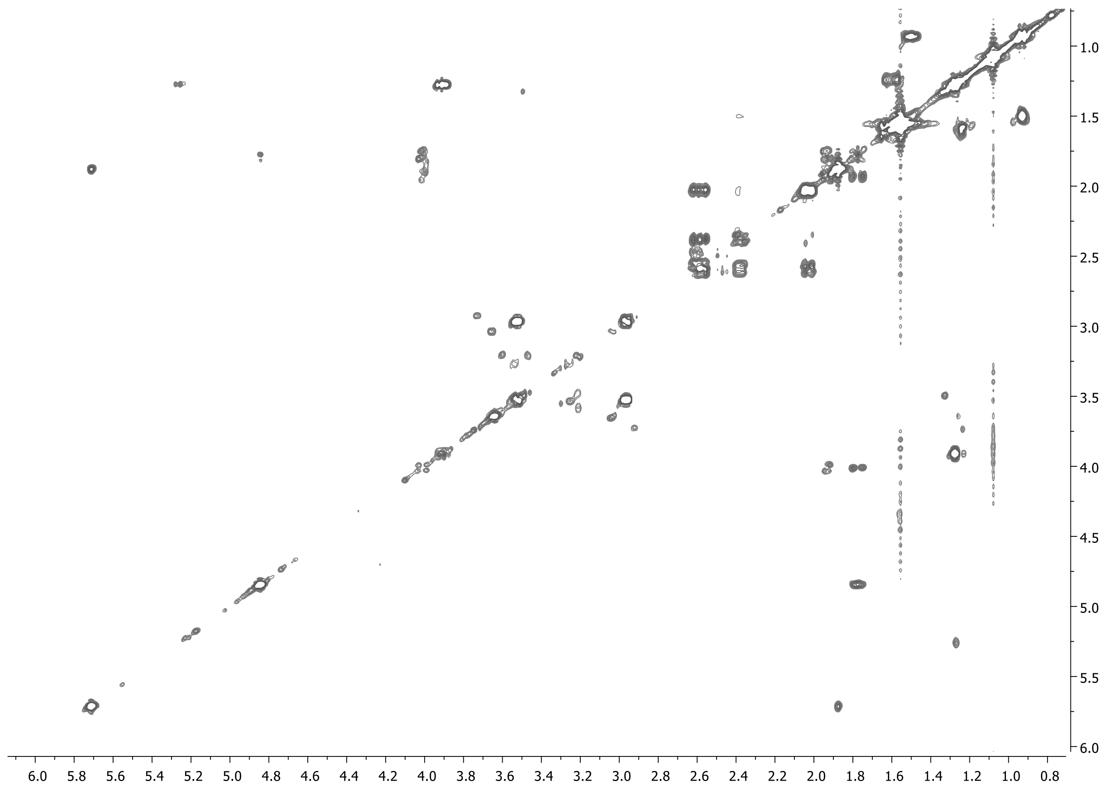

Figure S30. *g*COSY spectrum (500 MHz, $CDCl_3$) of compound **5**.

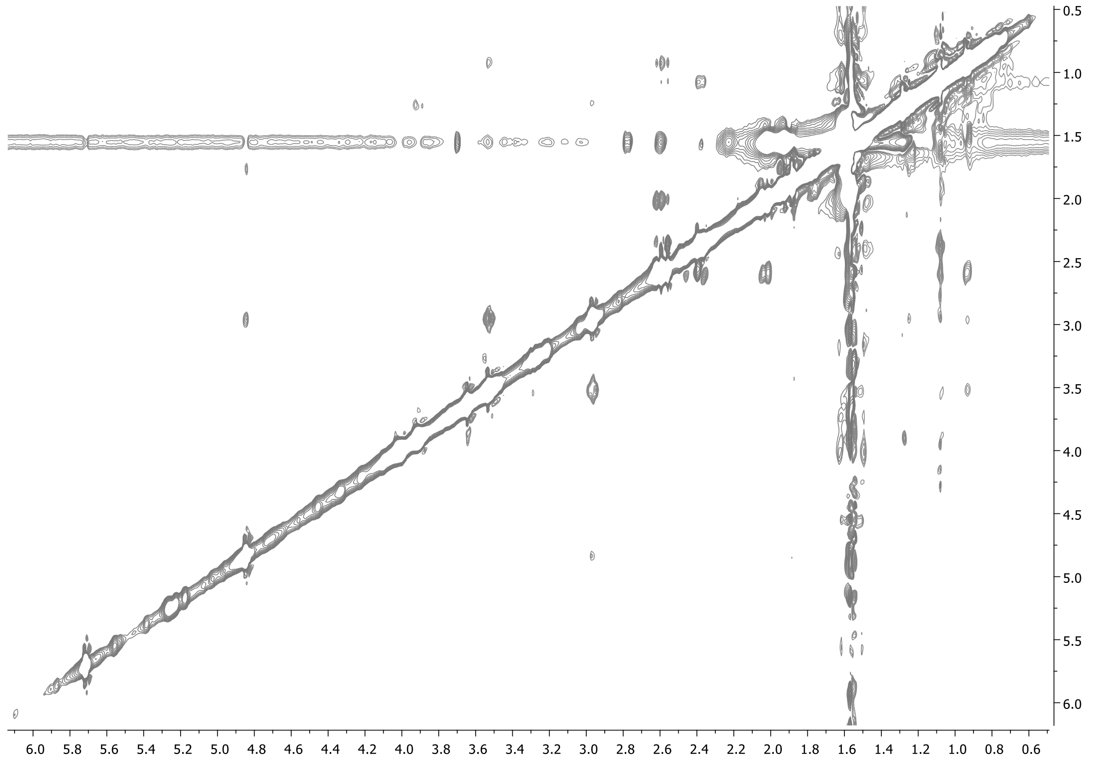

Figure S31. NOESY spectrum (500 MHz, $CDCl_3$) of compound **5**.

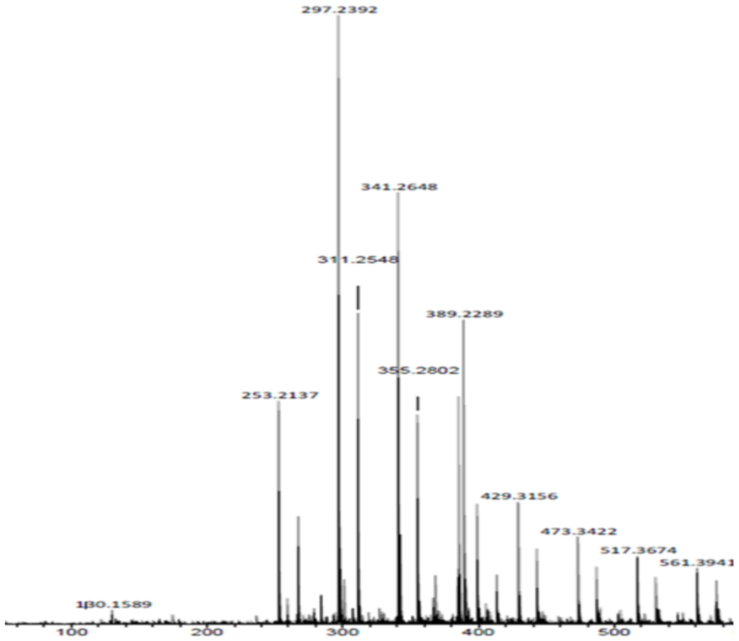

Figure S32. HR-ESI-MS spectrum of compound **5** (*m/z* 389.2289 [M+Na]$^+$, calc. $C_{21}H_{34}O_5Na^+$, 389.22985, err 2.4 ppm).

Table S6. NMR data of compound **6**

Position	δ ^{13}C ^{c}	δ ^{1}H (mult., *J* Hz, integral)	*g*COSY	*g*TOCSY	*g*HMBC	NOESY
**1**	40.4	2.67 (dd, 17.1, 14.7, 1H) - β<br>1.99 (dd, 17.1, 3.5, 1H) - α	H-1α, H-10<br>H-1β	H-1α, H-9,<br>H-10, H-13<br>H-1β, H-10	C-2	H-1α, H-10,<br>H-12, H-13<br>H-1β, H-9, H-10
**2**	203.4	-	-	-	-	-
**3**	125.9	5.70 (br s, 1H)	-	H-11	C-1, C-11	H-11
**4**	175.5	-	-	-	-	-
**5**	41.1	-	-	-	-	-
**6**	30.7	1.63 (m, 1H) - a<br>1.30 (m, 1H) – b*	-<br>-	-	-	-<br>-
**7**	26.3	1.62 (m, 1H) - a<br>1.18 (m, 1H) - b	-<br>-	H-14b<br>H-7a	C-6	-<br>-
**8**	37.5	-	-	-	-	-
**9**	40.7	1.55 (m, 1H)	H-13	-	-	H-1α, H-10, H-13
**10**	41.3	2.38 (dt, 14.7, 3.5, 1H)	H-1β	H-1α, H1β,<br>H-13	C-4	H-1α, H-1β, H-9, H-15
**11**	18.5	1.93 (d, 1.1, 3H)	-	-	C-3, C-4	H-3
**12**	17.8	1.13 (s, 3H)	-	-	C-4, C-5,<br>C-6, C-10	H-1β, H-14a, H-14b
**13**	11.2	0.97 (d, 7.7, 3H)	H-9	H-1α, H-1β,<br>H-9, H-10	C-8, C-9,<br>C-10	H-1β, H-9, H-14a
**14**	78.3	3.59 (d, 9.1, 1H) - a<br>3.28 (d, 9.1, 1H) - b	H-14b<br>H-14a	H-14b<br>H-14a	C-15, C-8,<br>C-1’	H-7b, H-12, H-13, H-14b<br>H-12, H-13, H-1’
**15**	22.6	1.12 (s, 3H)	-	-	C-7, C-8,<br>C-9	H-10
**1’**	101.9	4.41 (dd, 9.8, 1.5, 1H)	H-2’α	H-2’a, H-2’b,<br>H-3’	C-14	H-14b, H-2’a, H-2’b,<br>H-3’, H-5’
**2’**	35.1	1.80 (m, 1H) - b<br>1.70 (m, 1H) - a	H-2’a<br>H-1’, H-2’b, H-3’	-	C-1’, C-3’,<br>C-4’	H-1’, H-3’<br>H-1’, H-3’
**3’**	69.8	3.68 (dt,12.4, 3.0, 1H)	H-2’a	H-1’, H-2’a,<br>H-2’b, H-4’	-	H-1’, H-2’a, H-2’b, H-4’,<br>H-5’
**4’**	71.2	3.45 (br s, 1H)	-	H-1’, H-2’a	-	H-3’, H-6’
**5’**	71.7	3.50 (q, 6.4, 1H)	H-6’	-	C-1’	H-1’, H-3’, H-6’
**6’**	16.7	1.27 (d, 6.4, 3H)	H-5’	H-5’	C-4’	H-4’, H-5’

Acquired at 500 MHz in CD_3OD. α and β refer to planar orientation; a and b are used to distinguish each geminal hydrogen. [c] indirectly determined by *g*HSQC and *g*HMBC. *uncertain chemical shift.

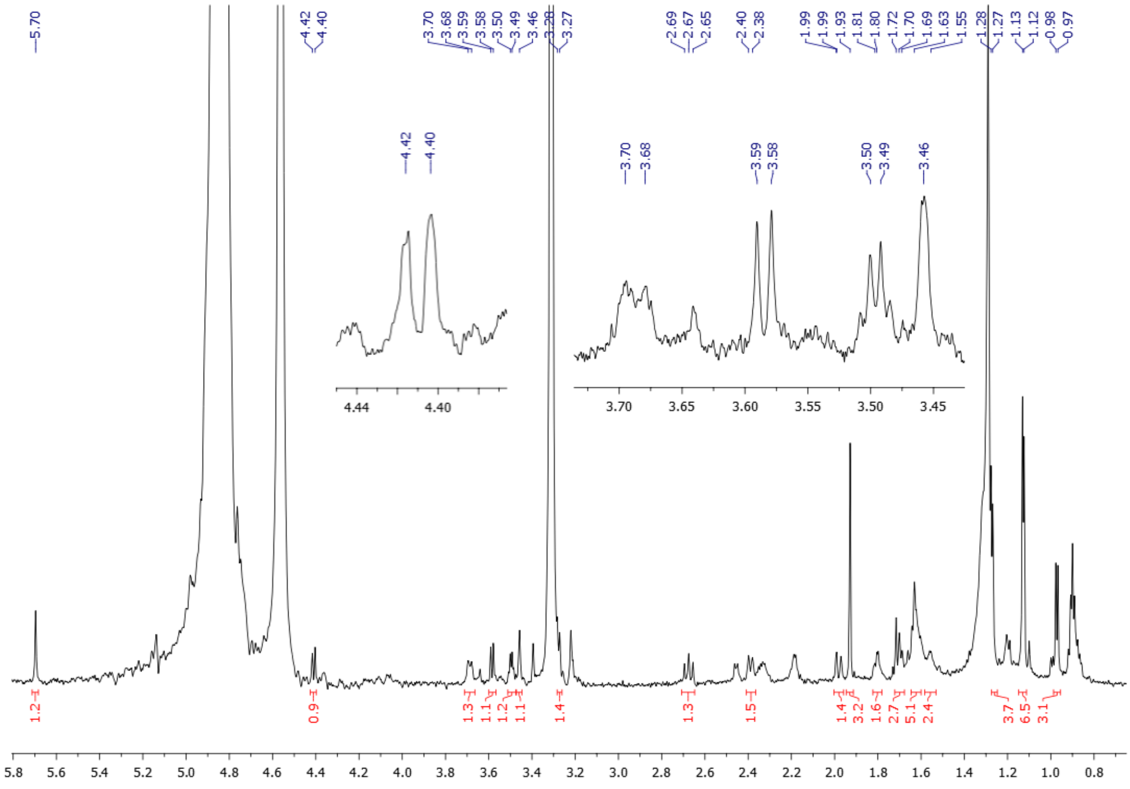

Figure S33. ^{1}H NMR spectrum (500 MHz, CD_3OD) of compound **6**. Signals corresponding to this compound are assigned.

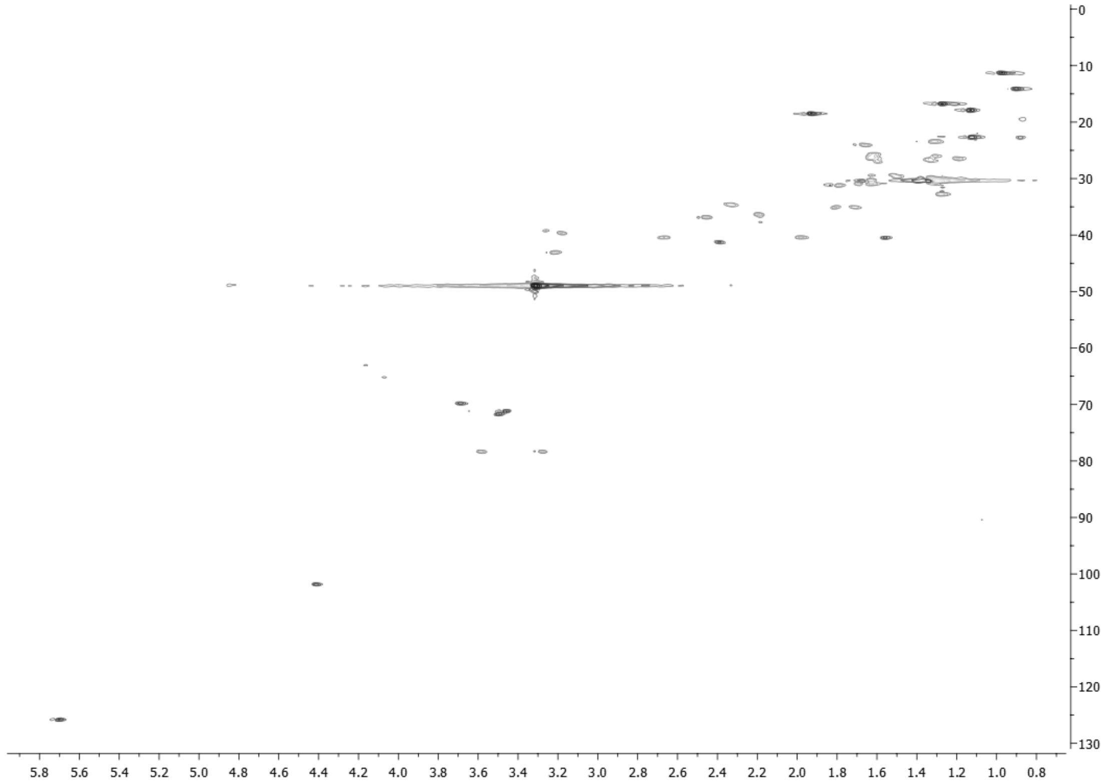

Figure S34. *g*HSQC spectrum (500 MHz, CD_3OD) of compound **6**.

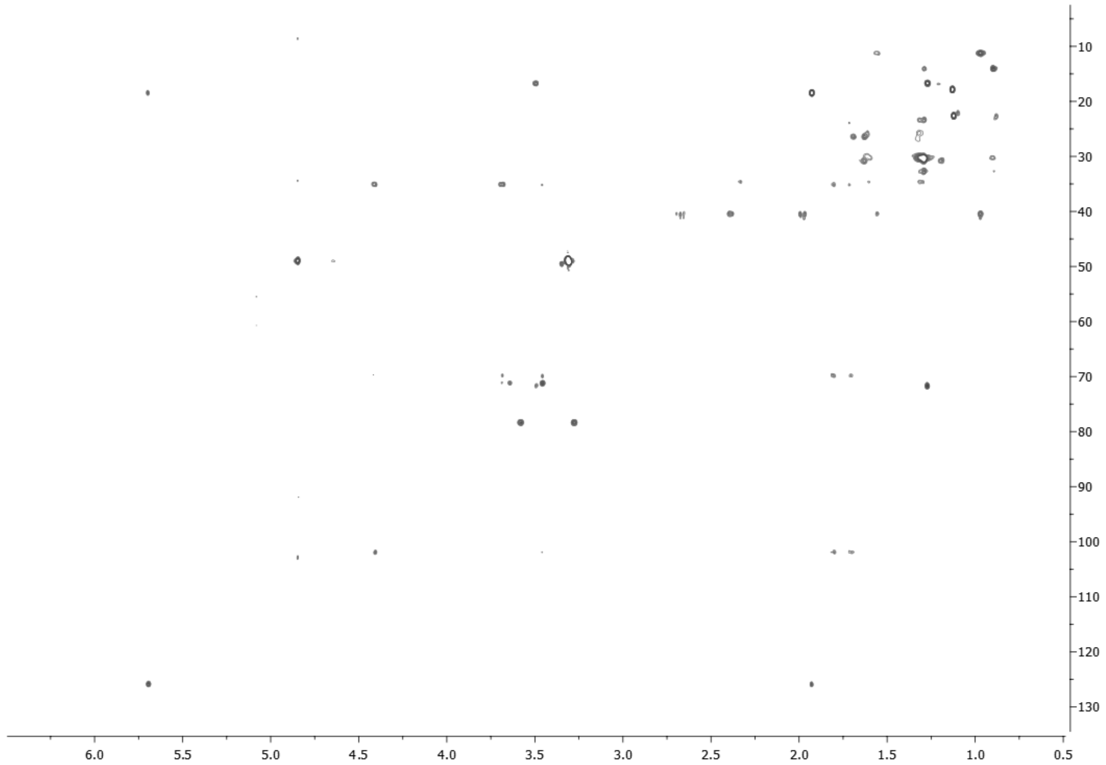

Figure S35. *g*HMBC spectrum (500 MHz, CD_3OD) of compound **6**.

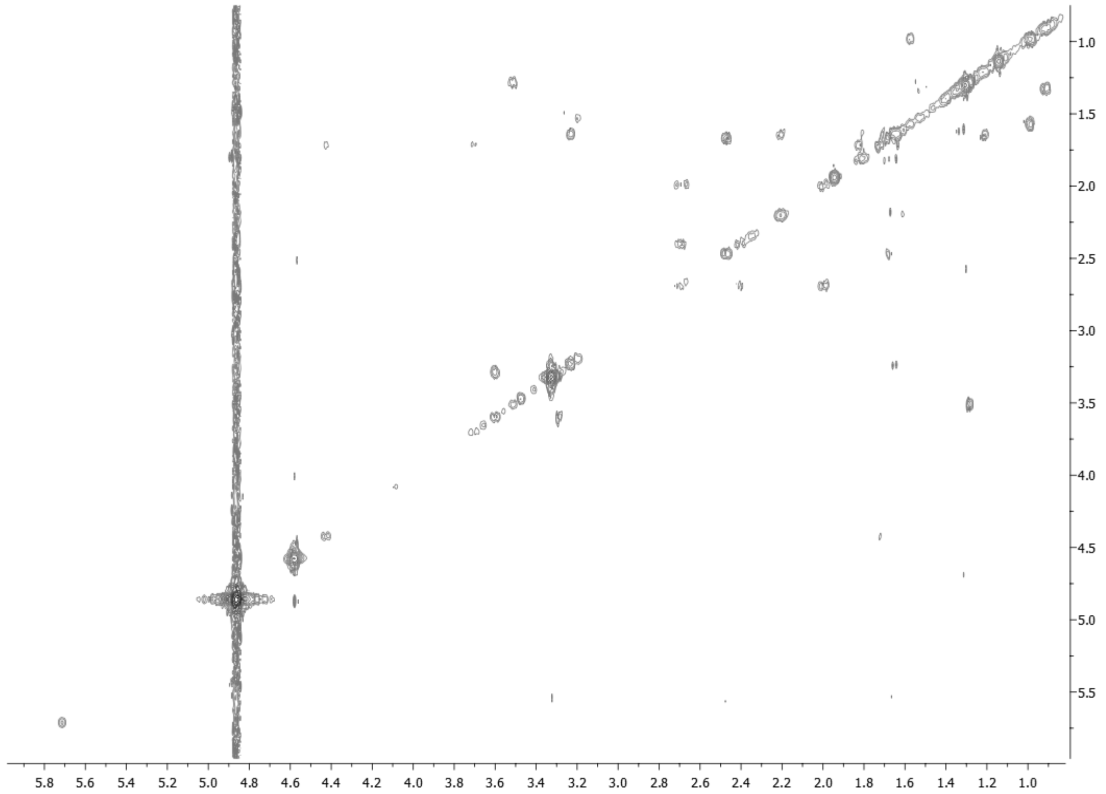

Figure S36. *g*COSY spectrum (500 MHz, CD_3OD) of compound **6**.

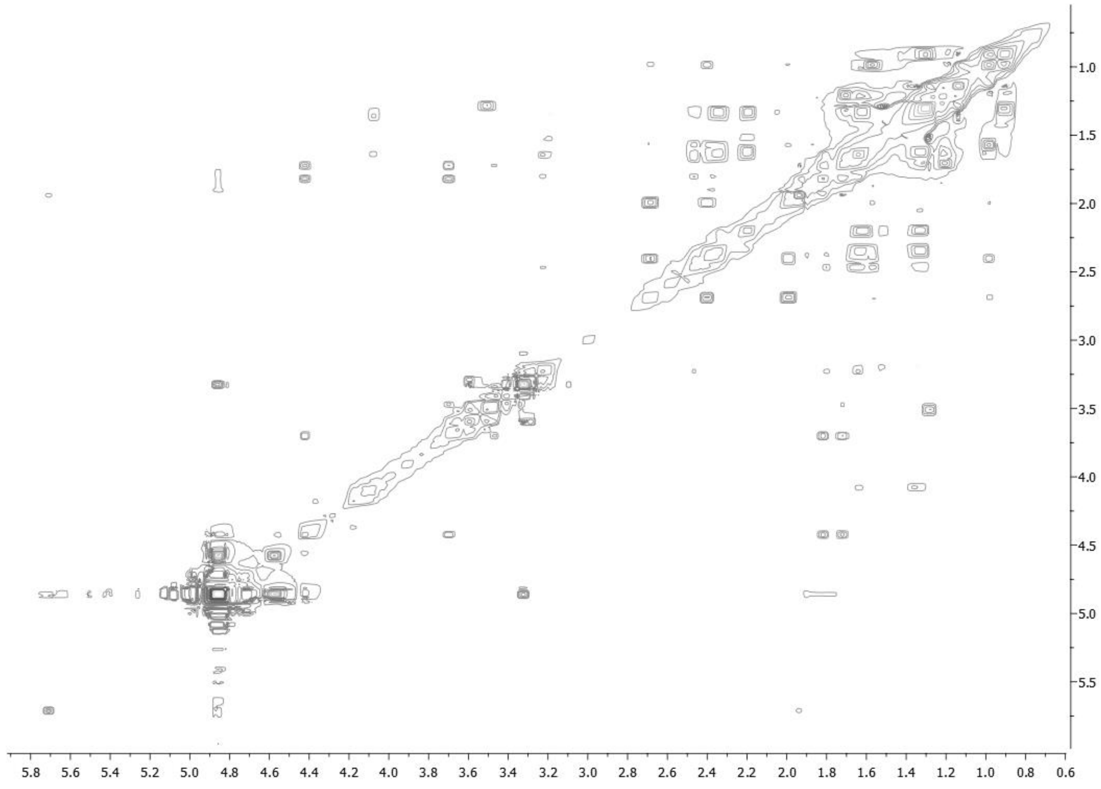

Figure S37. *g*TOCSY spectrum (500 MHz, CD_3OD) of compound **6**.

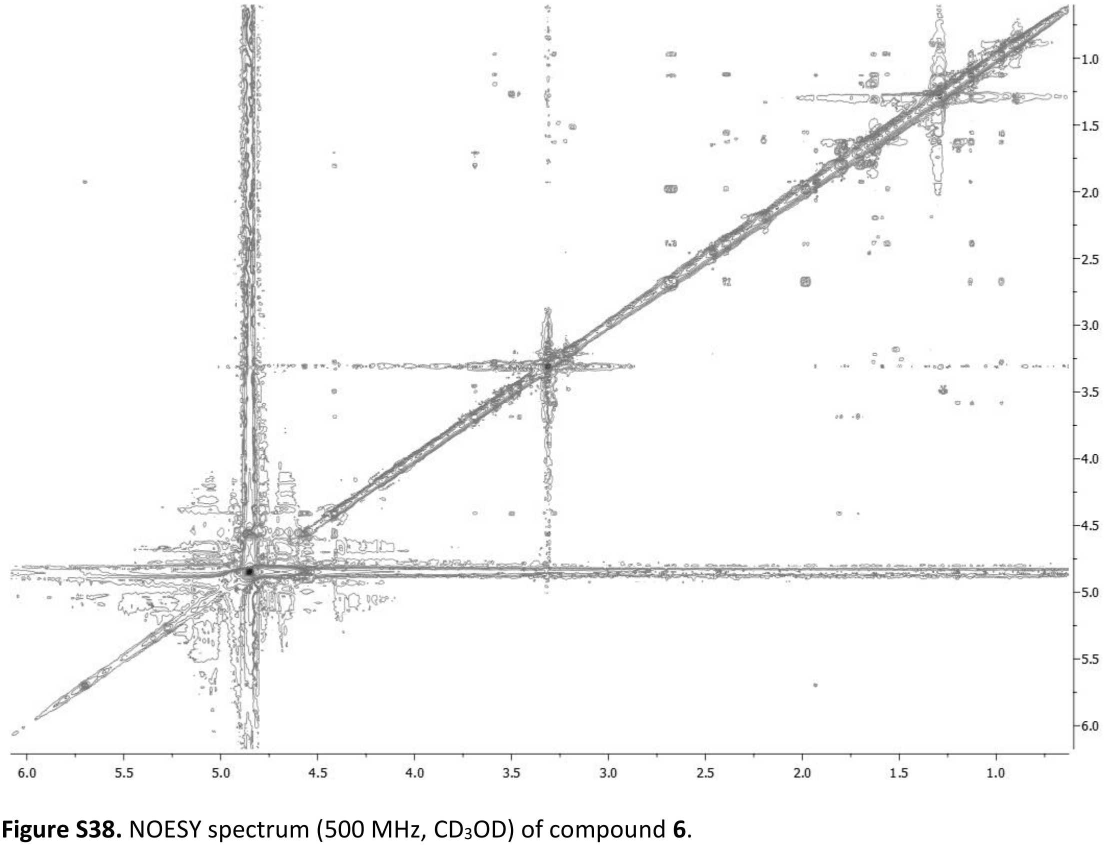

Figure S38. NOESY spectrum (500 MHz, CD_3OD) of compound **6**.

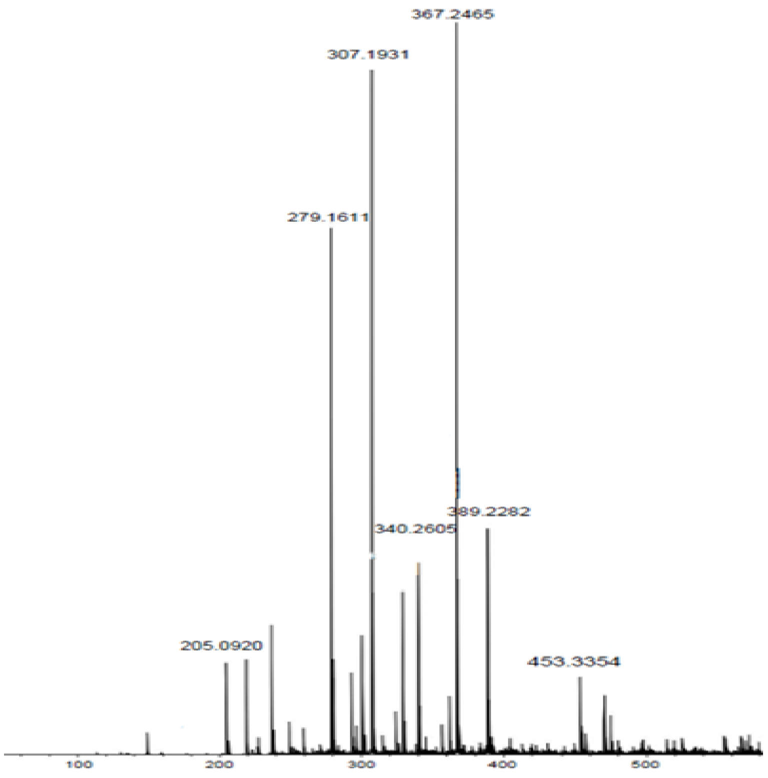

Figure S39. HR-ESI-MS spectrum of compound **6** (*m/z* 367.2465 $[M+H]^+$, calc. $C_{21}H_{35}O_5^+$, 367.24790, err 3.8 ppm; and *m/z* 389.2282 $[M+Na]^+$, calc. $C_{21}H_{34}O_5Na^+$, 389.22985, err 4.2 ppm).

Table S7. NMR data of compound **7**

Position	δ ^{13}C [c]	δ ^{1}H (mult., *J* Hz, integral)	*g*COSY	*g*TOCSY	*g*HMBC	NOESY
**1**	40.7	2.67 (dd, 16.9, 14.7, 1H) - β<br>1.97 (dd, 16.9, 3.5, 1H) - α	H-1α, H-10<br>H-1β	H-1α, H-10, H-13<br>H-13	-	H-1α<br>H-1β
**2**	n.o.	-	-	-	-	-
**3**	125.9	5.69 (br s, 1H)	H-11[d]	H-11	-	H-11
**4**	176.0	-	-	-	-	-
**5**	41.1	-	-	-	-	-
**6**	31.1	1.69 (m, 1H) - a<br>1.60 (m, 1H) - b	-<br>-	-<br>-	-	-<br>-
**7**	26.6	1.65 (m, 1H) - a<br>1.21 (m, 1H) - b	H-7b<br>H-7a	-<br>-	-	-<br>-
**8**	37.9	-	-	-	-	-
**9**	40.8	1.49 (m, 1H)	H-13	-	-	-
**10**	41.5	2.39 (dt, 14.7, 3.5, 1H)	H-1β	H-1α, H-1β, H-13	-	-
**11**	18.5	1.93 (d, 1.1, 3H)	H-3[d]	-	C-3, C-4, C-5	H-3
**12**	17.9	1.13 (s, 3H)	-	H-7b	C-4, C-6, C-10	-
**13**	11.2	1.00 (d, 7.7, 3H)	H-9	H-1α, H-1β, H-10	C-8, C-10	-
**14**	77.9	3.71 (d, 9.4, 1H) - a<br>2.99 (d, 9.4, 1H) - b	H-14b<br>H-14a	-<br>-	-	H-3’, H-14b<br>H-5’, H-14a
**15**	22.6	1.09 (s, 3H)	-	-	C-7, C-8, C-14	-
**1’**	105.9	5.13 (dd, 5.3, 1.5, 1H)	H-2’a	H-2’a, H-2’b, H-3’	-	H-2’a, H-6’, H-14b
**2’**	42.7	2.19 (ddd, 13.1, 5.2, 1.5, 1H) - b<br>2.02 (ddd, 13.1, 6.9, 5.3, 1H) - a	H-2’a, H-3’<br>H-1’, H2’b, H-3’	H-1’<br>H-1’	C-3’	H-7b, H-2’a<br>H-1’, H-2’b
**3’**	72.8	4.24 (dt, 6.9, 5.2, 1H)	H-2’a, H-2’b, H-4’	H-2’a, H-2’b, H-4’, H-5’, H-6’	-	H-14a
**4’**	91.0	3.57 (dd, 6.4, 5.2, 1H)	H-3’, H-5’	H-3’, H-2’a, H-2’b, H-6’	-	H-6’
**5’**	70.6	3.68 (quint., 6.4, 1H)	H-4’, H-6’	H-3’, H-6’	-	H-14b, H-6’
**6’**	19.0	1.19 (d, 6.4, 3H)	H-5’	H-3’, H-4’, H-5’	C-4’, C-5’	H-1’, H-4’, H-5’

Acquired at 500 MHz in CD_3OD. α and β refer to planar orientation; a and b are used to distinguish each geminal hydrogen. [c] indirectly determined by *g*HSQC and *g*HMBC. [d] long range coupling (^4J) in *g*COSY.

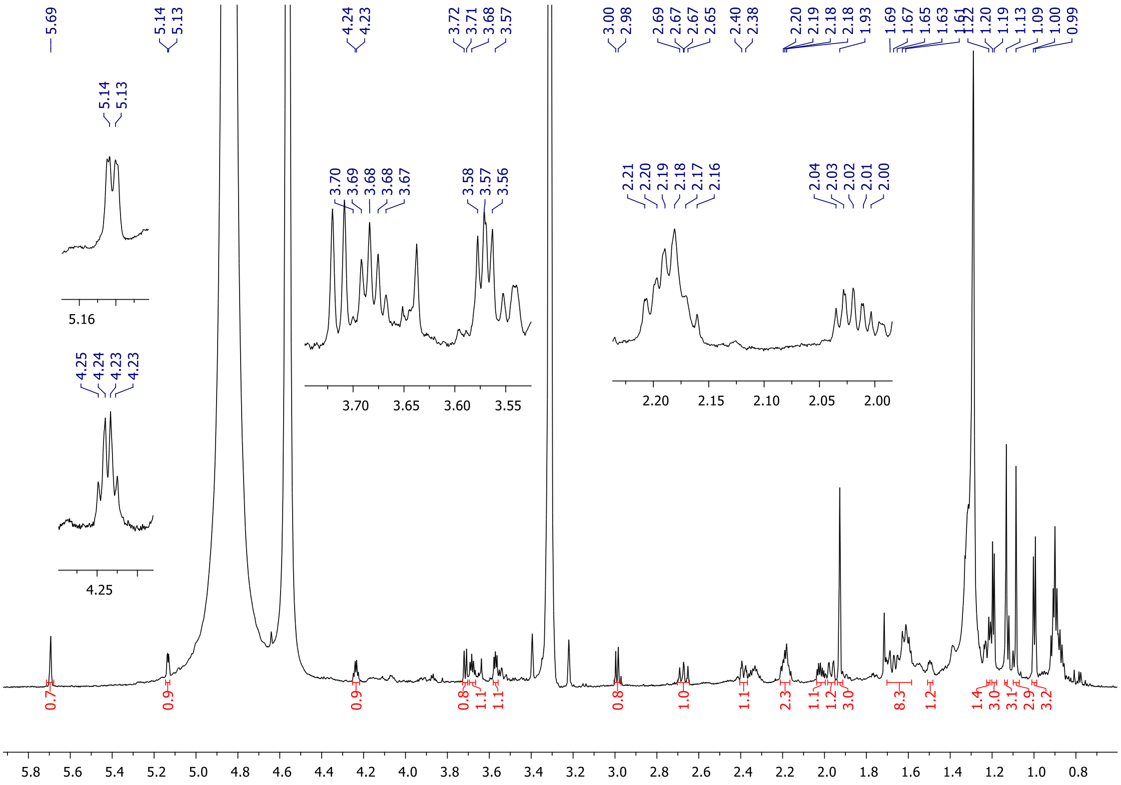

Figure S40. ^{1}H NMR spectrum (500 MHz, CD_3OD) of compound **7**. Signals from this compound are assigned.

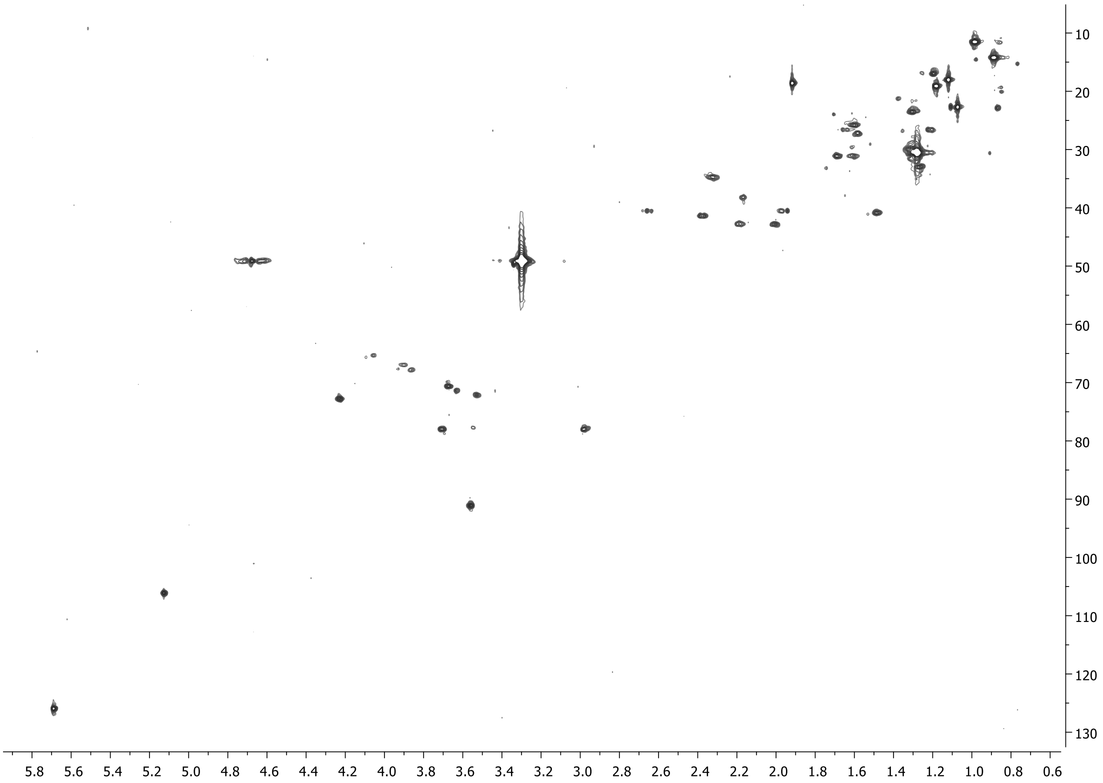

Figure S41. *g*HSQC spectrum (500 MHz, CD_3OD) of compound **7**.

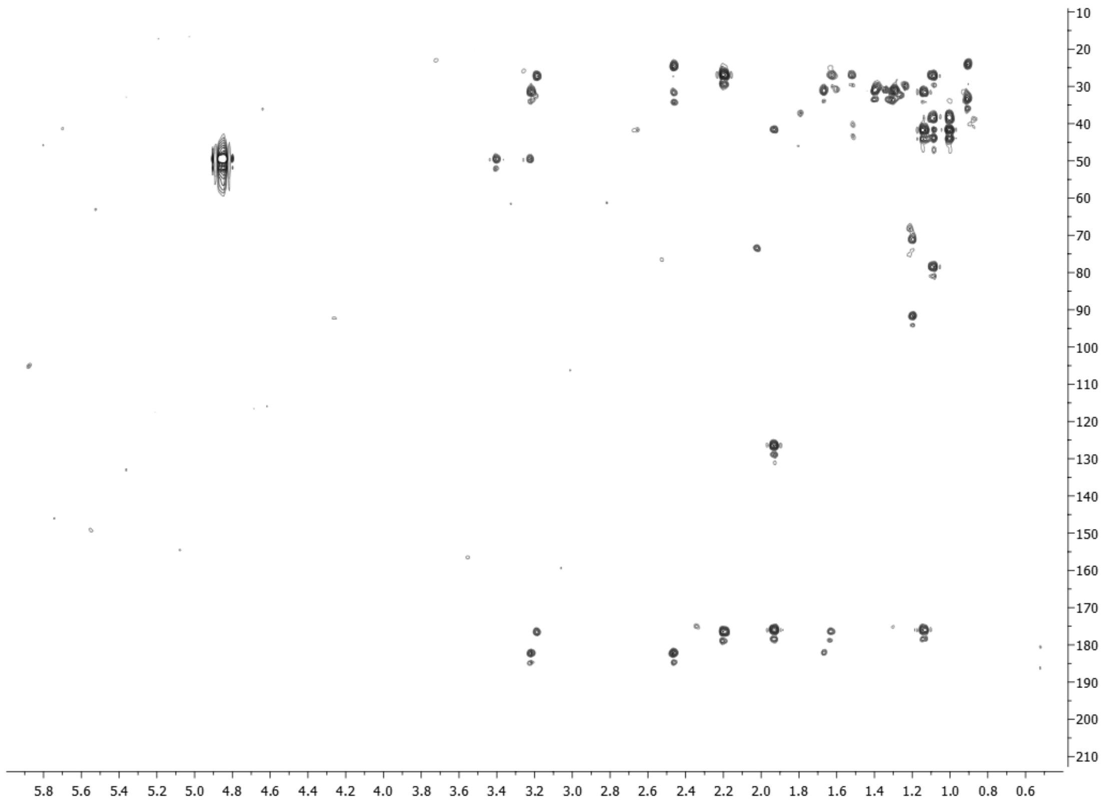

Figure S42. *g*HMBC spectrum (500 MHz, CD_3OD) of compound **7**.

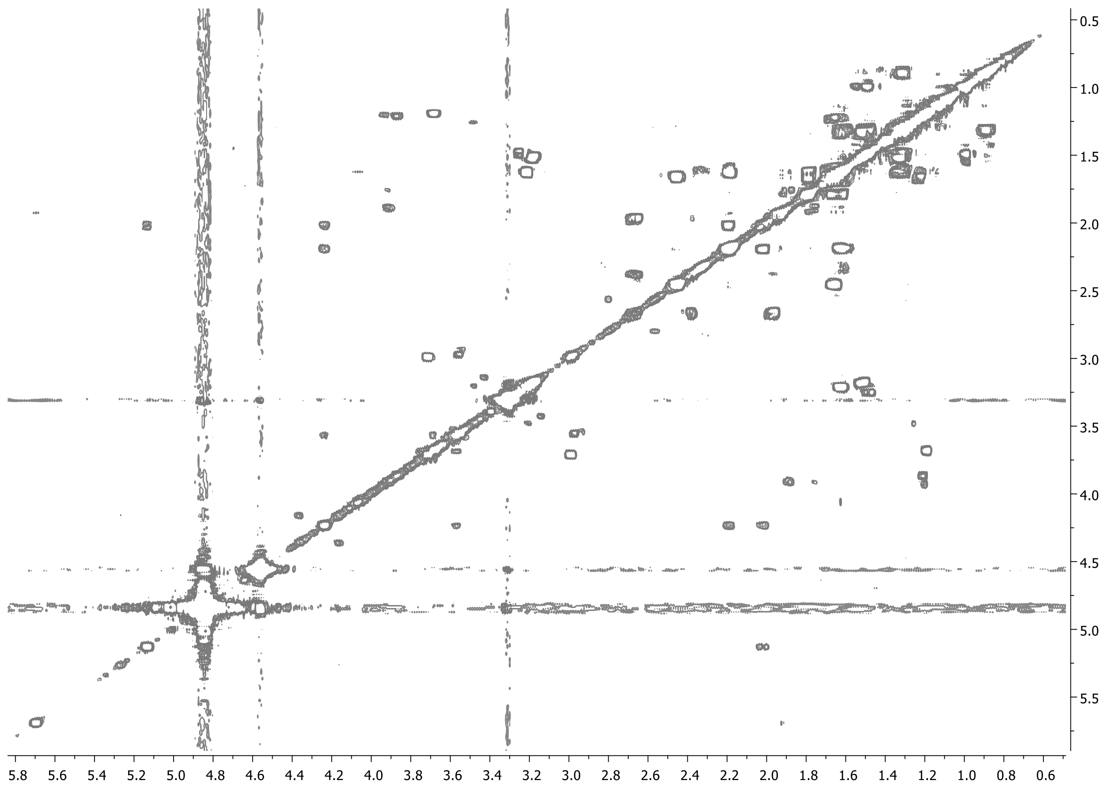

Figure S43. *g*COSY spectrum (500 MHz, CD_3OD) of compound **7**.

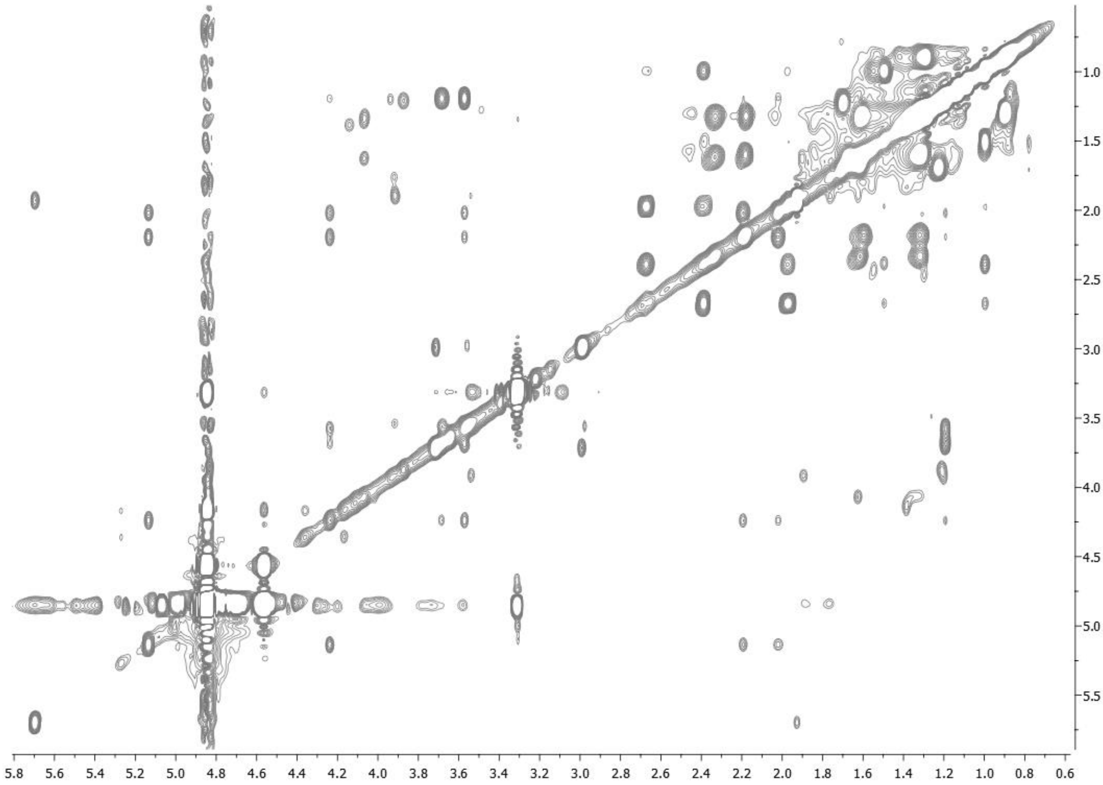

Figure S44. *g*TOCSY spectrum (500 MHz, CD_3OD) of compound **7**.

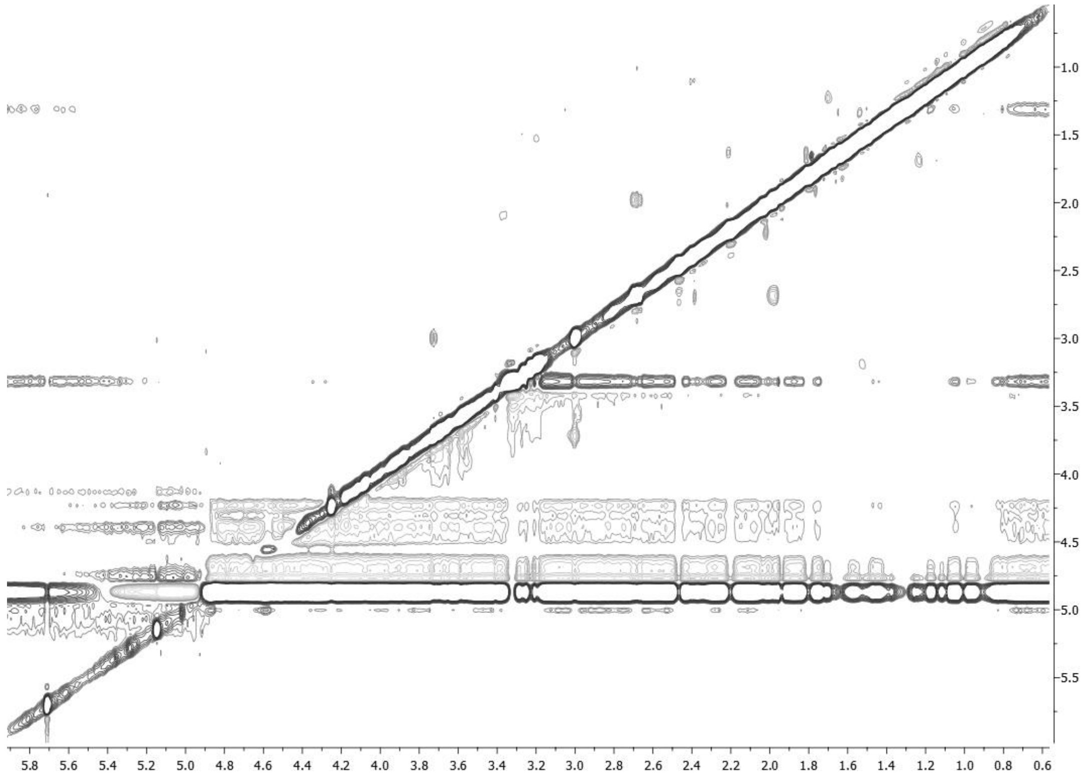

Figure S45. NOESY spectrum (500 MHz, CD_3OD) of compound **7**.

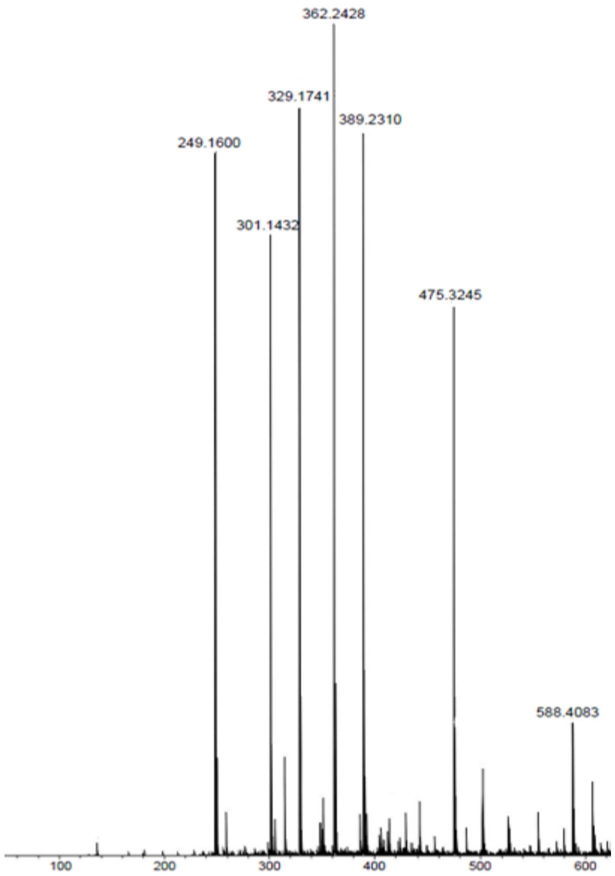

Figure S46. HR-ESI-MS spectrum of compound **7** (*m/z* 389.2310 $[M+Na]^+$, calc. $C_{21}H_{34}O_5Na^+$, 389.22985, err 3.0 ppm).

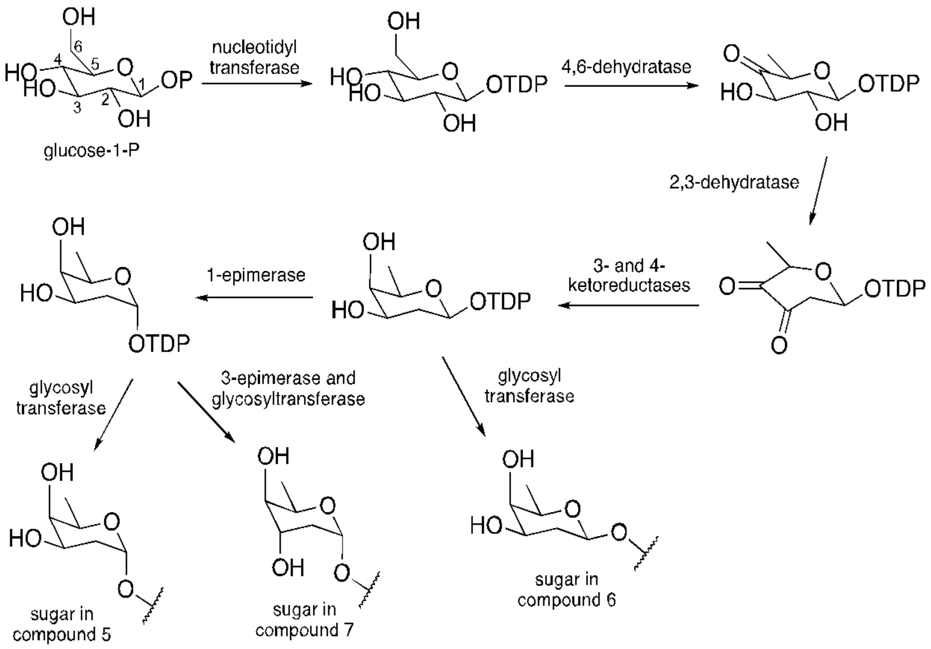

Figure S47. Possible steps in the biosynthesis of the sugars found in olindenones E-G (**5-7**).

Table S8. ^{13}C NMR (CD_3OD, 125 MHz) of ^{13}C-enriched **1** from *S. olindensis* DAUFPE 5622 cultivation with glucose-1-^{13}C or pyruvate-3-^{13}C

Position	^{13}C	glucose-1-^{13}C	pyruvate-3-^{13}C
**1**	41.3	1.1	1.0
**2**	203.3	**5.1**	1.4
**3**	126.1	1.1	1.1
**4**	175.6	1.1	1.2
**5**	40.8	1.6	1.3
**6**	31.4	**4.9**	1.1
**7**	26.6	1.2	1.2
**8**	38.6	1.1	1.3
**9**	40.6	1.1	1.1
**10**	41.7	1.5	1.2
**11**	18.8	1.3	1.1
**12**	18.2	**4.9**	**2.6**
**13**	11.5	**4.8**	**2.5**
**14**	71.4	**5.0**	1.1
**15**	22.3	**5.5**	**2.4**

* Positions with enriched carbons in each experiment are highlighted. In the glucose-1-^{13}C experiment, the average abundance of ^{13}C in the enriched positions increased to 5.0%, indicating an incorporation rate (L/U) almost five times higher than the natural ^{13}C-incorporation. In the pyruvate-3-^{13}C experiment, the abundance increased to ~2.5%, more than twice the natural ^{13}C abundance of 1.1%.

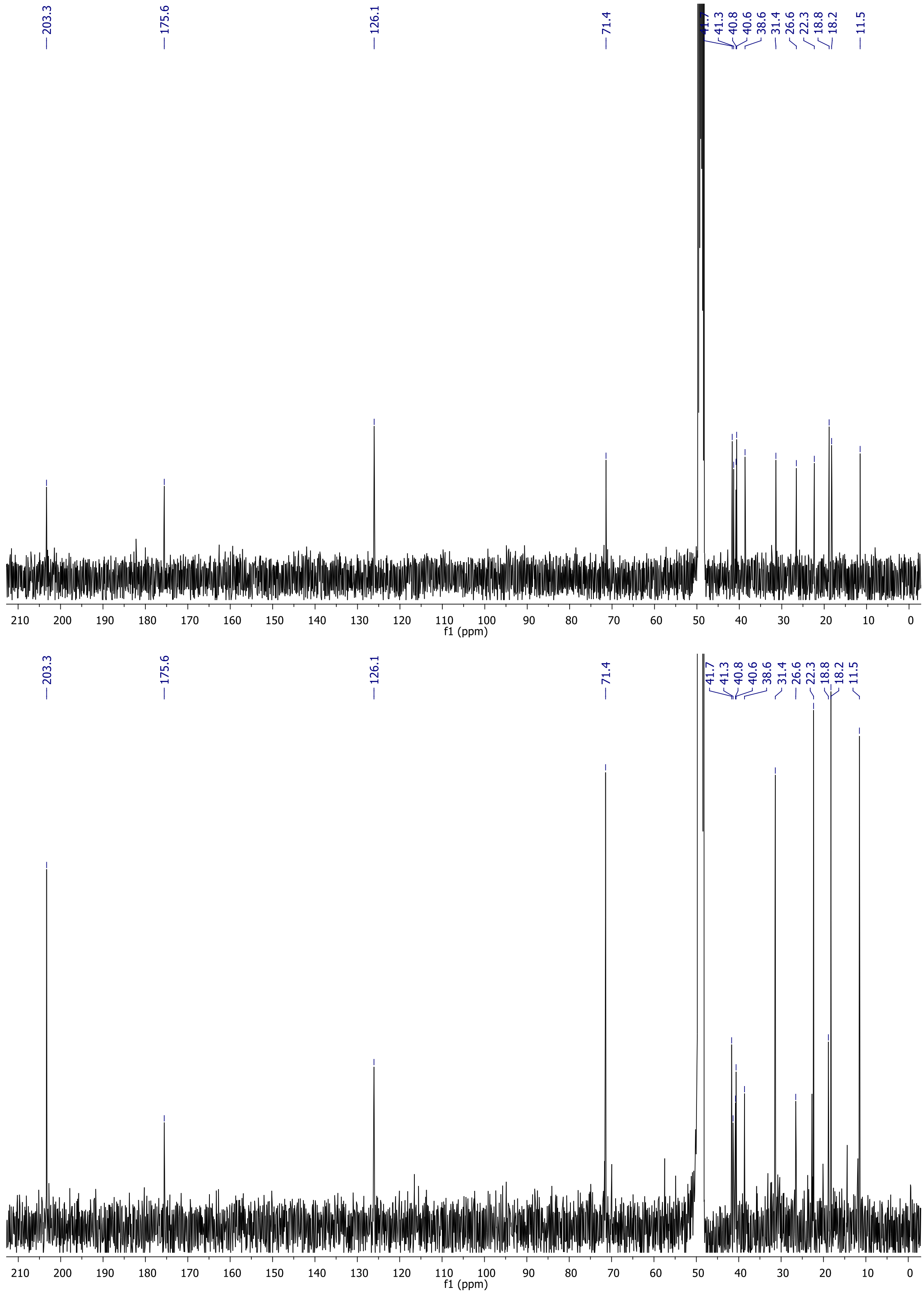

Figure S48. ^{13}C NMR spectra (125 MHz, CD_3OD) of compound **1** unlabeled (top) and labeled using glucose-1-^{13}C (bottom). Signals corresponding to carbons in compound **1** are assigned.

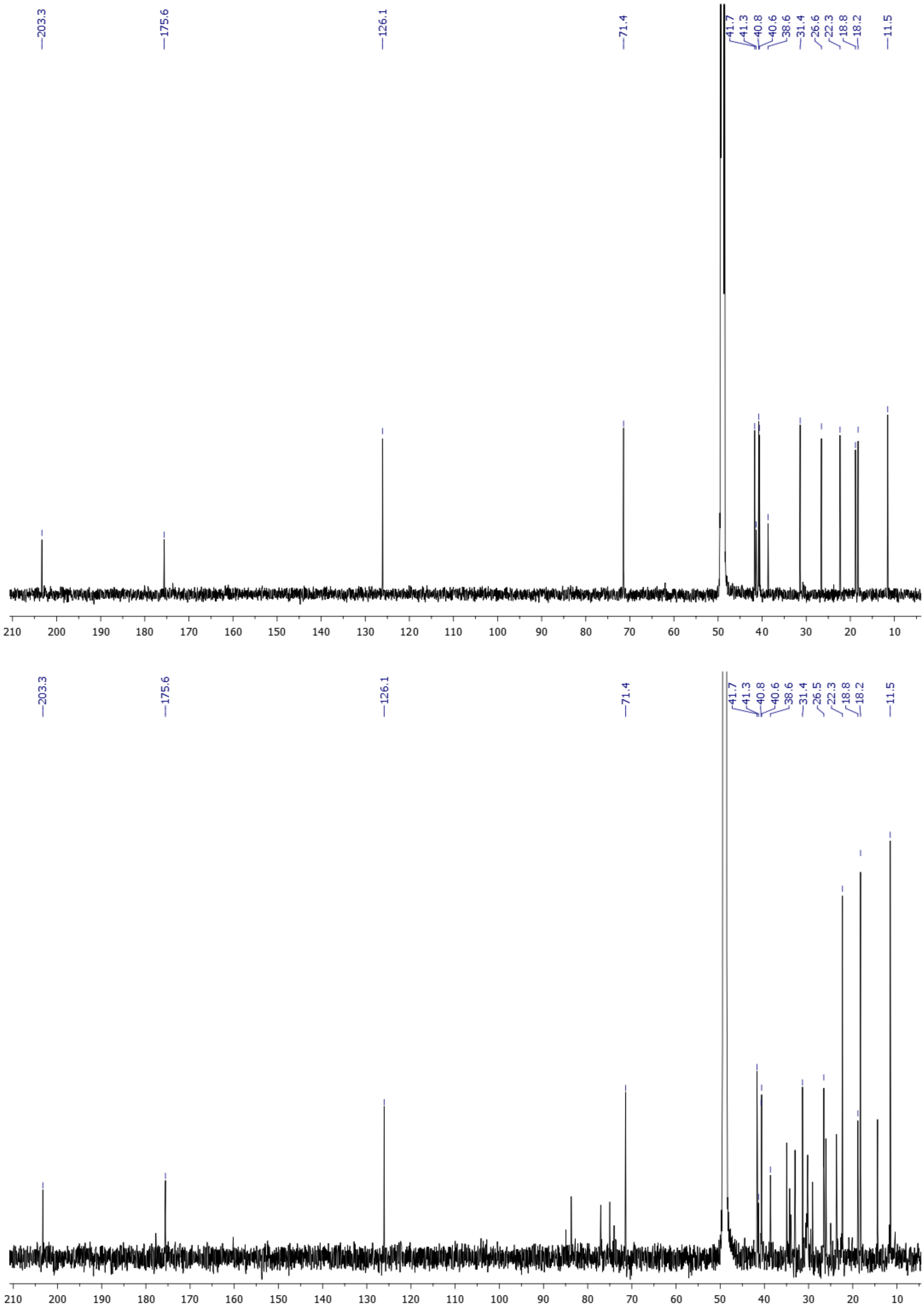

Figure S49. ^{13}C NMR spectra (125 MHz, CD_3OD) of compound **1** unlabeled (top) and labeled using pyruvate-3-^{13}C (bottom). Signals corresponding to carbons in compound **1** are assigned.

Table S9. Terpene putative BGCs identified by antiSMASH

AntiSMASH Assigned Region	GenBank Access Number	Terpene Related Gene	Most Similar Known BGC	Percentage of Genes Showing Similarity
Region 1.2	JJOH01000001	DF19_00385	Tetronasin (PKS)	9%
Region 5.2	JJOH01000005	DF19_35615	Geosmin	100%
Region 25.1	JJOH01000025	DF19_26680	Albaflavenone	100%
Region 56.1	JJOH01000092	DF19_41585	No match found	0%
Region 72.1	JJOH01000108	DF19_10595 to DF19_10620	Hopene	84%

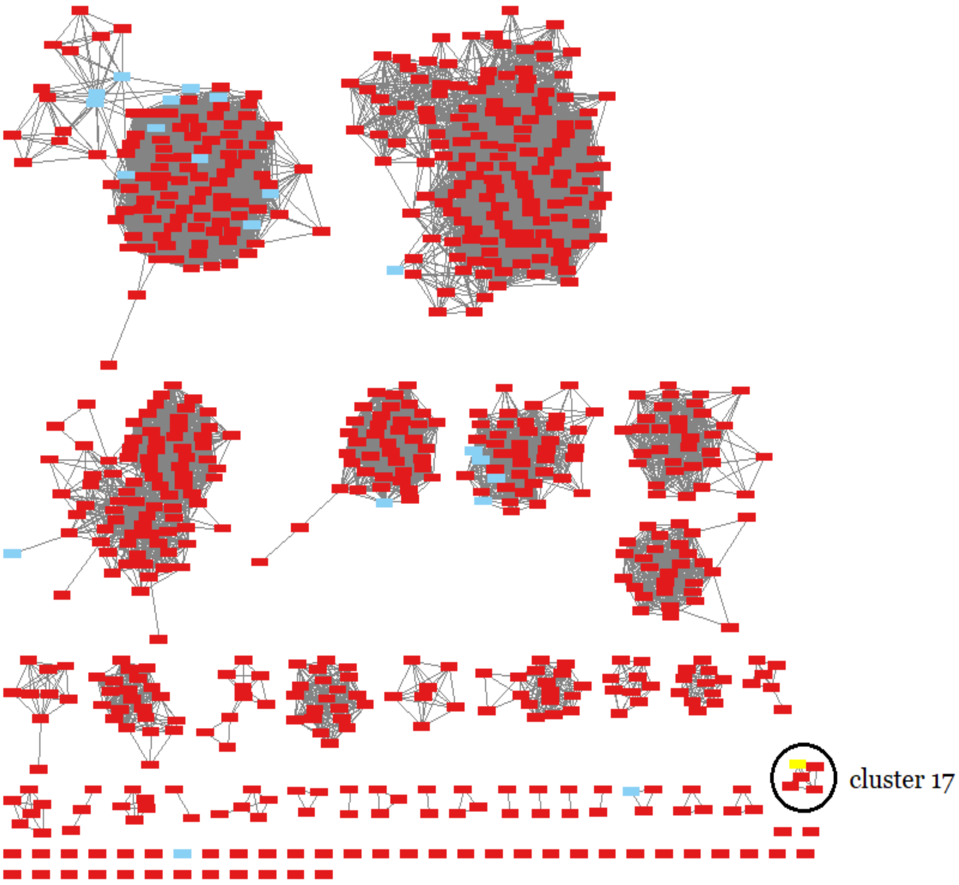

Figure S50. SSN using the putative TC KDN80181.1 (gene DF19_00380) from *S. olindensis* DAUFPE 5622 as input. Nodes in red belong to the IPR03920 PaxB-like family (an integral membrane cyclases, IMC), nodes in blue are proteins that were not previously annotated as IMC, and the yellow node in cluster 17 corresponds to KDN80181.1
